# Mechanochemical Release of Non‐Covalently Bound Guests from a Polymer‐Decorated Supramolecular Cage

**DOI:** 10.1002/anie.202102383

**Published:** 2021-05-05

**Authors:** Robin Küng, Tobias Pausch, Dustin Rasch, Robert Göstl, Bernd M. Schmidt

**Affiliations:** ^1^ Institut für Organische Chemie und Makromolekulare Chemie Heinrich-Heine-Universität Düsseldorf Universitätsstrasse 1 40225 Düsseldorf Germany; ^2^ DWI—Leibniz Institute for Interactive Materials Forckenbeckstrasse 50 52056 Aachen Germany; ^3^ Institute of Technical and Macromolecular Chemistry RWTH Aachen University Worringerweg 1 52074 Aachen Germany

**Keywords:** cage compounds, drug release, host–guest systems, mechanochemistry, polymers

## Abstract

Supramolecular coordination cages show a wide range of useful properties including, but not limited to, complex molecular machine‐like operations, confined space catalysis, and rich host–guest chemistries. Here we report the uptake and release of non‐covalently encapsulated, pharmaceutically‐active cargo from an octahedral Pd cage bearing polymer chains on each vertex. Six poly(ethylene glycol)‐decorated bipyridine ligands are used to assemble an octahedral Pd^II^_6_(TPT)_4_ cage. The supramolecular container encapsulates progesterone and ibuprofen within its hydrophobic nanocavity and is activated by shear force produced by ultrasonication in aqueous solution entailing complete cargo release upon rupture, as shown by NMR and GPC analyses.

## Graphical abstract

An octahedral Pd^II^_6_(TPT)_4_ cage with polymer chains on each vertex is activated by shear force from ultrasonication in aqueous solution. The metal–organic cage encapsulates progesterone or ibuprofen within its hydrophobic nanocavity and shows complete cargo release upon rupture.

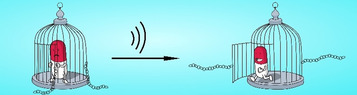

Metal‐mediated self‐assembly of organic ligands into discrete nanoscopic structures, such as cages^[1]^ and capsules^[2]^ (amongst other motifs),[^1c^, ^3^] has generated a large number of unique structures over the last decades. Some of these assemblies were shown to encapsulate guest compounds with high affinity,^[4]^ can be used as molecular transporters,^[5]^ or were used to modulate physicochemical properties inside the confined spaces within the assemblies.^[6]^ A variety of responsive systems have been reported using light[^7a^, ^7b^, ^7c^] or chemical stimuli as triggers.[^7d^, ^7e^]

Concomitantly, the mechanochemical activation of various metal−ligand bonds (metallocenes;^[8]^ Zn, Cu, Ni, and Rh complexes;[^9a^, ^9b^] N‐heterocyclic carbene complexes with Ag, Ru,[^9c^, ^9d^, ^9e^, ^9f^, ^9g^, ^9h^, ^9i^] and Cu;[^9j^, ^9k^, ^9l^, ^9m^] as well as Pd phosphanes^[9n]^) was successfully carried out. In addition, force activation of supramolecular rotaxanes bearing poly(methyl acrylate) (PMA) backbones was achieved by bond scission at the rotaxane junction,[^10a^, ^10c^, ^10d^] while on the other hand, catenanes are able to effectively distribute tensile deformation in macrocycles and can thus be considered a mechanical protecting group.^[10b]^

The mechanochemical release of cargo molecules from their respective carrier polymers is intrinsically challenging, as covalent chain scission generally results in the production of two shorter, but still polymeric, chain fragments. Methods cleverly circumventing this limitation led to proton release,^[11a]^ metal ion release following ferrocene rupture,^[11b]^ furan derivative release,[^11c^, ^11d^, ^11e^] or release from sophisticated polymer‐based microcapsules.^[11f]^ In most of these systems, inertial cavitation generated by ultrasound was the method to exert force on the solutions of the carrier polymers.

The release and activation of drugs by ultrasound was achieved in micelles, liposomes, or microbubbles,^[12]^ or by synergistically increasing drug efficacy.^[13]^ Recently, we established the ability of ultrasound in the context of polymer mechanochemistry,^[14]^ to activate force‐responsive molecular moieties (mechanophores)^[15]^ embedded in polymers to activate and release drugs.^[16]^ However, many of the above examples compromise their universal applicability by relying on strong and selective carrier–cargo interactions or even chemical modification of the cargo molecules.

Herein, we report the ultrasound‐induced disassembly of a cargo‐loaded self‐assembled supramolecular Pd^II^_6_(TPT)_4_ cage with the release of its nanoconfined guests. We demonstrate examples of several non‐covalently bound, completely unmodified and pharmaceutically active compounds (Figure 1) as cargo.

**Figure 1:**
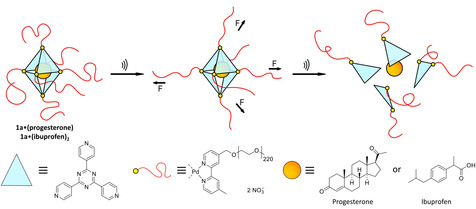
Schematic representation of the PEG‐functionalized octahedral cage **1 a**, bearing on average 220 repetitive ethylene glycol units at each vertex, leading to a total molar mass of 60 kDa. Activation by ultrasound in aqueous solution leads to fragmentation of the cage, releasing the non‐covalently bound cargo. The preloaded cargo is depicted as the orange dot within the cavity of cage **1 a**, representing either precisely one molecule of progesterone or two molecules of ibuprofen per supramolecular entity.

To enable the force‐induced scission of the Pd−N units within the Pd cage **1 a**, modified bipyridines were chosen as *cis*‐blocked, end‐capped ligands for the Pd corners. Therefore, 4‐bromomethyl‐4′‐methyl‐2,2′‐bipyridine was synthesized and poly(ethylene glycol) methyl ether (PEG, *M_n_*=10 kDa) was introduced by nucleophilic substitution, affording PEG‐functionalized bipyridine **9** (see the SI, Figure S2). PEG was specifically chosen for its water solubility, which is necessary to utilize the hydrophobic effect of the cage cavity. Over two steps, the corresponding Pd compound bearing nitrate counter anions **11** was obtained in 92 % yield. Adapting established procedures,^[1d]^ the PEG‐functionalized octahedral cage **1 a** was synthesized in aqueous solution by using six equivalents of the PEG‐functionalized Pd complex **11** and four equivalents of triazine **TPT**, giving access to the polymer‐embedded star‐shaped^[17]^ cage **1 a** in almost quantitative yields. ^1^H NMR of **1 a** in D_2_O showed the characteristic signals for the bipyridine and **TPT** panels, while the ethylene glycol repeat units and the methyl end groups were in accordance with the anticipated ratio (48:36:5330:18). Cage **1 a** was subsequently loaded with progesterone or ibuprofen, respectively, by adding excess drug to an aqueous solution of the cage. Guest uptake was again confirmed by ^1^H NMR in D_2_O by observing the distinctive shielding effect of the triazine panels on the encapsulated guests, leading to significant upfield shift of the peaks of around *δ*=1 ppm for both guests (Figure 2 c) and unambiguously confirming encapsulation within the cavity of cage **1 a**. Additionally, the encapsulation of both drugs and several other guests was carried out with a model compound **2**, without PEG units, thus allowing extensive NMR experiments including ^1^H DOSY and heteronuclear 2D measurements (see SI Figures S69–S74, S80–S98). Model compound **2** shows identical chemical shifts with guest compounds in ^1^H NMR and clearly indicates that each cavity encapsulates exactly one progesterone or two ibuprofen guests.

**Figure 2:**
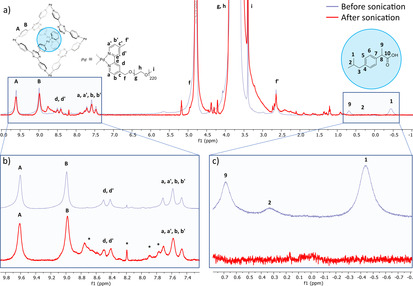
^1^H NMR (D_2_O, 298 K) of the encapsulated **1 a**⋅(**ibuprofen**)_2_ (blue line) and release of ibuprofen cargo (red line). Sonication time 3 h, a sequence of 1 s on and 1 s off was chosen, for each experiment only the “on” time is reported. The asterisks in the insets indicate the cage fragmentation products.

Subsequent sonication experiments of the cargo‐loaded cage **1 a** were performed using an immersion probe sonicator (20 kHz) in water. The release was monitored by ^1^H NMR and is shown in Figure 2 for **1 a**⋅(**ibuprofen**)_2_. The characteristic upfield guest signals of the isopropyl and methyl groups 1, 2, and 9 (*δ*=−0.45, 0.35 ppm, and 0.7 ppm), (Figure 2 c, blue line) completely disappeared over the course of the sonication experiment. We hypothesized that ibuprofen released from the cage precipitated from the aqueous solution concomitant with signals appearing corresponding to possible cage fragments (*δ*=8.79–8.60, 8.16, 7.88–7.75 ppm). Hence the release process was unequivocally connected to cage fragmentation. Yet, the fragmentation pathway remained unclear in that the guest molecules were either released from 1) damaged, partially intact cages or 2) the removal of one ligand induced complete disassembly of the overall cage structure.

Quantitative release was also observed for the sonication of **1 a**⋅(**progesterone**), where the methyl groups serve as an excellent probe to follow the release in the upfield ^1^H NMR, undisturbed by the broad PEG resonances (Figure S6). Additionally, the fragmentation of the Pd cage **1 a** not bearing cargo was observed on a similar time scale (Figure S5).

To examine the cargo release mechanism, **1 a**⋅(**ibuprofen**)_2_ was sonicated for only 15 min. Within this short period of time we reasoned that a considerably smaller fraction of the cage **1 a** would have the opportunity to fracture by inertial cavitation, but in principle still might have been able to release the guests due to an increased cavity size through ultrasound‐induced uncoiling of the polymers, facilitating guest “slippage”.^[18]^ As anticipated, cage **1 a** showed only minute amounts of fragmentation product, but at the same time, no cargo release was observed by ^1^H NMR (Figure S8). To unambiguously prove the mechanochemical origin of the observed release, model compound **2** bearing no polymer chains, and thus hypothesized not to be susceptible to force activation, was sonicated under identical conditions (Figure S11). As expected, cage **2** did not show any changes in the ^1^H NMR after sonication experiments. Furthermore, control sonication experiments using **2**⋅(**ibuprofen**)_2_, showed neither cargo release nor core fragmentation (Figure S12).

To probe the force‐induced degradation of the star‐shaped polymeric supramolecular assembly in more detail, samples before and after sonication of each **1 a**, **1 a**⋅(**ibuprofen**)_2_, and **1 a**⋅(**progesterone**) were analyzed by gel permeation chromatography (GPC) using CHCl_3_ as the eluent (Figure 3). The cages disassembled under most GPC measurement conditions because of interference from organic solvents and salts. While the molar mass distributions of the disassembled fragments before sonication matched those of the pristine PEG‐functionalized bipyridine **9** (Figure S13), a shoulder appeared at lower molar masses after sonication. This was attributed to non‐specific scission events of individual PEG chains either before or after the cage structure was already cleaved. Since the extent of observed non‐specific scission was only marginal compared to the observed quantitative mechanochemical release of the cargo molecules by NMR, we reasoned that the mechanochemically weakest link lay within the cage structure and not within the polymer chains, rendering the force activation of **1 a** reasonably selective.

**Figure 3:**
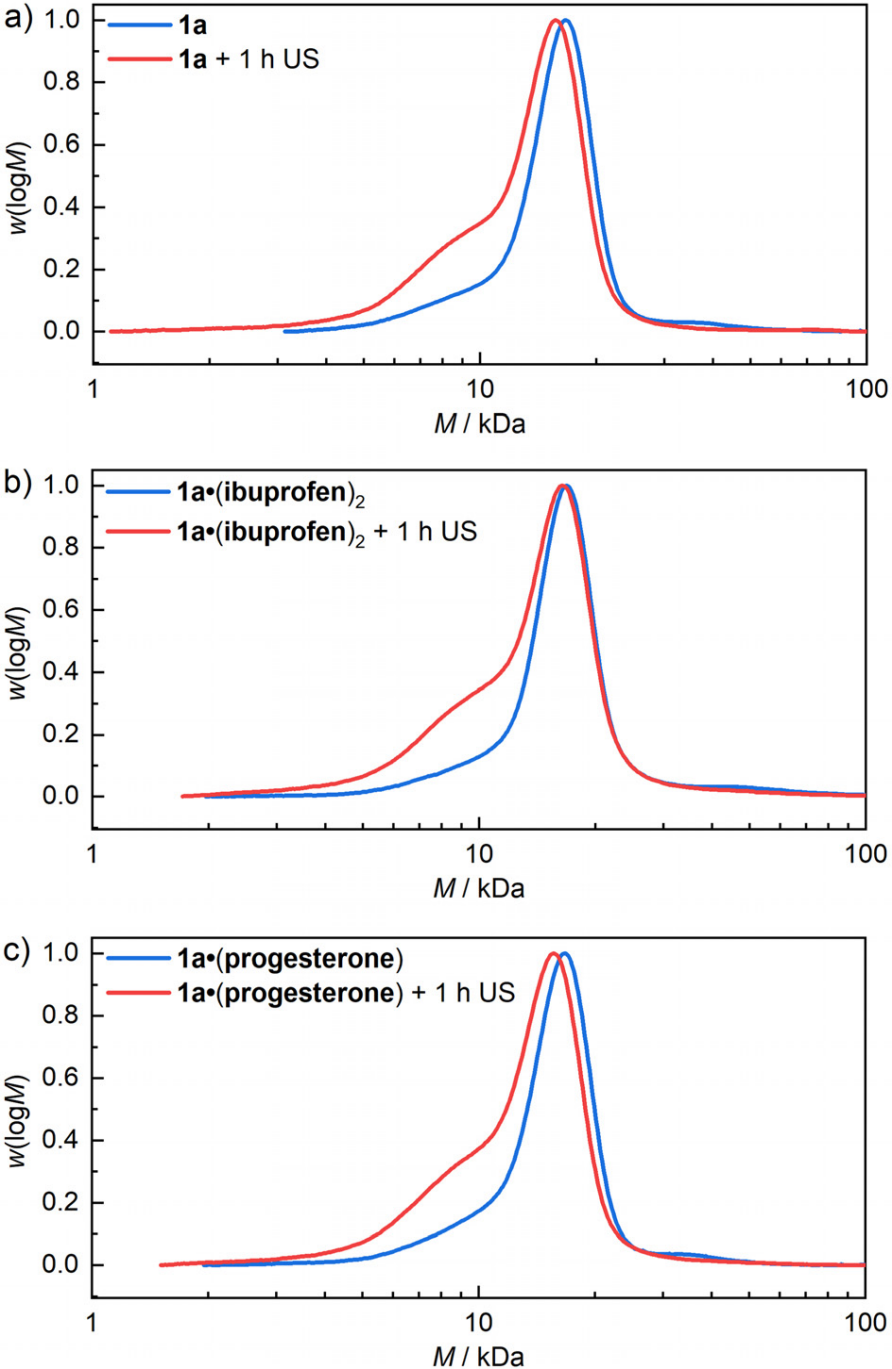
GPC chromatograms obtained in CHCl_3_ from the RI detector of cage **1 a** with and without guests before and after sonication for 1 h.

Next, we investigated whether an increase in the degree of polymerization of the attached PEG chains was also reflected in an increased tendency to release cargo. Therefore, we prepared an isostructural cage using PEG with *M_n_*=20 kDa at each vertex (Figure 4). Samples of the Pd cage **2** bearing no PEG chains, cage **1 a** bearing 220 repeat units at each ligand, and cage **1 b** bearing 440 repeat units at each ligand, were sonicated over the course of 1 h. Judging from ^1^H NMR integration, the anticipated molar mass dependency was observed, corresponding to a roughly 100 % increase in release rate when using 440 (cage **1 b**) instead of 220 repeat units (cage **1 a**, Figure 4).

**Figure 4:**
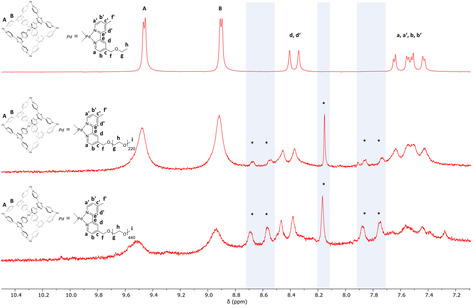
^1^H NMR (D_2_O, 298 K) of **2**, **1 a**, and **1 b** (from top to bottom), after 1 h of total sonication “on” time. The asterisks indicate the cage fragmentation. Only the aromatic region showing panels and bipyridine ligands is shown because of the intense and very broad resonances of the PEG chains.

In conclusion, we have presented the first example of a supramolecular coordination cage forming a star‐shaped, water‐soluble polymer structure which is responsive to ultrasonication‐induced shear force in solution. We showcased the mechanochemical release of both ibuprofen and progesterone from the same parent cage structure. Since the release of small molecules from their latent macromolecular carriers by means of polymer mechanochemistry generally requires specifically functionalized cargo molecules, we anticipate that our combination of universal supramolecular encapsulation and force as an external stimulus will contribute to the development of molecular release systems and potentially advanced therapeutics.

## Conflict of interest

The authors declare no conflict of interest.

## Supporting information

As a service to our authors and readers, this journal provides supporting information supplied by the authors. Such materials are peer reviewed and may be re‐organized for online delivery, but are not copy‐edited or typeset. Technical support issues arising from supporting information (other than missing files) should be addressed to the authors.

Supplementary
