# Supplementary material for: Mechanochemical Release of Non‐Covalently Bound Guests from a Polymer‐Decorated Supramolecular Cage
Source: Angew Chem Int Ed Engl. 2021 May 5;60(24):13626–30. doi: 10.1002/anie.202102383 (PMC8251918; doi:10.1002/anie.202102383)
Supplement: Supplementary file 1 — Supplementary [file ANIE-60-13626-s001.pdf]

Angewandte Chemie
Eine Zeitschrift der Gesellschaft Deutscher Chemiker

Supporting Information

Mechanochemical Release of Non-Covalently Bound Guests from a Polymer-Decorated Supramolecular Cage

Robin Küng, Tobias Pausch, Dustin Rasch, Robert Göstl, and Bernd M. Schmidt**

anie_202102383_sm_miscellaneous_information.pdf

Contents

General 3

Analytics 3

NMR measurements 3

DOSY NMR 3

IR 4

Mass spectrometry 4

Melting points 4

Gel permeation chromatography (GPC) 4

Experimental details 5

Synthesis 5

Synthesis of 2,4,6-tris(4-pyridyl)-1,3,5-triazine (**3**) 5

Synthesis of 4-hydroxymethyl-4'-methyl-2,2'-bipyridine (**4**) 6

Synthesis of 4-bromomethyl-4'-methyl-2,2'-bipyridine (**5**) 7

Synthesis of cage **2** 8

Synthesis of 4-ethoxymethyl-4'-methyl-2,2'-bipyridine (**6**) 8

Synthesis of (4-ethoxymethyl-4'-methyl-2,2'-bipyridine)-dichloropalladium(II) (**7**) 8

Synthesis of (4-ethoxymethyl-4'-methyl-2,2'-bipyridine)-dinitratopalladium(II) (**8**) 9

Synthesis of cage **2** 10

Synthesis of cage **1a** 11

Synthesis of 4-methoxypolyethyleneglycolmethyl-4'-methyl-2,2'-bipyridine (**9**) 11

Synthesis of (4-methoxypolyethyleneglycolmethyl-4'-methyl-2,2'-bipyridine)-dichloropalladium(II) (**10**) 12

Synthesis of (4-methoxypolyethyleneglycolmethyl-4'-methyl-2,2'-bipyridine)-dinitratopalladium(II) (**11**) 13

Synthesis of cage **1a** 14

Synthesis of cage **1b** 15

Synthesis of 4-methoxypolyethyleneglycolmethyl-4'-methyl-2,2'-bipyridine (**12**) 15

Synthesis of (4-methoxypolyethyleneglycolmethyl-4'-methyl-2,2'-bipyridine)-dichloropalladium(II) (**13**) 16

Synthesis of (4-methoxypolyethyleneglycolmethyl-4'-methyl-2,2'-bipyridine)-dinitratopalladium(II) (**14**) 17

Synthesis of cage **1b** 18

Encapsulation experiments 19

Guest encapsulations with cage **1a** 19

Cage **1a•(progesterone)** 19

Cage **1a•(ibuprofen)**$_2$ 20

Cage **1a•**(**phenolphthalein**) 20
Guest encapsulations with cage **2** 21
Cage **2•**(**progesterone**) 22
Cage **2•**(**ibuprofen**)$_2$ 22
Cage **2•**(**phenolphthalein**) 23
Cage **2•**(**testosterone**) 24
Cage **2•**(**drospirenone**) 24
Sonication experiments 25
Cage **1a** 26
Cage **1a•**(**progesterone**) 27
Cage **1a•**(**ibuprofen**)$_2$ 28
Cage **1a•**(**phenolphthalein**) 29
Cage **1b** 31
Cage **2** 31
GPC chromatogram in $CHCl_3$ 33
Spectra 34
Synthesis 34
2,4,6-Tris(4-pyridyl)-1,3,5-triazine 34
4-Hydroxymethyl-4’-methyl-2,2’-bipyridine 35
4-Bromomethyl-4’-methyl-2,2’-bipyridine 36
4-Ethoxymethyl-4’-methyl-2,2’-bipyridine 36
(4-Ethoxymethyl-4’-methyl-2,2’-bipyridine)-dichloropalladium(II) (**7**) 40
(4-Ethoxymethyl-4’-methyl-2,2’-bipyridine)-dinitratopalladium(II) (**8**) 43
Cage **2** 47
4-Methoxypolyethyleneglycolmethyl-4’-methyl-2,2’-bipyridine (**9**) 51
(4-Methoxypolyethyleneglycolmethyl-4‘-methyl-2,2‘-bibyridine)-dichloropalladium(II) (**10**) 52
(4-Methoxypolyethyleneglycolmethyl-4’-methyl-2,2’-bipyridine)-dinitratopalladium(II) (**11**) 53
Cage **1a** 54
4-Methoxypolyethyleneglycolmethyl-4’-methyl-2,2’-bipyridine (**12**) 55
(4-Methoxypolyethyleneglycolmethyl-4‘-methyl-2,2‘-bibyridine)-dichloropalladium(II) (**13**) 56
(4-Methoxypolyethyleneglycolmethyl-4’-methyl-2,2’-bipyridine)-dinitratopalladium(II) (**14**) 57
Cage **1b** 58
Spectra encapsulation and release studies 59
Cage **1a** 59
Cage **1b** 62
Comparison of spectra of cage **2•**(**progesterone**) & cage **1a•**(**progesterone**) before sonication and cage **1a•**(**progesterone**) after sonication 64

Comparison of spectra of **2•(ibuprofen)**$_2$ & **1a•(ibuprofen)**$_2$ before sonication (mid) and **1a•(ibuprofen)**$_2$ after sonication 70

Phenolphthalein 77

Testosterone 78

Drospirenone 81

References 84

General

Chemicals and solvents were purchased from Sigma-Aldrich, VWR/Merck, Tokyo Chemical Industry and were used without further purification. HPLC grade acetonitrile was used as received. Dichloromethane, tetrahydrofuran and dioxane were obtained by a solvent purification system from MBraun (SPS-800). Reactions were monitored by thin layer chromatography (TLC), using silica gel plates from Macherey Nagel (ALUGRAM® Xtra SIL G/UV254). Column chromatography was done with silica gel from Macherey Nagel (Silica 60 M, 0.04-0.063 mm). The eluents are stated individually for each reaction. If stated that the silica gel was deactivated, 1-2 % of NEt_3 was added to the eluent and the column was flushed three times. The solvents were removed under reduced pressure by using a rotary evaporator at 50 °C, if not stated otherwise.

Reactions which had to be done under complete exclusion of water were prepared by drying the laboratory glassware and the stirring bar at 80 °C for several hours. Reactions were run using Schlenk techniques for working under an inert atmosphere of nitrogen or argon.

Analytics

NMR measurements

The measurements of ^{1}H NMR-, ^{13}C{^{1}H}-NMR-spectra, DOSY and 2D-spectra were recorded on a Bruker Avance III – 300 (^{1}H NMR: 300 MHz, ^{13}C{^{1}H}-NMR: 75 MHz) and Bruker Avance III – 600 (^{1}H NMR: 600 MHz, ^{13}C{^{1}H}-NMR: 150 MHz) NMR-spectrometers. ^{13}C{^{1}H}-NMR of the encapsulation complexes were measured on a Bruker Avance III equipped with a BBO H&F cryoprobe at the Max Planck Institute for Coal Research located in Mülheim an der Ruhr, Germany. All samples were dissolved in deuterated solvents.

DOSY NMR

For the estimation of the hydrodynamic radii, the unmodified Stokes-Einstein-equation was used.[1]

$$D = \frac{k_B T}{6\pi\eta r_H}$$

D is the measured diffusion coefficient (m^2s^{-1})

k_B is the Boltzmann constant ($1.3806485 \cdot 10^{-23}$ $m^2 kg\ s^{-2}\ K^{-1}$)

T is the temperature (K)

r_H is the hydrodynamic radius of the analyte (m)

η is the viscosity of the solvent at temperature T ($kg\ m^{-1}\ s^{-1}$)

IR

Infrared spectra were measured with a FT/IR-6200 of the company JASCO and FT/IR IRAffinity-1 with ATR attachment of the company Shimadzu.

Mass spectrometry

Mass spectrometry was performed by using a UHR-QTOF maXis 4G spectrometer of the company Bruker Daltonik.

Melting points

Melting points were determined with a melting point apparatus (B-540) of the company BÜCHI Labortechnik GmbH.

Gel permeation chromatography (GPC)

GPC (SEC) with $CHCl_3$ (≥99.8%, stabilized with 2-methyl-2-buten, HiPerSolv CHROMANORM® HPLC grade, VWR) as eluent was performed using a HPLC pump (PU-2080plus, Jasco) equipped with a refractive index detector (RI-2031plus, Jasco). The sample solvent contained 250 $mg \cdot mL^{-1}$ 3,5-di-*t*-4-butylhydroxytoluene (BHT, ≥99%, Fluka) as internal standard. One pre-column (8×50 mm) and four SDplus gel columns (8×300 mm, SDplus, MZ Analysentechnik) were applied at a flow rate of 1.0 $mL \cdot min^{-1}$ at 20 °C. The diameter of the gel particles was 5 µm, the nominal pore widths were 50, 10^2, 10^3, and 10^4 Å. Calibration was achieved using narrowly distributed poly(methyl methacrylate) standards (Polymer Standards Service). Molar masses (M_n and M_w) and molar mass distributions (M_w/M_n) were calculated by using the PSS WinGPC UniChrom software (Version 8.1.1).

Experimental details

Synthesis

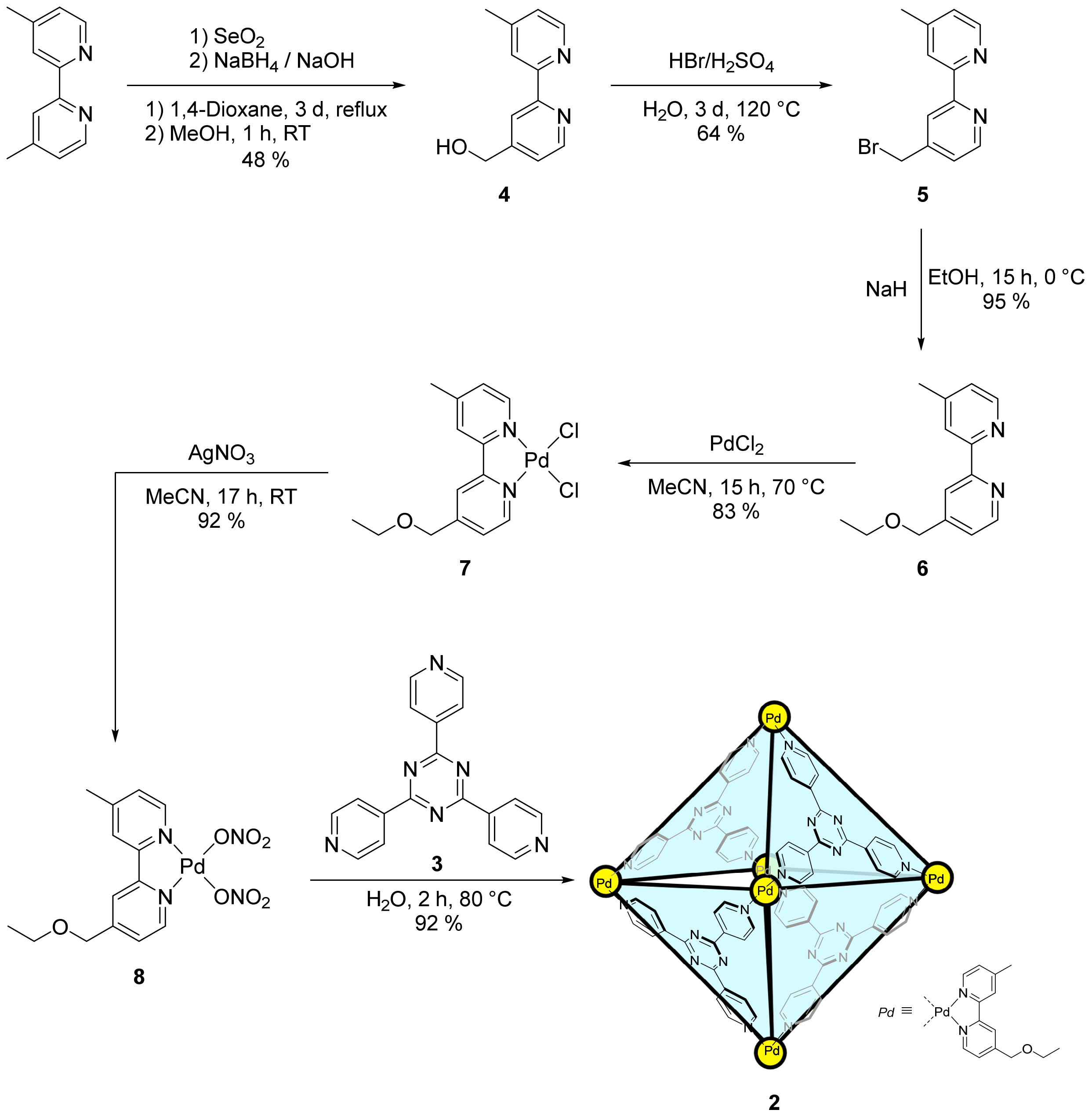

*Figure S 1: Synthetic overview of cage **2**.*

*Synthesis of 2,4,6-tris(4-pyridyl)-1,3,5-triazine (**3**)*

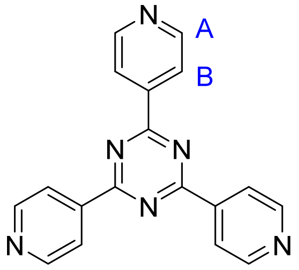

This synthesis was done in accordance to the literature procedure published by Fujita *et al*.[2]

4-Cyanopyridine (10 g, 100 mmol, 1.0 eq.) was stirred at 160 °C. Powdered NaOH (0.40 g, 10 mmol, 0.10 eq.) was added and the reaction was stirred overnight for 18 h. The reaction was cooled to RT, washed with acetone, and dried. The resulting solid was dissolved in 2 M HCl (80 mL) and precipitated with a 5 M NaOH-solution (80 mL). The white solid was filtered, washed with H_2O and acetone, dried in a high vacuum oven at 80 °C yielding the product as an off-white solid (4.5 g 45 %). If needed the product was sublimated (230 °C, 1 x 10^{-3} mbar).

^{1}H NMR (300 MHz, $CDCl_3$): δ = 8.97 (d, *J* = 5.8 Hz, 6H, **A**), 8.62 (s, 6H, **B**).

Synthesis of 4-hydroxymethyl-4'-methyl-2,2'-bipyridine (4)

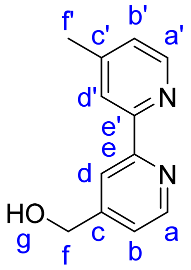

This synthesis was done in accordance to the literature procedure published by Studer *et al*.[3]

4,4'-Dimethyl-2,2'-bipyridine (5.0 g, 27 mmol, 1.0 eq.) was dissolved in 1,4-dioxane (180 mL) and SeO_2 (3.3 g, 30 mmol, 1.1 eq.) was added. This suspension was degassed with argon for 30 min. and then refluxed for 3 d. After the reaction was cooled to room temperature the black solid was filtered off and washed several times with chloroform giving a yellow solution. The solvent was removed under reduced pressure and the resulting pink solid was suspended in MeOH (40 mL). $NaBH_4$ (1.1 g, 30 mmol, 1.1 eq.) dissolved in NaOH (0.2 M, 6.8 mL, 14 mmol) was added to the suspension at 0 °C. The mixture was stirred for 1 h at RT. The resulting solution was set to pH = 1 with aqueous HCl and stirred for additional 30 min. The red suspension was adjusted to pH = 9 with a saturated Na_2CO_3-solution and extracted with DCM (3 x 100 mL). The combined organic phases were dried over $MgSO_4$ and the solvent was removed under reduced pressure. The crude product was purified by a deactivated silica gel chromatography with cyclohexane/ethyl acetate (9/1, later 1/1) yielding **4** as a colourless solid (2.6 g, 48 %).

^{1}H NMR (300 MHz, $CDCl_3$): δ 8.60 (d, *J* = 5.0 Hz, 1H, **a**), 8.51 (d, *J* = 5.0 Hz, 1H, **a´**), 8.33 (s, 1H, **d**), 8.20 (s, 1H, **d´**), 7.30 (d, *J* = 5.0 Hz, 1H, **b**), 7.14 (d, *J* = 5.1 Hz, 1H, **b´**), 4.78 (s, 2H, **f**), 3.10 (s, 1H, **g**), 2.43 (s, 3H, **f´**).

Synthesis of 4-bromomethyl-4'-methyl-2,2'-bipyridine (5)

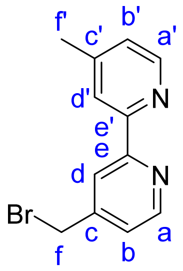

This synthesis was done in accordance to the literature procedure published by Studer *et al.*[3]

4-Hydroxymethyl-4'-methyl-2,2'-bipyridine (**4**) (2.0 g, 10 mmol, 1.0 eq.) was dissolved in HBr/H_2O (48 wt%, 100 mL). H_2SO_4 (4 mL) was added and the reaction mixture was heated to 120 °C for 3 d. Water (25 mL) was added and the reaction was basified with Na_2CO_3 to pH = 8. The aqueous solution was extracted with DCM (8 x 50 mL) until the organic layer was colourless, dried over Na_2SO_4 and filtered. The solvent was removed under reduced pressure at 30 °C. The crude product was purified by silica gel chromatography with dichloromethane/acetone (1/1) yielding **5** as a colourless solid (1.7 g, 64 %) (again the solvent was removed under reduced pressure at 30 °C).

^{1}H NMR (300 MHz, $CDCl_3$): δ 8.59 (d, *J* = 5.0 Hz, 1H, **a**), 8.48 (d, *J* = 5.0 Hz, 1H, **a´**), 8.36 (s, 1H, **d**), 8.18 (s, 1H, **d´**), 7.28 (dd, *J* = 5.0, 1.8 Hz, 1H, **b**), 7.10 (d, *J* = 5.0 Hz, 1H, **b´**), 4.41 (s, 2H, **f**), 2.38 (s, 3H, **f´**).

Synthesis of cage 2

Synthesis of 4-ethoxymethyl-4’-methyl-2,2’-bipyridine (6)

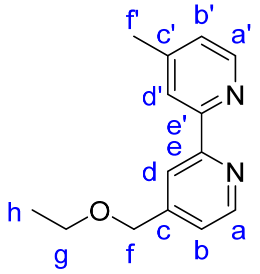

4-Bromomethyl-4’-methyl-2,2’-bipyridine (**5**) (0.55 g, 2.1 mmol, 1.0 eq.) was dissolved in EtOH (10 mL) a solution of NaH (60 wt% in mineral oil, 0.20 g, 5.1 mmol, 2.4 eq.) in EtOH (15 mL) was added in small portions. The resulting solution was stirred for 15 h until the starting material was fully converted, after that H_2O (15 mL) was added. The aqueous solution was extracted with EE (8 x 15 mL) until the organic layer was colourless and the combined organic layers were dried over anhydrous Na_2SO_4. The solution was filtered, and the solvent was removed under reduced pressure. The obtained brown oil was purified by silica gel column chromatography with DCM/MeOH (97/3) to yield **6** (0.45 g, 95 %) as a pale-yellow oil.

^{1}H NMR (600 MHz, $CDCl_3$): δ 8.61 (d, *J* = 4.9 Hz, 1H, **a**), 8.51 (d, *J* = 4.9 Hz, 1H, **a´**), 8.30 (s, 1H, **d**), 8.21 (s, 1H, **d´**), 7.32 (d, *J* = 4.6 Hz, 1H, **b**), 7.10 (d, *J* = 4.7 Hz, 1H, **b´**), 4.57 (s, 2H, **f**), 3.57 (q, *J* = 7.0 Hz, 2H, **g**), 2.41 (s, 3H, **f´**), 1.26 (t, *J* = 7.0 Hz, 3H, **h**); **^{13}C{^{1}H} NMR** (151 MHz, $CDCl_3$): δ 156.33 (**e**), 155.90 (**e´**), 149.34 (**c**), 149.05 (**c´**), 149.02 (**a**), 148.25 (**a´**), 124.82 (**b´**), 122.11 (**b**), 121.92 (**d´**), 119.47 (**d**), 71.27 (**f**), 66.49 (**g**), 21.26 (**f´**), 15.26 (**h**). **IR** (cm^{-1}): 3053.32 (w), 2974.23 (w), 2866.22 (b), 1597.06 (s), 1556.55 (m), 1456.26 (m), 1379.10 (m), 1109.07 (s,b), 991.41 (m), 821.68 (s); **HRMS** (ESI): m/z calc. 228.1263; found 229.1340 $[M+H]^+$.

Synthesis of (4-ethoxymethyl-4’-methyl-2,2’-bipyridine)-dichloropalladium(II) (7)

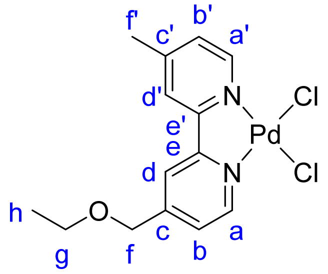

4-Ethoxymethyl-4’-methyl-2,2’-bipyridine (**6**) (0.76 g, 3.3 mmol, 1.0 eq.) was dissolved in MeCN (22 mL) and $PdCl_2$ (0.59 g, 3.3 mmol, 1.0 eq.) was added, resulting in a brown suspension which was heated at 70 °C for 15 h until the suspension turned yellow. The suspension was allowed to cool to

room temperature. The solid was filtered off and washed with cold H_2O (3 x 3 mL), cold acetone (6 × 3 mL) and dried *in vacuo* at 40 °C for 3 h yielding **7** as a yellow solid (1.1 g, 83 %).

^{1}H NMR (600 MHz, DMSO-d_6): δ 9.03 (d, *J* = 5.9 Hz, 1H, **a**), 8.92 (d, *J* = 5.9 Hz, 1H, **a´**), 8.47 (s, 1H, **d**), 8.43 (s, 1H, **d'**), 7.78 – 7.70 (m, 1H, **b**), 7.67 – 7.58 (m, 1H, **b´**), 4.70 (s, 2H, **f**), 3.61 (q, *J* = 7.0 Hz, 2H, **g**), 2.53 (s, 3H, **f´**), 1.23 (t, *J* = 7.0 Hz, 3H, **h**); **^{13}C{^{1}H} NMR** (126 MHz, DMSO-d_6): δ 156.03 (**e**), 155.55 (**e´**), 153.75 (**c**), 153.28 (**c´**), 149.35 (**a**), 148.75 (**a´**), 127.63 (**b´**), 124.44 (**d´**), 124.36 (**b**), 121.11 (**d**), 69.26 (**f**), 65.88 (**g**), 20.79 (**f´**), 14.84 (**h**); **IR** (cm^{-1}): 3115.04 (w), 3064.89 (w), 2927.94 (w), 1614.42 (m), 1419.61 (m), 1347.89 (w), 1303.88 (w), 1244.09 (w), 1163.08 (w), 1091.71 (s), 866.04 (s), 829.39 (s), 746.45 (w), 644.22 (w); **Elemental analysis:** calc. for $C_{14}H_{16}Cl_2N_2OPd$ %C: 41.46, %H: 3.98, %N: 6.91; found %C: 41.64, %H: 3.90, %N: 6.95; **Mp.**: 243.7-245.3 °C.

Synthesis of (4-ethoxymethyl-4'-methyl-2,2'-bipyridine)-dinitratopalladium(II) (8)

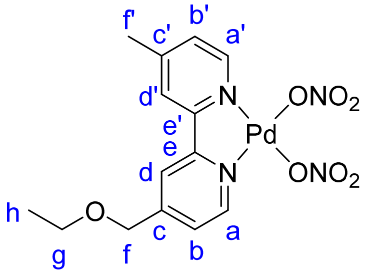

Nitrate complex **8** was prepared by adding $AgNO_3$ (0.43 g, 2.5 mmol, 2.0 eq.) to a suspension of (4-ethoxymethyl-4'-methyl-2,2'-bipyridine)-dichloropalladium(II) (**7**) (0.51 g, 1.3 mmol, 1.0 eq.) in MeCN (45 mL). The round-bottomed flask was wrapped in aluminium foil and the suspension was stirred for 17 h in the dark. The precipitated AgCl was removed by centrifugation (4400 rpm, 20 min), the resulting yellow supernatant was transferred into a round bottom flask and the solvent was removed by rotary evaporation to yield **8** as a yellow solid (0.53 g, 92 %).

^{1}H NMR (600 MHz, DMSO-d6): δ 8.48 (s, 1H, **d**), 8.41 (s, 1H, **d´**), 8.13 (d, *J* = 6.0 Hz, 1H, **a**), 8.01 (d, *J* = 6.0 Hz, 1H, **a´**), 7.71 (d, *J* = 6.0, 1H, **b**), 7.63 (d, *J* = 6.3, 1H, **b´**), 4.72 (s, 2H, **f**), 3.61 (q, *J* = 7.0 Hz, 2H, **g**), 2.55 (s, 3H, **f´**), 1.23 (t, *J* = 7.0 Hz, 3H, **h**); **^{13}C{^{1}H} NMR** (151 MHz, DMSO-d6): δ 155.80 (**e**), 155.54 (**c**), 155.29 (**e´**), 155.17 (**c´**), 148.61 (**a**), 148.00 (**a´**), 128.33 (**b**), 125.04 (**d**), 124.97 (**b´**), 121.55 (**d´**), 69.23 (**f**), 66.10 (**g**), 21.06 (**f´**), 15.01 (**h**). **IR** (cm^{-1}): 3122.75 (w), 2968.45 (w), 2926.01 (w), 2767.85 (w), 1614.42 (m), 1494.83 (s, b), 1257.59 (s, b), 1122.57 (m), 972.12 (s), 898.83 (m), 833.25 (m); **Elemental analysis:** calc. for $C_{14}H_{16}N_4O_7Pd$ %C: 36.66, %H: 3.52, %N: 12.21; found %C: 36.44, %H: 3.28, %N: 12.06; **Mp.**: 248.2-249.4 °C.

Synthesis of cage 2

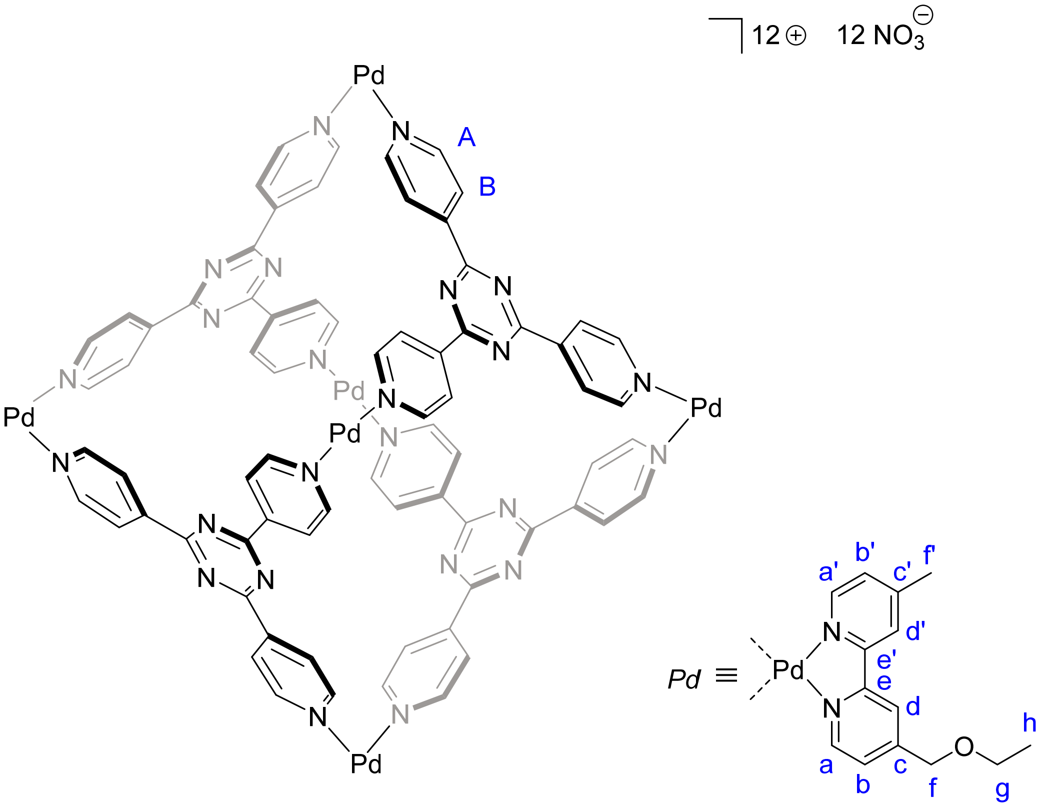

A suspension of (4-ethoxymethyl-4′-methyl-2,2′-bipyridine)-dinitratopalladium(II) (**8**) (0.30 g, 0.66 mmol, 1.0 eq.) and 2,4,6-tris(4-pyridyl)-1,3,5-triazine (**3**) (0.14 g, 0.44 mmol, 0.67 eq.) in H_2O (22 mL) was stirred at 80 °C for 2 h. Trace amounts of insoluble materials were removed by filtration using a syringe filter. The obtained clear yellow solution was evaporated under reduced pressure to give cage **2** (0.40 g, 10 mmol, 92 %) as a pale-yellow solid.

^{1}H NMR (600 MHz, D_2O): δ 9.48 (d, *J* = 6.2 Hz, 24H, **A**), 8.92 (d, *J* = 6.2 Hz, 24H, **B**), 8.42 (s, 6H, **d**), 8.36 (s, 6H, **d´**), 7.66 (d, *J* = 6.0 Hz, 6H, **a**), 7.57 (d, *J* = 6.0 Hz, 6H, **b**), 7.53 (d, *J* = 6.0 Hz, 6H, **a´**), 7.45 (d, *J* = 6.1 Hz, 6H, **b´**), 4.86 (s, 12H, **f**), 3.75 (q, *J* = 7.1 Hz, 12H, **g**), 2.63 (s, 18H, **f´**), 1.30 (t, *J* = 7.1 Hz, 18H, **h**); **^{13}C{^{1}H} NMR** (126 MHz, D_2O): δ 169.84 (**D**), 157.03 (**e´**), 156.67 (**c´**), 156.06 (**e**), 155.63 (**c**), 152.52 (**A**), 150.09 (**a**), 149.45 (**a´**), 146.43 (**C**), 128.98 (**b´**), 126.74 (**B**), 125.79 (**b**), 125.35 (**d´**), 122.08 (**d**), 69.90 (**f**), 67.50 (**g**), 21.17 (**f´**), 14.44 (**h**); **DOSY**: D= $2.16 \cdot 10^{-10}$ m^2/s **IR** (cm^{-1}): 3093.82 (w), 3427.51 (w), 3028.24 (w), 2821.86 (w), 2358.94 (w), 1622.13 (m), 1506.41 (s), 1317.38 (s,b), 1111.00 (m), 974.05 (w), 810.10 (s); **Mp.**: 298.9-301.2 °C (dec.).

Synthesis of cage 1a

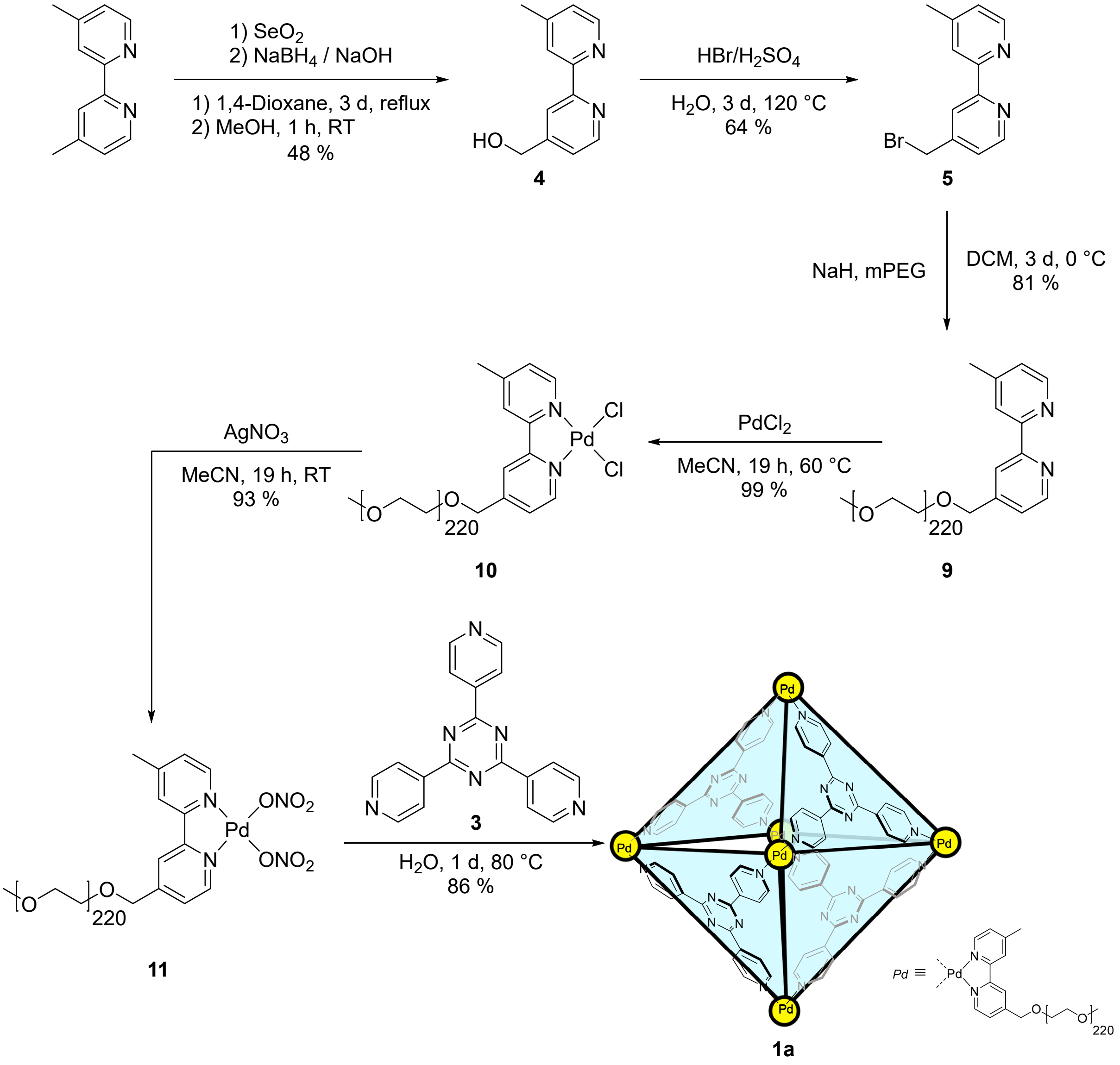

*Figure S 2: Synthetic overview of cage **1a**.*

Synthesis of 4-methoxypolyethyleneglycolmethyl-4'-methyl-2,2'-bipyridine (9)

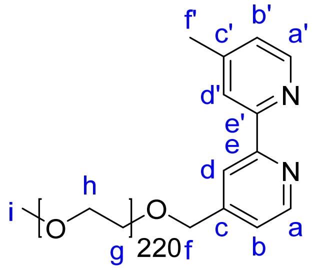

Poly(ethylene glycol) methyl ether (M_n = 10.000 Da, 5.0 g, 0.50 mmol, 1.0 eq.) was degassed with N_2 and dissolved in DCM (40 mL). The colourless solution was cooled to 0 °C. NaH (80 mg, 1.0 mmol, 4.0 eq.) was added and the reaction mixture was stirred for 1 h. 4-Bromomethyl-4'-methyl-2,2'-

bipyridine (**5**) (0.14 g, 0.53 mmol, 1.1 eq.) was added and the mixture was stirred for 3 d at 0 °C before the reaction was quenched with a saturated NH_4Cl-solution (2 mL). The reaction was stirred for an additional hour and then filtered. Water (50 mL) and a saturated NH_4Cl-solution (5 mL) were added, and the aqueous phase was extracted with DCM (3 x 50 mL). The combined organic phases were dried over $MgSO_4$, filtered and the solvent was removed under reduced pressure. The crude product was dialysed against deionized water for 4 d (MWCO: 1 kDa). After the solvent was removed the white solid was dissolved in hot DCM and precipitated in cold diethyl ether. After filtration **9** was obtained as a colourless solid (4.1 g, 81 %).

^{1}H NMR (600 MHz, $CDCl_3$): δ 8.62 (d, *J* = 5.1 Hz, 1H, **a**), 8.52 (d, *J* = 5.1 Hz, 1H, **a'**), 8.37 (s, 1H, **d**), 8.28 (s, 1H, **d'**), 7.39 (s, 1H, **b**), 7.17 (s, 1H, **b'**), 4.65 (s, 2H, **f**), 3.73 – 3.45 (m, 1072H, **g**, **h**), 3.32 (s, 3H, **i**), 2.44 (s, 3H, **f'**); **IR** (cm^{-1}): 2881.65 (m), 1465.90 (m), 1359.82 (w), 1340.53 (s), 1278.81 (m), 1242.16 (m), 1147.65 (m), 1099.43 (s), 1060.85 (m), 960.55 (m), 840.96 (m). **Mp.**: 60-65 °C.

Synthesis of (4-methoxypolyethyleneglycolmethyl-4'-methyl-2,2'-bipyridine)-dichloropalladium(II) (10)

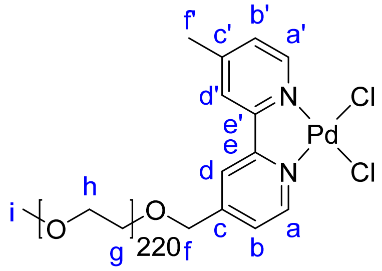

4-Methoxypolyethyleneglycolmethyl-4'-methyl-2,2'-bipyridine (**9**) (3.8 g, 0.37 mmol, 1.0 eq.) and $PdCl_2$ (66 mg, 0.37 mmol, 1.0 eq.) were suspended in MeCN (45 mL) and heated to 60 °C. After 19 h the yellow solution was cooled to RT, filtered and the solvent was removed under reduced pressure yielding the product as a yellow solid (3.8 g, 99 %).

^{1}H NMR (600 MHz, $CDCl_3$): δ 9.01 (d, *J* = 5.8 Hz, 1H, **a**), 8.93 (d, *J* = 5.8 Hz, 1H, **a'**), 8.12 (s, 1H, **d**), 7.97 (s, 1H, **d'**), 7.39 (d, *J* = 5.9 Hz, 1H, **b**), 7.24 (d, *J* = 6.0 Hz, 1H, **b'**), 4.76 (s, 2H, **f**), 3.59 (s, 911H, **g**, **h**), 3.32 (s, 3H, **i**), 2.55 (s, 3H, **f'**); **IR** (cm^{-1}): 2881.65 (m), 2360.87 (w), 1465.90 (m), 1359.82 (w), 1340.53 (s), 1278.81 (m), 1240.23 (m), 1147.65 (m), 1097.50 (s), 1060.85 (m), 960.55 (m), 840.96 (m); **Mp.**: 60-65 °C.

*Synthesis of (4-methoxypolyethyleneglycolmethyl-4’-methyl-2,2’-bipyridine)-dinitratopalladium(II) (**11**)*

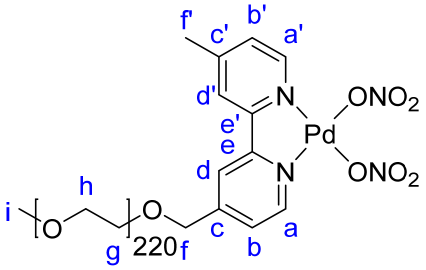

(4-methoxypolyethyleneglycolmethyl-4’-methyl-2,2’-bipyridine)-dichloro-palladium (**10**) (4.1 g, 0.40 mmol, 1.0 eq.) was dissolved in MeCN (50 mL) and $AgNO_3$ (0.15 g, 0.88 mmol, 2.2 eq.) was added, resulting in a colour change from yellow to a pale yellow. The conversion was tracked with 1 mL NMR-samples. After 19 h a full conversion of the starting material was observed and the AgCl was removed by centrifugation (4400 rpm, 1 h). The resulting yellow supernatant was transferred into a round bottom flask and the solvent was removed by rotary evaporation at 30 °C in the dark to yield a yellow solid. This solid was dissolved in DCM, precipitated from cold diethyl ether, filtered, and dried to yield **11** as a yellow solid (3.8 g, 93 %).

^{1}H NMR (600 MHz, $CDCl_3$): δ 8.22 (s, 1H, **d**), 8.11 (d, *J* = 5.9 Hz, 1H, **a**), 8.09 (s, 1H, **d’**), 8.02 (d, *J* = 5.9 Hz, 1H, **a’**), 7.55 (d, *J* = 6.0 Hz, 1H, **b**), 7.36 (d, *J* = 6.0, 1.7 Hz, 1H, **b’**), 4.79 (s, 2H, **f**), 3.57 (m, 985H, **g**, **h**), 3.30 (s, 3H, **i**), 2.56 (s, 3H, **f**); **IR** (cm^{-1}): 2881.65 (m), 1465.90 (m), 1359.82 (w), 1340.53 (s), 1278.81 (m), 1240.23 (m), 1147.65 (m), 1097.50 (s), 1060.85 (m), 960.55 (m), 840.96 (m); **Mp.**: 60-65 °C.

*Synthesis of cage **1a***

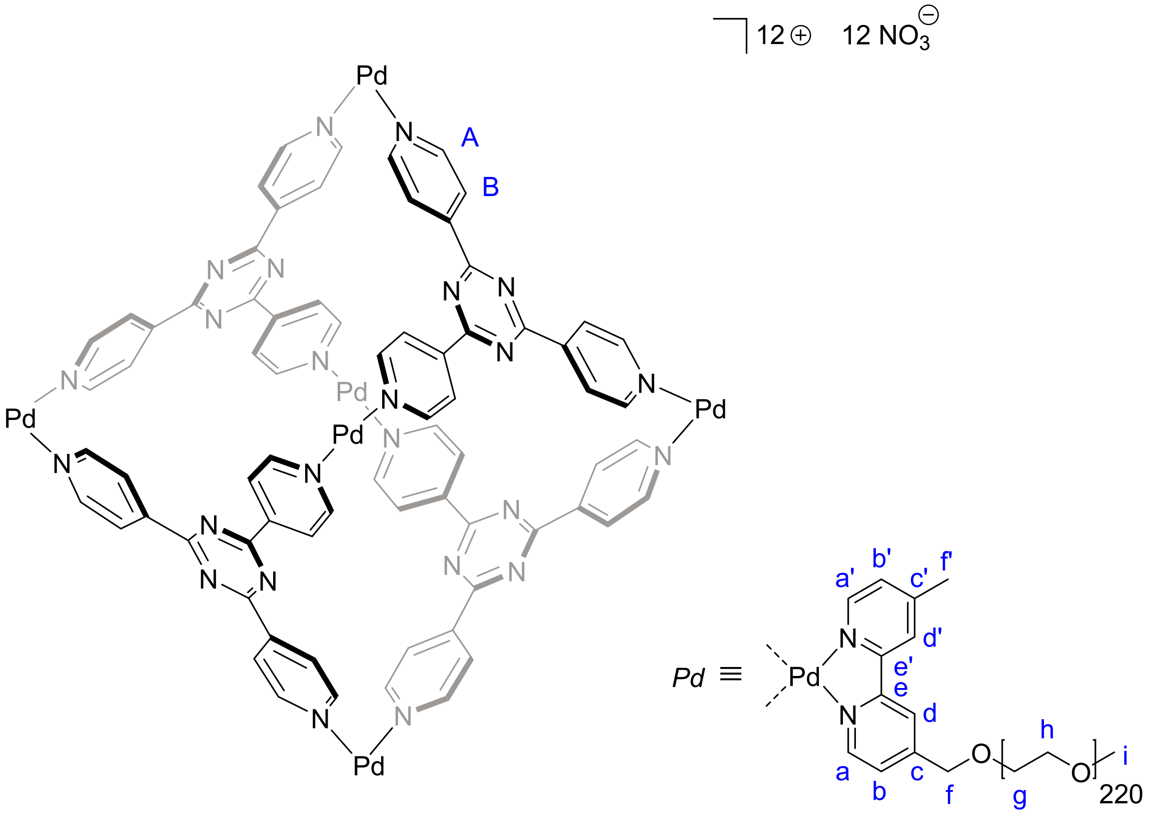

(4-methoxypolyethyleneglycolmethyl-4'-methyl-2,2'-bipyridine)-dinitrato palladium (**11**) (0.33 g, 0.032 mmol, 1.0 eq.) and 2,4,6-tris(4-pyridyl)-1,3,5-triazine (**3**) (9.6 mg, 0.021 mmol, 0.67 eq.) were suspended in H_2O (0.80 mL). The reaction was stirred at 80 °C for 1 d, cooled to RT, filtered and the solvent was removed under reduced pressure. The crude product was dissolved in DCM and precipitated in cold diethyl ether yielding **1a** as a pale-yellow solid (0.29 g, 86 %).

^{1}H NMR (600 MHz, D_2O): δ 9.51 (s, 24H, **A**), 8.96 (s, 24H, **B**), 8.46 (d, *J* = 53.1 Hz, 12H, **d**, **d'**), 7.57 (dd, *J* = 75.8, 44.2 Hz, 24H, **a**, **a'**, **b**, **b'**), 3.72 (s, 5209H, **g**, **h**), 3.40 (s, 18H, **i**), 2.64 (s, 18H, **f'**); **IR** (cm^{-1}): 2881.65 (m), 1465.90 (m), 1359.82 (w), 1340.53 (s), 1278.81 (m), 1240.23 (m), 1147.65 (m), 1097.50 (s), 1060.85 (m), 960.55 (m), 840.96 (m); **Mp.**: 60-65 °C.

Synthesis of cage 1b

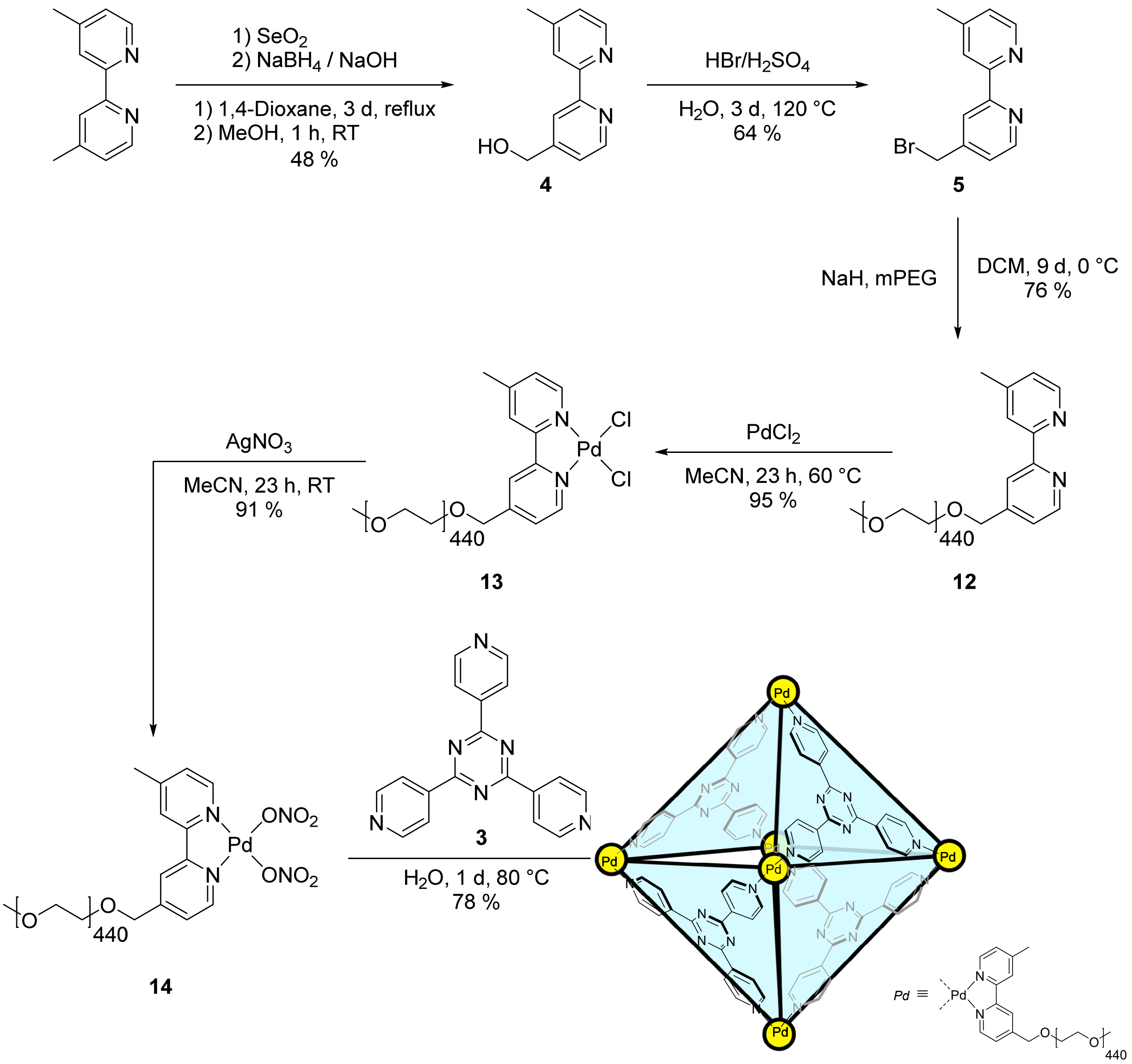

*Figure S 3: Synthetic overview of cage **1b**.*

Synthesis of 4-methoxypolyethyleneglycolmethyl-4'-methyl-2,2'-bipyridine (12)

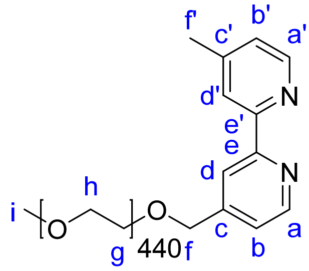

Poly(ethylene glycol) methyl ether (M_n = 20.000 Da, 5.0 g, 0.25 mmol, 1.0 eq.) was degassed with N_2 and dissolved in DCM (55 mL). The colourless solution was cooled to 0 °C. NaH (18 mg, 0.75 mmol, 3.0 eq.) was added and the reaction mixture was stirred for 1 h. 4-Bromomethyl-4'-methyl-2,2'-

bipyridine (**5**) (0.072 g, 0.28 mmol, 1.1 eq.) was added and the mixture was stirred for 4 d at 0 °C before another portion of NaH (18 mg, 0.75 mmol, 3.0 eq.) was added. After additional 5 d the reaction was quenched with MeOH (2 mL). The reaction was stirred for 30 min. and then filtered. The crude product was dialysed against deionized water for 4 d (MWCO: 1 kDa). After the solvent was removed, the colourless solid was dissolved in hot DCM and precipitated from cold diethyl ether. After filtration **12** was obtained as a colourless solid (3.8 g, 76 %).

^{1}H NMR (600 MHz, D_2O): δ 8.60 (d, *J* = 4.9 Hz, 1H, **a**), 8.49 (d, *J* = 4.8 Hz, 1H, **a’**), 8.28 (s, 1H, **d**), 8.19 (s, 1H, **d’**), 7.32 (dd, *J* = 4.9, 1.5 Hz, 1H, **b**), 7.10 (d, *J* = 5.0 Hz, 1H, **b’**), 4.63 (s, 2H, **f**), 3.60 (s, 1444H, **g**, **h**), 3.33 (s, 3H, **i**), 2.40 (s, 3H, **f’**).

Synthesis of (4-methoxypolyethyleneglycolmethyl-4’-methyl-2,2’-bipyridine)-dichloropalladium(II) (13)

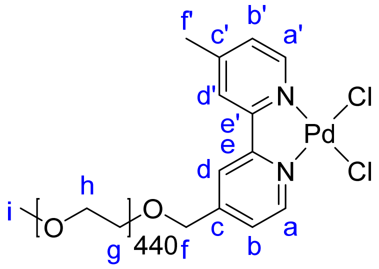

4-Methoxypolyethyleneglycolmethyl-4’-methyl-2,2’-bipyridine (**5**) (2.5 g, 0.12 mmol, 1.0 eq.) and $PdCl_2$ (22 mg, 0.12 mmol, 1.0 eq.) were suspended in MeCN (75 mL) and heated to 60 °C. After 23 h the yellow solution was cooled to RT, filtered and the solvent was removed under reduced pressure yielding **13** as a yellow solid (2.4 g, 95 %).

^{1}H NMR (600 MHz, D_2O): δ 9.06 (d, *J* = 5.9 Hz, 1H, **a**), 8.98 (d, *J* = 5.9 Hz, 1H, **a’**), 8.12 (s, 1H, **d**), 7.97 (s, 1H, **d’**), 7.40 (d, *J* = 6.0 Hz, 1H, **b**), 7.26 (s, 1H, **b’**), 4.75 (s, 2H, **f**), 3.58 (s, 1777H, **g**, **h**), 3.31 (s, 3H, **i**), 2.54 (s, 3H, **f’**).

Synthesis of (4-methoxypolyethyleneglycolmethyl-4'-methyl-2,2'-bipyridine)-dinitratopalladium(II) (14)

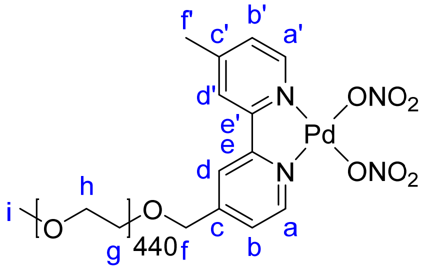

(4-methoxypolyethyleneglycolmethyl-4'-methyl-2,2'-bipyridine)-dichloro-palladium (**13**) (3.4 g, 0.17 mmol, 1.0 eq.) was dissolved in MeCN (55 mL) and $AgNO_3$ (0.1 g, 0.58 mmol, 3.4 eq.) was added, resulting in a colour change from yellow to a pale yellow. The conversion was tracked with 1 mL NMR-samples. After 23 h a full conversion of the starting material was observed and the AgCl was removed by centrifugation (4400 rpm, 90 min.). The resulting yellow supernatant was transferred into a round bottom flask and the solvent was removed by rotary evaporation at 30 °C in the dark to yield a yellow solid. This solid was dissolved in DCM, precipitated in cold diethyl ether, filtered, and dried to yield **14** as a yellow solid (3.1 g, 91 %).

^{1}H NMR (600 MHz, D_2O): δ 8.21 (s, 1H, **d**), 8.15 (d, *J* = 5.9 Hz, 1H, **a**), 8.07 (d, *J* = 5.9 Hz, 1H, **a'**), 8.06 (s, 1H, **d'**), 7.52 (d, *J* = 5.4 Hz, 1H, **b**), 7.37 (d, *J* = 6.1 Hz, 1H, **b'**), 4.76 (s, 2H, **f**), 3.57 (s, 1661H, **g**, **h**), 3.30 (s, 3H, **i**), 2.56 (s, 3H, **f'**).

*Synthesis of cage **1b***

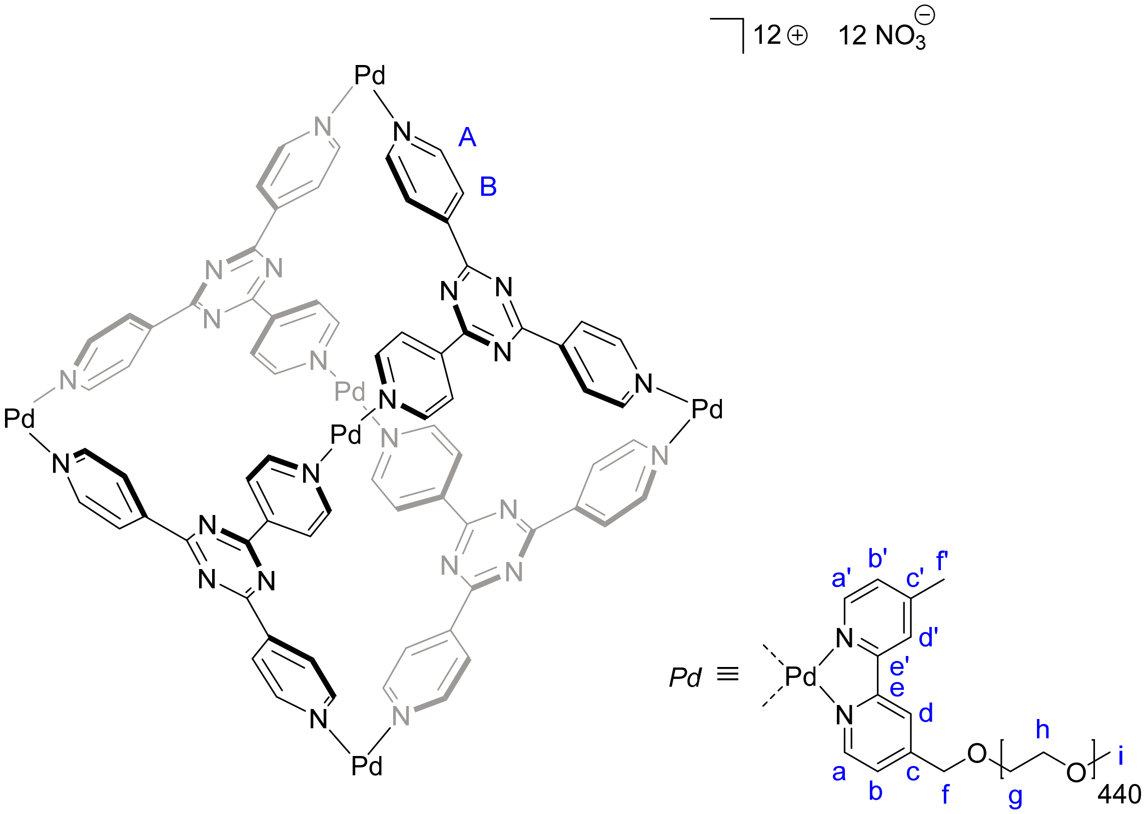

(4-methoxypolyethyleneglycolmethyl-4’-methyl-2,2’-bipyridine)-dinitrato palladium (**14**) (0.38 g, 0.019 mmol, 1.0 eq.) and 2,4,6-tris(4-pyridyl)-1,3,5-triazine (**3**) (3.9 mg, 0.013 mmol, 0.67 eq.) were suspended in H_2O (0.80 mL). The reaction was stirred at 80 °C for 1 d, cooled to RT, filtered and the solvent was removed under reduced pressure yielding **1b** as a pale-yellow solid (0.30 g, 78 %).

^{1}H NMR (600 MHz, D_2O): δ 9.52 (s, 24H, **A**), 8.96 (s, 24H, **B**), 8.46 (d, *J* = 56.0 Hz, 12H, **d**, **d’**), 7.78 – 7.45 (m, 24H, **a**, **a’**, **b**, **b’**), 3.72 (s, 10549H, **g**, **h**), 3.40 (s, 18H, **i**), 2.64 (s, 18H, **f’**).

Encapsulation experiments

Guest encapsulations with cage 1a

The following encapsulation experiments were done using cage **1a**.

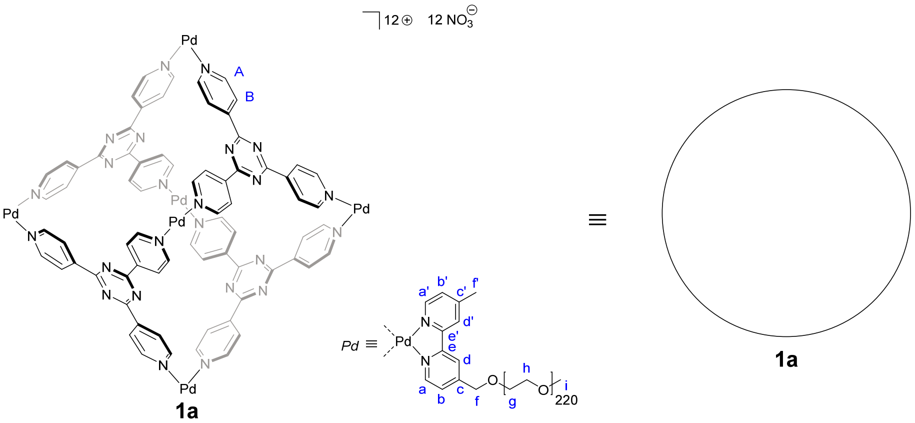

1a **1a**

For the following encapsulation experiments cage **1a** will be depicted as shown above plus its corresponding guest.

Cage 1a•(progesterone)

Cage **1a** (0.15 g, 0.0024 mmol, 1.0 eq.) was dissolved in H_2O (0.8 mL) and progesterone (7.4 mg, 0.024 mmol, 10 eq.) was added. The solution was stirred at 50 °C for 1 h. After removal of residual guests by filtration, the solvent was evaporated under reduced pressure to yield **1a•(progesterone)** as a yellow solid (0.13 g, 87 %).

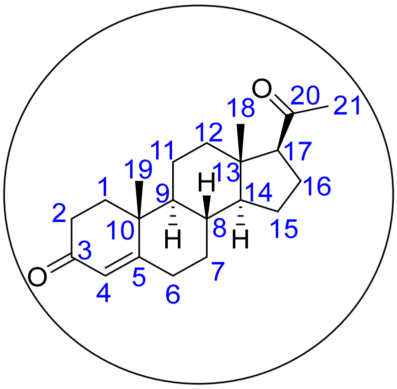

1a•(progesterone)

^{1}H NMR (600 MHz, D_2O): δ 9.61 (s, 24H, **A**), 9.00 (s, 24H, **B**), 8.46 (d, *J* = 57.2 Hz, 12H, **d**, **d'**), 7.61 (t, *J* = 77.4 Hz, 24H, **a**, **a'**, **b**, **b'**), 3.72 (s, 5016H, **g**, **h**), 3.40 (s, 18H, **i**), 2.64 (s, 18H, **f'**), 2.18 (s, 4H, **21**), 2.05 (d, *J* = 7.1 Hz, 1H, **17**), 1.17 (s, 1H, could not be assigned due to overlaps in the spectrum), 0.67 (s, 3H, **6 or 7**), 0.44 (s, 3H, **12**, last proton could not be assigned due to overlaps), -0.66 (s, 8H, **9**, **11**, **15**, **19**), -1.15

(s, 4H, **18**); **IR** (cm^{-1}): 2879.72 (m), 1660.71 (w), 1465.90 (m), 1359.82 (w), 1340.53 (s), 1278.81 (m), 1240.23 (m), 1143.79 (m), 1097.50 (s), 1058.92 (m), 960.55 (m), 840.96 (m); **Mp.**: 60-65 °C.

Cage 1a•(ibuprofen)$_2$

Cage **1a** (0.15 g, 0.0024 mmol, 1.0 eq.) was dissolved in H_2O (0.8 mL) and ibuprofen (4.9 mg, 0.024 mmol, 10 eq.) was added. The solution was stirred at 50 °C for 1 h. After removal of residual guests by filtration, the solvent was evaporated under reduced pressure to **1a•(ibuprofen)**$_2$ as a yellow solid (0.14 g, 93 %).

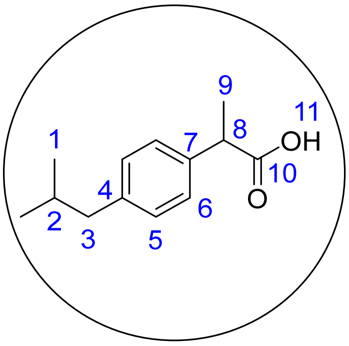

1a•(ibuprofen)$_2$

^{1}H NMR (600 MHz, D_2O): δ 9.64 (s, 24H, **A**), 9.03 (s, 24H, **B**), 8.49 (d, J = 53.8 Hz, 12H, **d**, **d'**), 7.62 (t, J = 74.8 Hz, 24H, **a**, **a'**, **b**, **b'**), 5.69 (s, 4H, **6**), 3.75 (s, 5889H, **g**, **h**), 3.43 (s, 18H, **i**), 3.02 (s, 2H, **8**), 2.67 (s, 18H, **f'**), 1.32 (s, 4H, **3**), 0.72 (s, 6H, **9**), 0.36 (s, 2H, **2**), -0.41 (s, 12H, **1**); **IR** (cm^{-1}): 2881.65 (m), 2360.87 (w), 1521.84 (w), 1465.90 (m), 1359.82 (w), 1342.46 (s), 1278.81 (m), 1242.16 (m), 1143.79 (m), 1097.50 (s), 1058.92 (m), 962.48 (m), 840.96 (m); **Mp.**: 60-65 °C.

Cage 1a•(phenolphthalein)

Cage **1a** (0.25 g, 0.0039 mmol, 1.0 eq.) was dissolved in H_2O (1.2 mL) and phenolphthalein (0.40 mg, 0.0020 mmol, 0.50 eq.) was added. The solution was stirred at 50 °C for 2 h. After removal of residual guests by filtration, the solvent was evaporated under reduced pressure to yield **1a•(phenolphthalein)** as a yellow solid (0.24 g, 96 %).

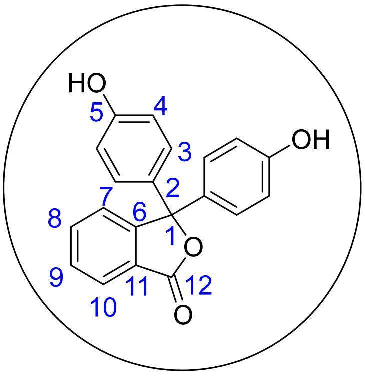

1a•(phenolphthalein)

^{1}H NMR (600 MHz, D_2O): 9.45 (s, 24H, **A**), 8.83 (s, 24H, **B**), 8.37 (d, *J* = 52.9 Hz, 12H, **d**, **d'**), 7.62 – 7.33 (m, 24H, **a**, **a'**, **b**, **b'**), 3.63 (s, 5424H, **g, h**), 3.31 (s, 18H, **i**), 2.55 (s, 18H, **f'**); **IR** (cm^{-1}): 2883.58 (m), 1465.90 (m), 1359.82 (w), 1340.53 (s), 1278.81 (m), 1240.23 (m), 1147.65 (m), 1097.50 (s), 1060.85 (m), 960.55 (m), 840.96 (m); **Mp.**: 60-65 °C.

Guest encapsulations with cage 2

For each of the following encapsulation experiments 1 mL of a 5 mM cage solution (in D_2O) of cage **2** was added to 5 eq. of the respective guest molecule. The resulting mixture was stirred at 80 °C for 2 h. After removal of residual guests by filtration, ^{1}H NMR spectra of the solution were measured. The yields of the inclusion complexes were determined by comparison of the integral ratio between host and guest in the ^{1}H NMR spectra.

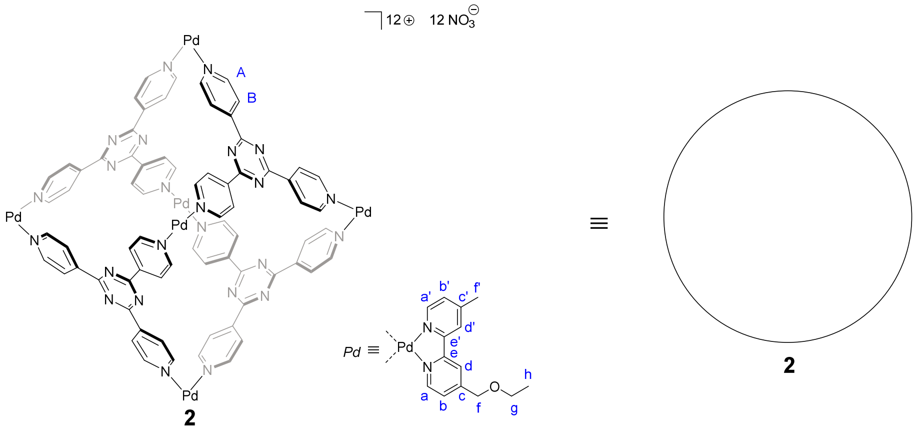

For the following encapsulation experiments cage **2** will be depicted as shown above plus its corresponding guest.

Cage 2•(progesterone)

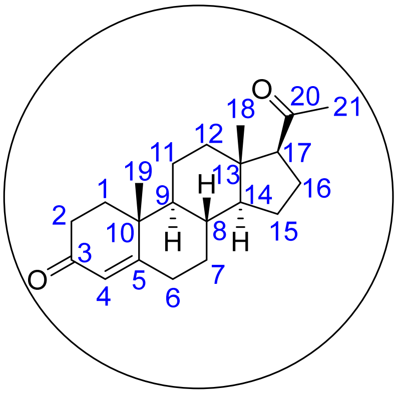

2•(progesterone)

^{1}**H NMR** (600 MHz, D_2O): δ 9.57 (dd, *J* = 12.3, 6.0 Hz, 24H, **A**), 8.97 (dt, *J* = 6.6, 3.2 Hz, 24H, **B**), 8.43 (s, 6H, **d**), 8.36 (s, 6H, **d´**), 7.72 (d, *J* = 5.9 Hz, 6H, **a**), 7.58 (dd, *J* = 8.0, 6.0 Hz, 12H, **b, a´**), 7.48 – 7.44 (m, 6H, **b´**), 4.86 (s, 12H, **f**), 3.76 (q, *J* = 7.0 Hz, 12H, **g**), 2.99 (s, 1H, **4**), 2.63 (s, 18H, **f'**), 2.24 – 2.13 (m, 4H, **21**), 2.00 (d, *J* = 0.7 Hz, 1H, **17**), 1.67 (s, 1H, cannot be assigned due to overlaps in the spectrum), 1.30 (t, *J* = 7.0 Hz, 19H, **h**, cannot be assigned unambiguously), 0.94 (s, 1H, cannot be assigned unambiguously), 0.66 (s, 2H, **6 or 7**), 0.57 (s, 1H, cannot be assigned unambiguously), 0.34 (s, 2H, **16**), 0.20 (d, *J* = 11.7 Hz, 1H, cannot be assigned unambiguously), -0.20 (s, 1H, **8 or 14**), -0.48 (s, 1H, **12**), -0.62 – -0.87 (m, 8H, **9**, **11**, **15**, **19**), -1.22 (s, 4H, **18**), -1.48 (s, 1H, **8 or 14**); ^{13}**C{**^{1}**H} NMR** (151 MHz, D_2O): δ 213.44 (**20**), 201.03 (**3**), 173.74 (**5**), 169.81 (**D**), 156.98 (**e´**), 156.59 (**c´**), 156.02 (**e**), 155.57 (**c**), 152.82 (**A**), 150.18 (**a**), 149.55 (**a´**), 145.78 (**C**), 128.92 (**b**), 126.55 (**B**), 125.69 (**b´**), 125.30 (**d**), 121.99 (**d´**), 121.43 (**4**), 69.86 (**f**), 67.45 (**g**), 62.77 (**17**), 55.59 (**14**), 53.80 (**9**), 42.39 (**13**), 37.58 (**12**), 37.35 (**10**), 34.80 (**1 or 8**), 32.87 (**1 or 8**), 32.80 (**2**), 31.25 (**6**, **7**), 31.15 (**21**), 22.33 (**15 or 16**), 21.91 (**15 or 16**), 21.14 (**f´**), 19.05 (**11**), 14.66 (**19**), 14.40 (**h**), 11.00 (**18**); **DOSY** (D_2O, 298 K): D = $2.01\cdot10^{-10}$ m^2/s; **IR** (cm^{-1}): 2358.94 (w), 1616.35 (w), 1506.41 (m), 1373.32 (m), 1338.60 (m), 1267.23 (w), 1114.86 (w), 962.48 (b), 810.10 (m); **Mp.**: 296.8-298.4 °C (dec.).

Cage 2•(ibuprofen)$_2$

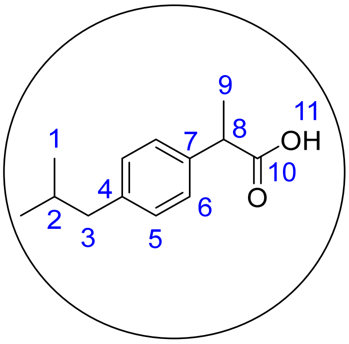

2•(ibuprofen)$_2$

^{1}**H NMR** (600 MHz, D_2O): δ 9.60 (d, *J* = 6.1 Hz, 24H, **A**), 9.00 – 8.95 (m, 24H, **B**), 8.43 (s, 6H, **d**), 8.36 (s, 6H, **d'**), 7.70 (d, *J* = 6.0 Hz, 6H, **a**), 7.59 – 7.54 (m, 12H, **b, a'**), 7.45 (d, 6H, **b'**), 5.25 (s, 4H, **6**), 4.86 (s, 12H, **f**), 4.52 (s, 4H, **5**), 3.75 (q, *J* = 7.1 Hz, 12H, **g**), 2.88 – 2.83 (m, 2H, **8**), 2.63 (s, 18H, **f'**), 1.30 (t, *J* = 7.0 Hz, 18H, **h**), 1.23 – 1.10 (m, 4H, **3**), 0.43 (d, *J* = 6.9 Hz, 6H, **9**), 0.18 (s, 2H, **2**), -0.61 (s, 12H, **1**); ^{13}**C{**^{1}**H} NMR** (151 MHz, D_2O): δ 177.04 (**10**), 169.53 (**D**), 156.98 (**e´**), 156.65 (**c´**), 156.02 (**e**), 155.62 (**c**), 152.89 (**A**), 150.17 (**a**), 149.54 (**a´**), 145.56 (**C**), 138.32 (**4**), 137.23 (**7**), 128.94 (**b´**), 127.17 (**5**), 126.44 (**B**), 125.71 (**b**), 125.34 (**d´**), 125.10 (**6**), 122.03 (**d**), 69.86 (**f**), 67.47 (**g**), 44.48 (**8**), 43.61 (**3**), 28.89 (**2**), 21.15 (**f´**), 20.59 (**1**), 18.14 (**9**), 14.41 (**h**); **DOSY** (D_2O, 298 K): D = $2.13 \cdot 10^{-10}$ m^2/s; **IR** (cm^{-1}): 2985.81 (m), 2970.38 (m), 2885.51 (m), 2355.08 (w), 1375.25 (w), 1228.66 (w), 1220.94 (w), 1074.35 (s), 1056.99 (s), 864.11 (w); **Mp.**: 300.1-302.4 °C (dec.)

Cage 2•(phenolphthalein)

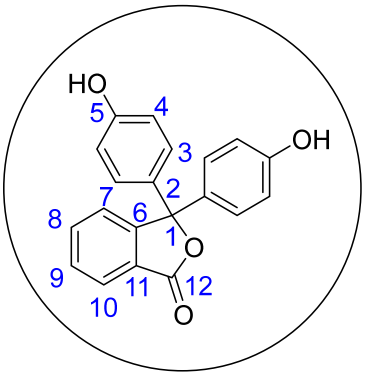

2•(phenolphthalein)

^{1}**H NMR** (600 MHz, D_2O): δ 9.45 – 9.41 (m, 24H, **A**), 8.82 – 8.78 (m, 24H, **B**), 8.35 – 8.32 (m, 6H, **d**), 8.29 – 8.26 (m, 6H, **d´**), 7.58 (d, *J* = 6.0 Hz, 6H, **a**), 7.50 – 7.47 (m, 6H, **b**), 7.45 (d, *J* = 6.0 Hz, 6H, **a´**), 7.38 – 7.33 (m, 6H, **b´**), 6.88 (s, 1H, **10**), 6.50 (s, 1H, **9**), 6.34 (s, 1H, **8**), 5.62 (s, 1H, **7**), 5.22 (s, 4H, **4**), 5.12 (s, 4H, **3**), 4.77 (s, 12H, **f**), 3.66 (q, *J* = 7.0 Hz, 12H, **g**), 2.54 (s, 18H, **f´**), 1.21 (t, *J* = 7.0 Hz, 18H, **h**); **DOSY** (D_2O, 298 K): D = $1.88 \cdot 10^{-10}$ m^2/s.

Cage 2•(testosterone)

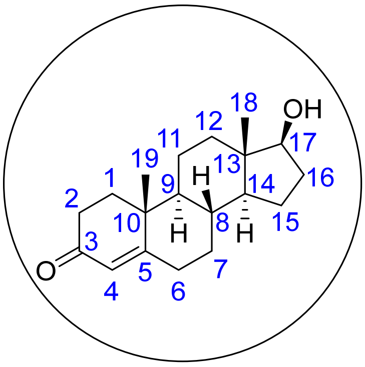

2•(testosterone)

The assignment was done based on the work of Kamo *et al*.[4]

^{1}H NMR (600 MHz, D_2O): δ 9.61 – 9.53 m, 24H, **A**), 8.99 – 8.95 (m, 24H, **B**), 8.43 (s, 6H, **d**), 8.37 (s, 6H, **d´**), 7.71 (d, *J* = 5.9 Hz, 6H, **a**), 7.60 – 7.56 (m, 6H, **b, a´**), 7.46 (d, *J* = 6.2 Hz, 6H, **b´**), 4.86 (s, 12H, **f**), 3.76 (q, *J* = 7.0 Hz, 12H, **g**), 3.16 – 3.11 (m, 1H, **4**), 2.63 (s, 18H, **f´**), 1.93 (s, 1H, **17**), 1.51 – 1.48 (m, 2H, **2**), 1.30 (t, *J* = 7.0 Hz, 18H, **h**), 0.87 (d, *J* = 44.8 Hz, 4H, **6**, **16**), 0.71 (s, 1H, cannot be assigned unambiguously), 0.51 (d, 2H, **7**), 0.23 (s, 1H, cannot be assigned unambiguously), -0.04 (s, 1H, cannot be assigned unambiguously), -0.30 – -0.84 (m, 13H, **18**, **19** cannot be assigned unambiguously), -1.04 – -1.25 (m, 2H, **9**, **14**); **^{13}C{^{1}H} NMR** (151 MHz, D_2O): δ 200.81 (**3**), 174.12 (**5**), 169.82 (**D**), 156.98 (**e´**), 156.60 (**c´**), 156.02 (**e**), 155.57 (**c**), 152.82 (d, *J* = 6.9 Hz, **A**), 150.18 (**a**), 149.55 (**a´**), 145.77 (**C**), 128.92 (**b´**), 126.57 (d, *J* = 8.3 Hz, **B**), 125.69 (**b**), 125.31 (**d´**), 122.00 (**d**), 121.31 (**4**), 80.06 (**17**), 69.87 (**f**), 67.45 (**g**), 54.16 (**9**), 50.08 (**14**), 41.43 (**13**), 37.48 (**10**), 35.43 (**12**), 34.75 (**1**), 33.07 (**6**), 32.70 (**2**), 31.13 (**8**), 29.25 (**16**), 21.28 (**15**), 21.14 (**f´**), 19.01 (**11**), 14.84 (**19**), 14.41 (**h**), 9.19 (**18**); **DOSY** (D_2O, 298 K): D = $2.06\cdot10^{-10}$ m^2/s; **IR** (cm^{-1}): 2360.87 (m), 2320.37 (w), 1618.28 (w), 1485.63 (s), 1330.88 (s), 1313.52 (s), 1265.30 (m), 1105.21 (w), 1062.78 (w), 968.27 (b), 808.17 (s); **Mp.**: 298.4-301.6 °C (dec.).

Cage 2•(drospirenone)

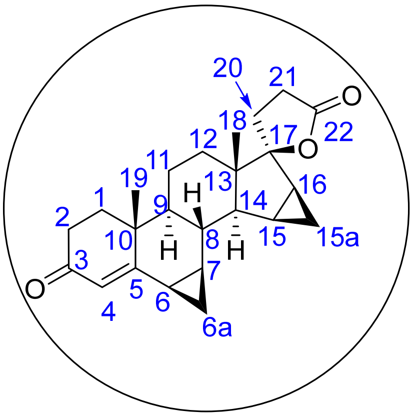

2•(drospirenone)

The assignment was done based on the work of Baldessari *et al.*[5]

^{1}H NMR (600 MHz, D_2O): δ 9.58 (dd, *J* = 21.8, 6.1 Hz, 24H, **A**), 8.99 (dd, *J* = 34.7, 6.0 Hz, 24H, **B**), 8.59 – 8.39 (m, 6H, **d**), 8.36 (d, *J* = 1.7 Hz, 6H, **d´**), 7.69 (d, *J* = 6.0 Hz, 6H, **a**), 7.57 (m, 12H, **b**, **a´**), 7.48 (m, 6H, **b´**), 5.21 (s, 1H, **4**), 4.86 (s, 12H, **f**), 3.76 (q, *J* = 7.1 Hz, 12H, **g**), 2.63 (s, 18H, **f´**), 2.31 (s, 1H, **21**), 1.82 – 1.57 (m, 3H, **2**, **21**), 1.30 (t, *J* = 7.0 Hz, 18H, **h**), 0.87 (d, *J* = 43.6 Hz, 2H, **20**), -0.02 (d, *J* = 59.6 Hz, 2H, cannot be assigned unambiguously), -0.21 (s, 1H, cannot be assigned unambiguously), -0.30 – -0.71 (m, 4H, cannot be assigned unambiguously), -0.83 (d, *J* = 46.7 Hz, 3H, cannot be assigned unambiguously), -0.98 (s, 3H, **19**), -1.12 (s, 5H, **18**), -1.31 (s, 1H, **6a**), -1.60 (s, 1H, **15a**); **^{13}C{^{1}H} NMR** (151 MHz, D_2O): δ 200.91 (**3**), 179.31 (**22**), 174.23 (**5**), 169.92 (**D**), 157.00 (**e´**), 156.63 (**c´**), 156.05 (**e**), 155.61 (**c**), 152.90 (d, *J* = 18.0 Hz, **A**), 150.18 (**a**), 149.55 (**a´**), 145.79 (**C**), 128.95 (**b´**), 126.62 (d, *J* = 42.7 Hz, **B**), 125.72 (**b**), 125.34 (**d´**), 124.42 (**5**), 122.03 (**d**), 95.74 (**17**), 69.89 (**f**), 67.48 (**g**), 51.19 (**14 or 9**), 50.76 (**9 or 24**), 40.40 (**13**), 36.61 (**10**), 36.10 (**12**), 35.59 (**11**), 33.26 (**8**), 31.62 (**20**), 30.54 (**1 or 2**), 29.47 (**2 or 1**), 23.81 (**16**), 21.17 (**f´**), 19.77 (**18 or 7**), 19.50 (**7 or 18**), 18.31 (**6**), 17.04 (**6a**), 16.25 (**19**), 14.84 (**15**), 14.44 (**h**), 8.57 (**15a**); **DOSY** (D_2O, 298 K): D = $2.13 \cdot 10^{-10}$ m^2/s; **IR** (cm^{-1}): 2966.52 (w), 2937.59 (w), 2357.01 (w), 1604.77 (s), 1388.75 (s), 1323 (s), 1288.45 (m), 1114.86 (m), 1074.35 (m), 1047.35 (m), 1001.06 (w), 989.48 (w), 920.05 (m), 767.67 (w), 702.09 (w), 626.87 (s); **Mp.**: 298.5-300.3°C (dec.).

Sonication experiments

All sonication experiments were performed with a Vibra cell VCX 500 sonicator with a frequency of 20 kHz, an amplitude of 30 % and a full wave probe (13 mm). For each sonication experiment a 5 $mg \cdot mL^{-1}$ cage solution (in H_2O) was degassed with N_2 in a suslick vessel and cooled with an ice-water bath. A sequence of 1 s on and 1 s off was chosen, if not stated otherwise. Samples were taken out by a degassed syringe. The solution was exposed to N_2 during the whole sonication.

Cage 1a

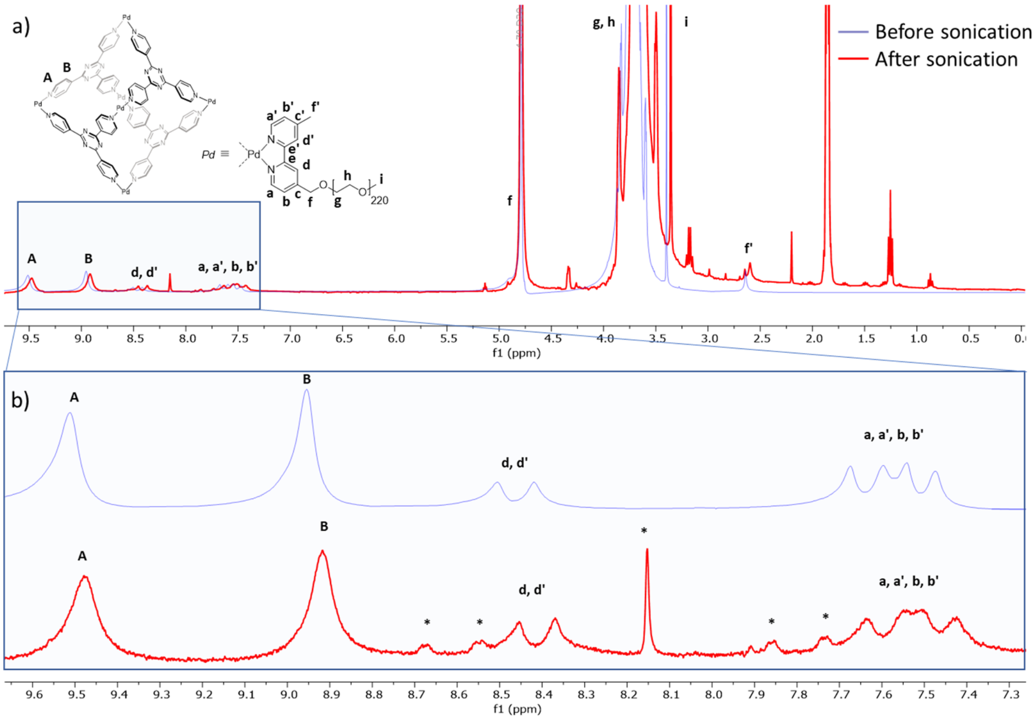

*Figure S 4: ^{1}H NMR (D_2O) of cage **1a** before (blue) and after sonication (red) with a) the complete spectral range and b) the enlarged region for characteristic signals of the supramolecular host. Sonicated for 1 h (1 s on, 2 s off). The ^{1}H NMR after the sonication was baseline corrected using a Whittaker Smoother: filter=45, smooth factor=16384, the unedited spectrum is additionally provided in the "Spectra encapsulation and release studies" section. The asterisks indicate fragmented cage, an accurate assignment was not always possible.*

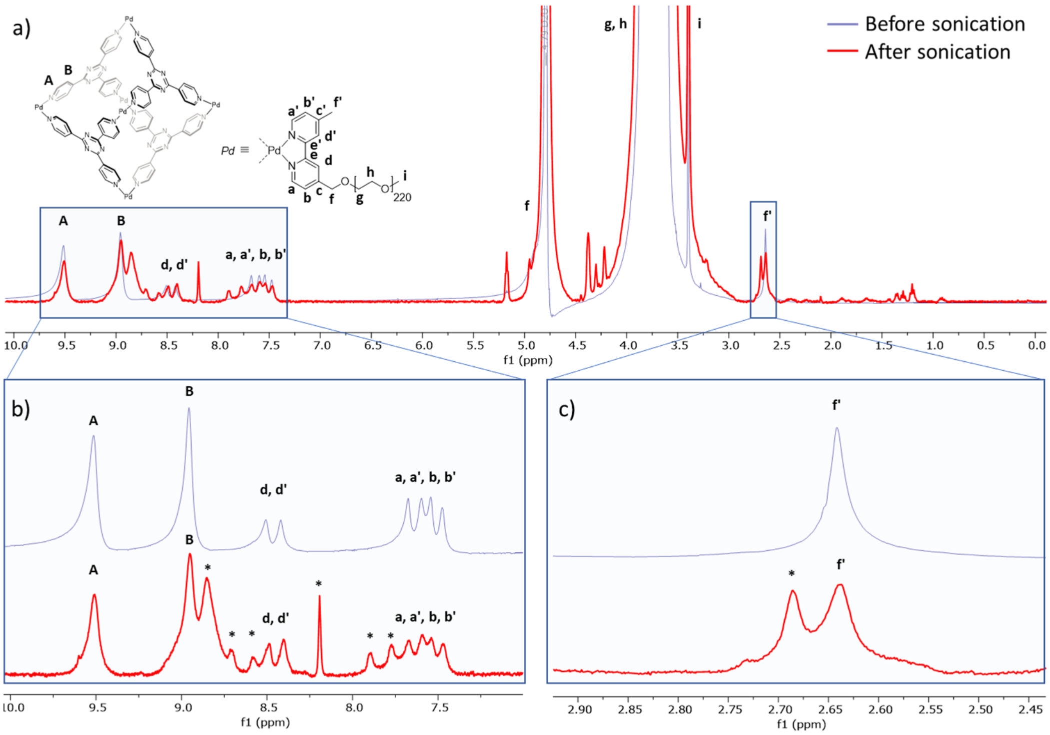

*Figure S 5: ^{1}H NMR (D_2O) of cage **1a** before (blue) and after sonication (red) with a) the complete spectral range and b+c) the enlarged region for characteristic signals of the supramolecular host. Sonicated for 3 h (1 s on, 1 s off). The ^{1}H NMR after the sonication was baseline corrected using a Whittaker Smoother: filter=45, smooth factor=16384, the unedited spectrum is additionally provided in the "Spectra encapsulation and release studies" section. The asterisks indicate the fragmented cage, an accurate assignment of the resulting signals was not always possible.*

Cage 1a•(progesterone)

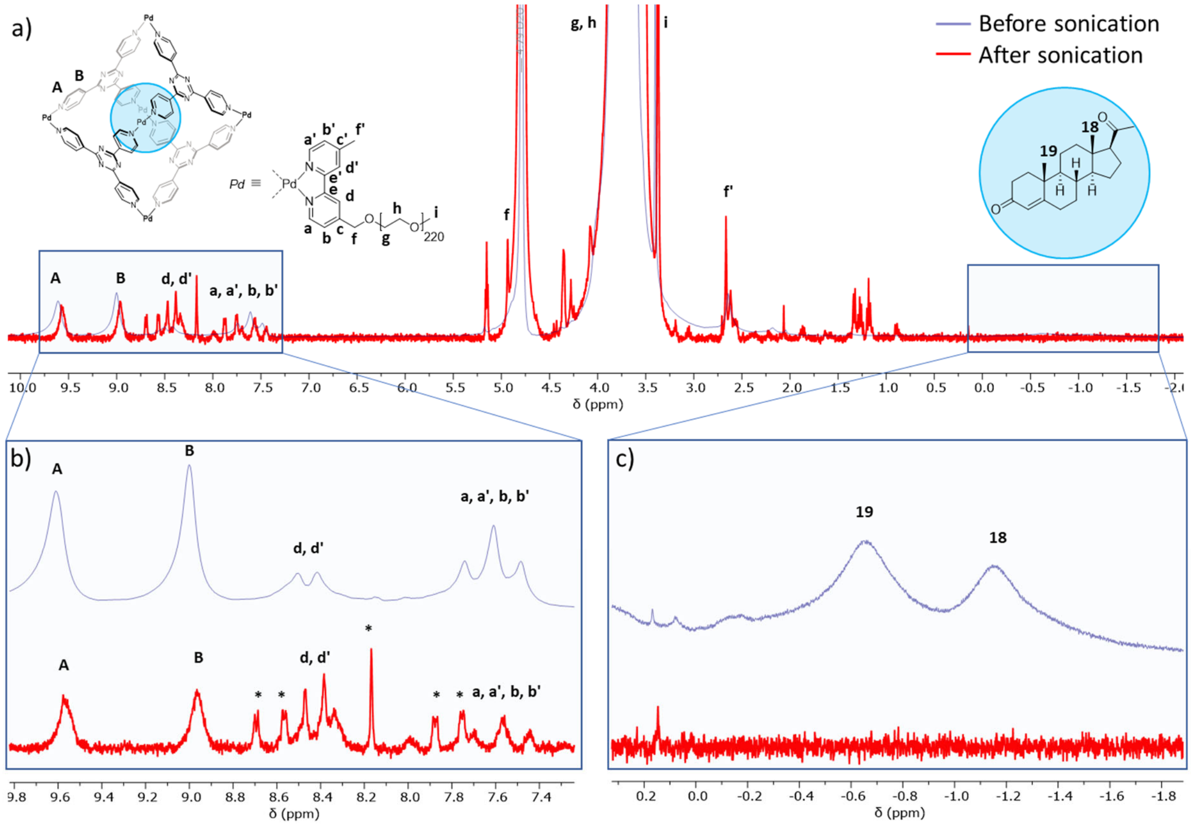

*Figure S 6: ¹H NMR (D_2O) of cage **1a•(progesterone)** before (blue) and after sonication (red) with a) the complete spectral range, b) the enlarged region for characteristic signals of the supramolecular host and c) the region showing guest encapsulation and release. Sonicated for 3 h (1 s on, 1 s off). The ¹H NMR after the sonication was baseline corrected using a Whittaker Smoother: filter=37, smooth factor=16384, the unedited spectrum is additionally provided in the "Spectra encapsulation and release studies" section again. The asterisks indicate the fragmented cage, an accurate assignment of the resulting signals was not always possible; c) the release of progesterone can be observed.*

Cage 1a•(ibuprofen)$_2$

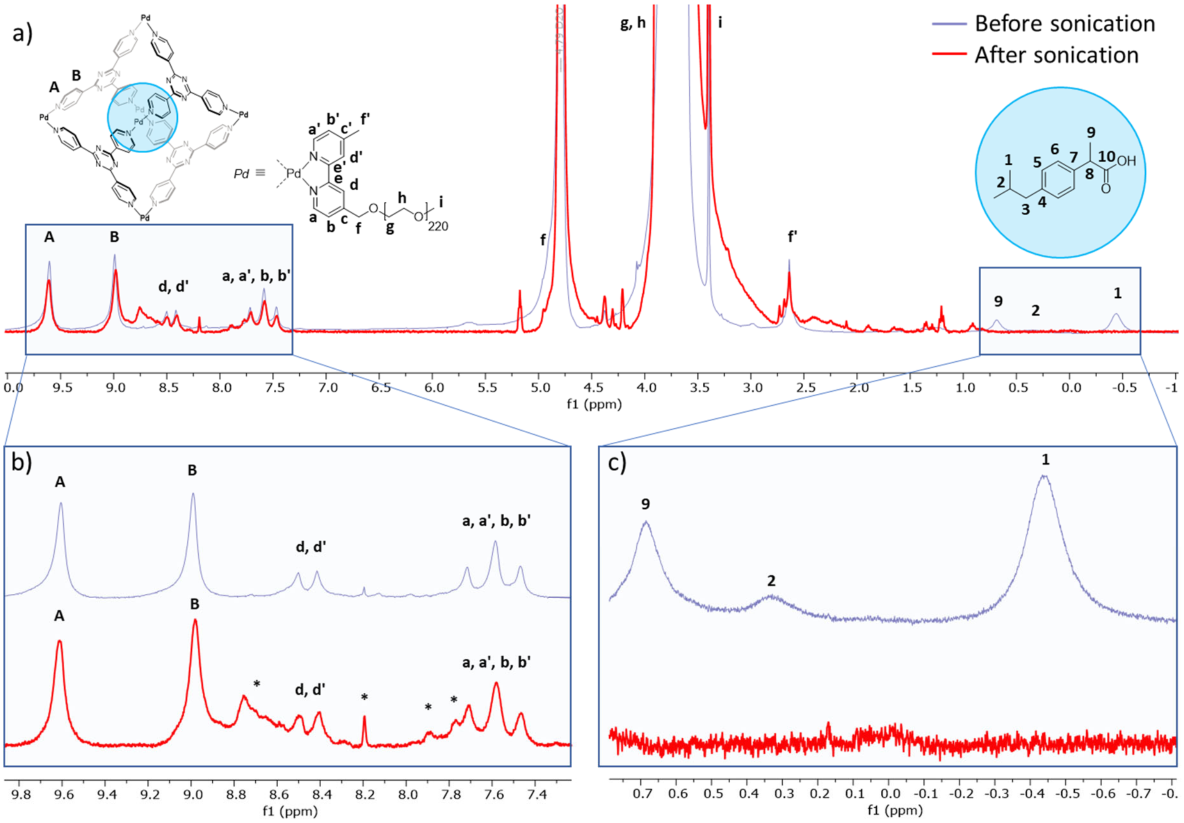

*Figure S 7: ^{1}H NMR (D_2O) of cage **1a•(ibuprofen)**$_2$ before (blue) and after sonication (red) with a) the complete spectral range, b) the enlarged region for characteristic signals of the supramolecular host and c) the region showing guest encapsulation and release. Sonicated for 3 h. (1 s on, 1 s off). The ^{1}H NMR after the sonication was baseline corrected using a Whittaker Smoother: filter=62, smooth factor=16384, the unedited spectrum is additionally provided in the "Spectra encapsulation and release studies" section again. The asterisks indicate the fragmented cage, where an accurate assignment of the resulting signals was not always possible. For c), the release of ibuprofen can be observed.*

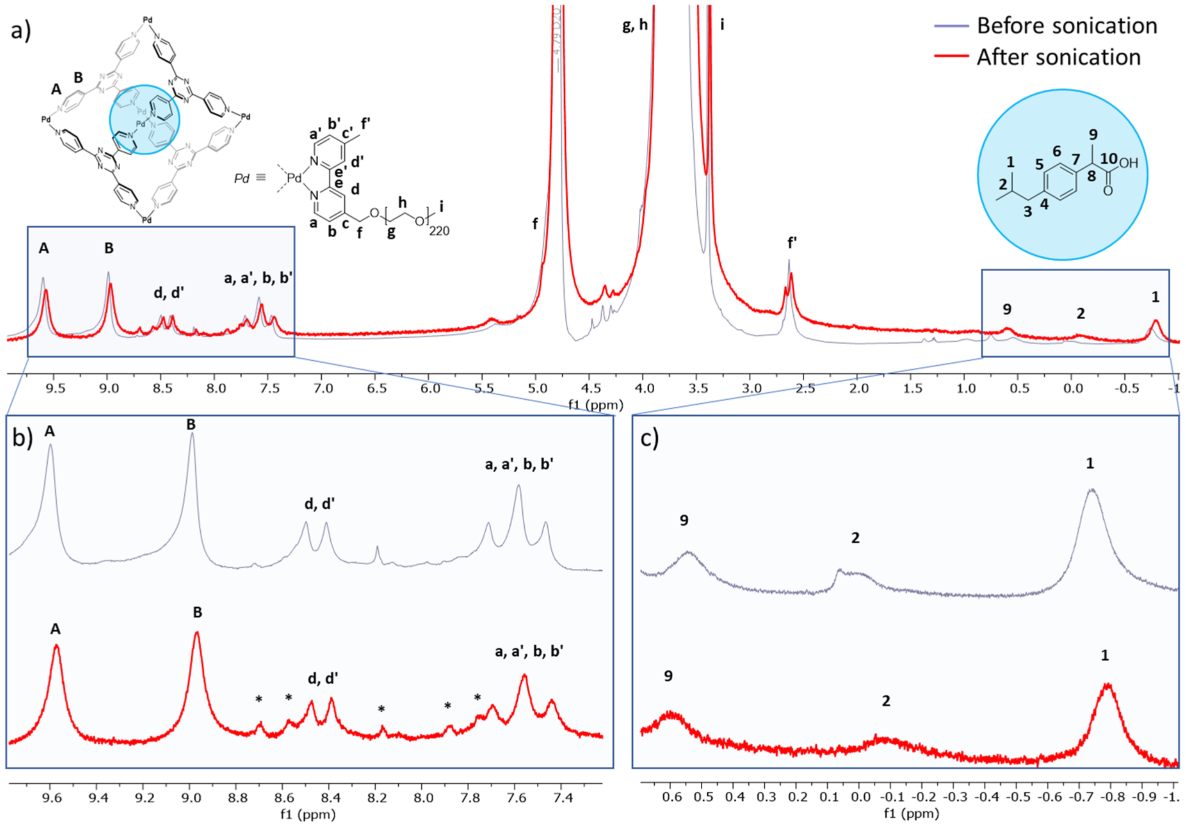

*Figure S 8: ^{1}H NMR (D_2O) of cage **1a•**(i**buprofen**)$_2$ before (blue) and after sonication (red) with a) the complete spectral range, b) the enlarged region for characteristic signals of the supramolecular host and c) the region showing guest encapsulation. Sonicated for 15 min. (1 s on, 2 s off). The asterisks indicate the fragmented cage. An accurate assignment of the resulting signals was not always possible. b) Some fragmentation of cage **1a** can be observed, c) indicates that no guest was released.*

Cage 1a•(phenolphthalein)

Cage **1a•**(**phenolphthalein**) (0.20 g, 3.1 µmol) was dissolved in a 1 mM carbonate buffer (10 mL) in a suslick vessel, degassed with N_2 and cooled with an ice-water bath. The solution was sonicated for a total of 6 h (1 sec on, 1 sec off). Every 30 min a sample was taken with a degassed syringe and diluted to a 20 µM solution with carbonate buffer. For the UV-Vis spectra a 1-cm quartz cell (3.0 mL) was used. The absorbance for phenolphthalein at 529 nm was monitored. An increase is observable at 529 nm but at the same time the absorbance for the whole UV-Vis spectra increases. This might be due to the high concentration of the carbonate buffer during the sonication resulting in an unwanted decomposition or side reaction of cage **1a**.

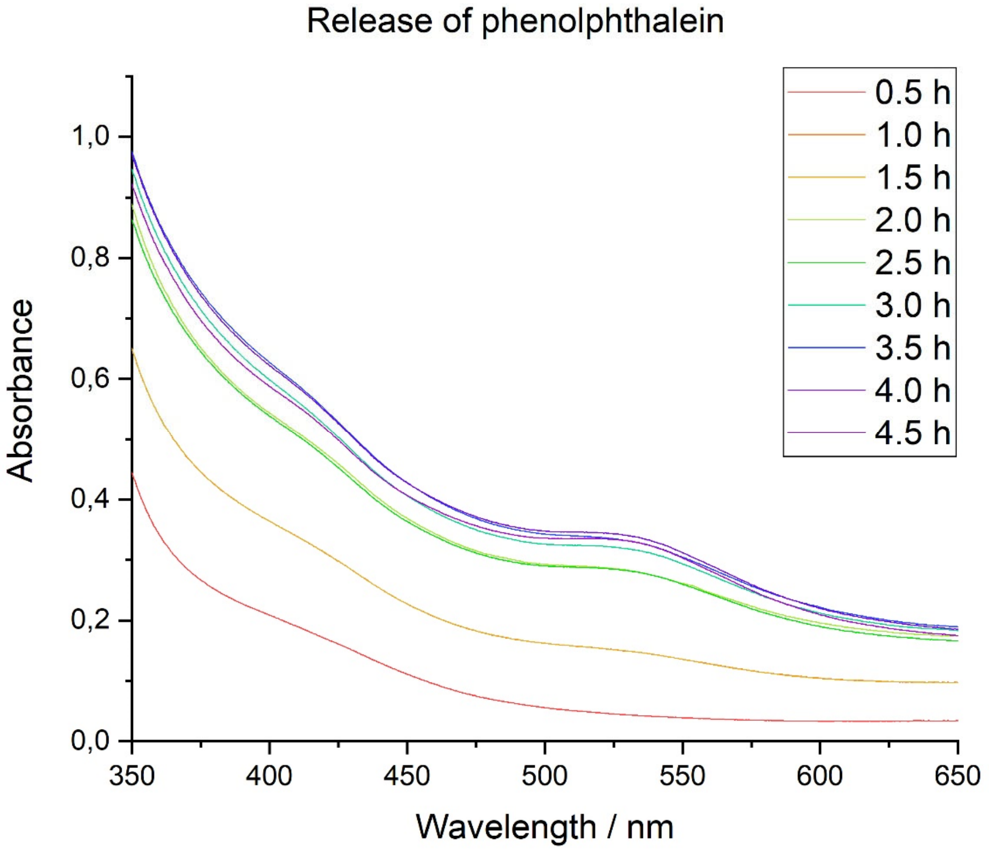

*Figure S 9: UV-Vis spectra of the sonication of cage **1a**•(**phenolphthalein**) (20 µM) in carbonate buffer (1 mM).*

Cage 1b

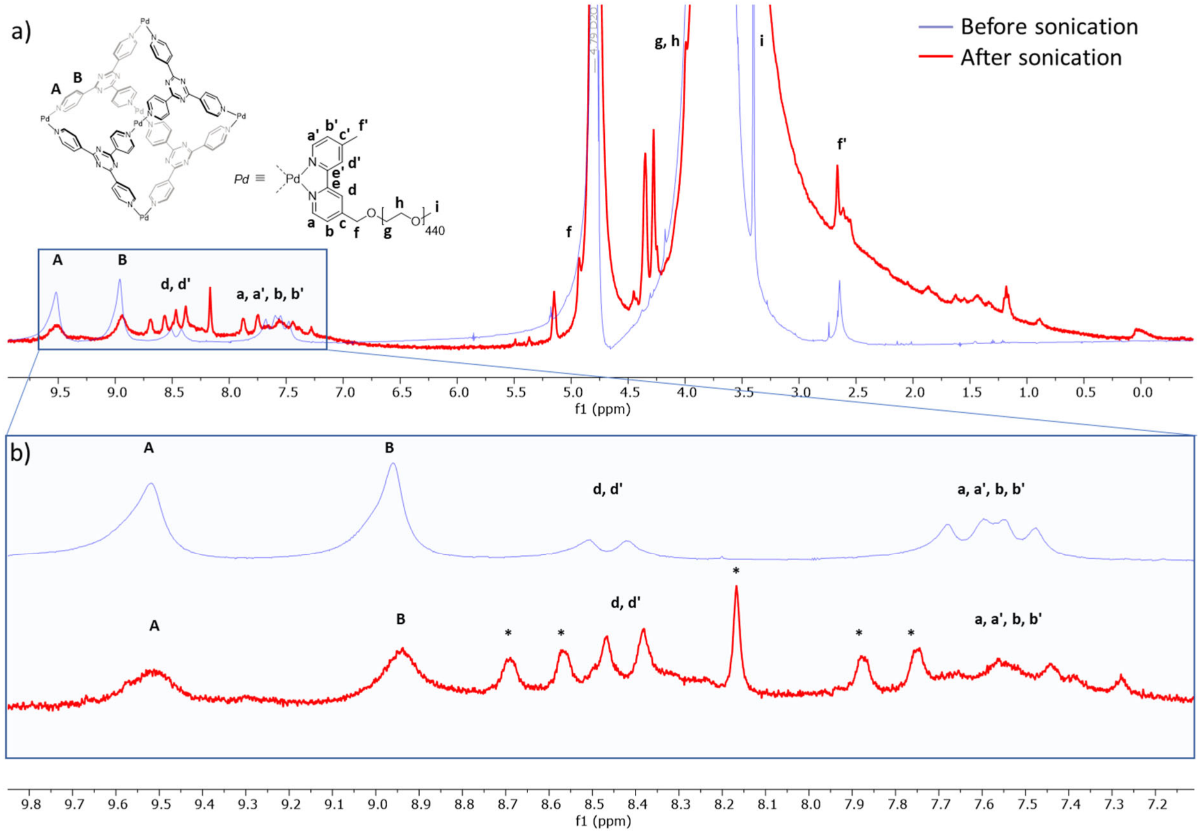

*Figure S 10: ^{1}H NMR (D_2O) of cage **1b** before (blue) and after sonication (red) with a) the complete spectral range and b) the enlarged region for characteristic signals of the supramolecular host. Sonicated for 1 h. (1 s on, 1 s off). For the ^{1}H NMR after the sonication a baseline correction with a polynomial fit: filter=67, polynomial order=5 was employed. The unedited spectrum is additionally provided in the "NMR encapsulation and release studies" section. The asterisks indicate fragmented cage **1b**. An accurate assignment of the resulting signals was not possible for all signals.*

Cage 2

For the sonication experiments with cage **2** a 1 mg·mL^{-1} cage solution in H_2O was used instead of the 5 mg/ml. This solution was degassed with N_2 in a suslick vessel and cooled with an ice-water bath. A sequence of 1 sec on and 1 sec off was chosen. Samples were taken out by a degassed syringe. The solution was exposed to N_2 during the whole sonication.

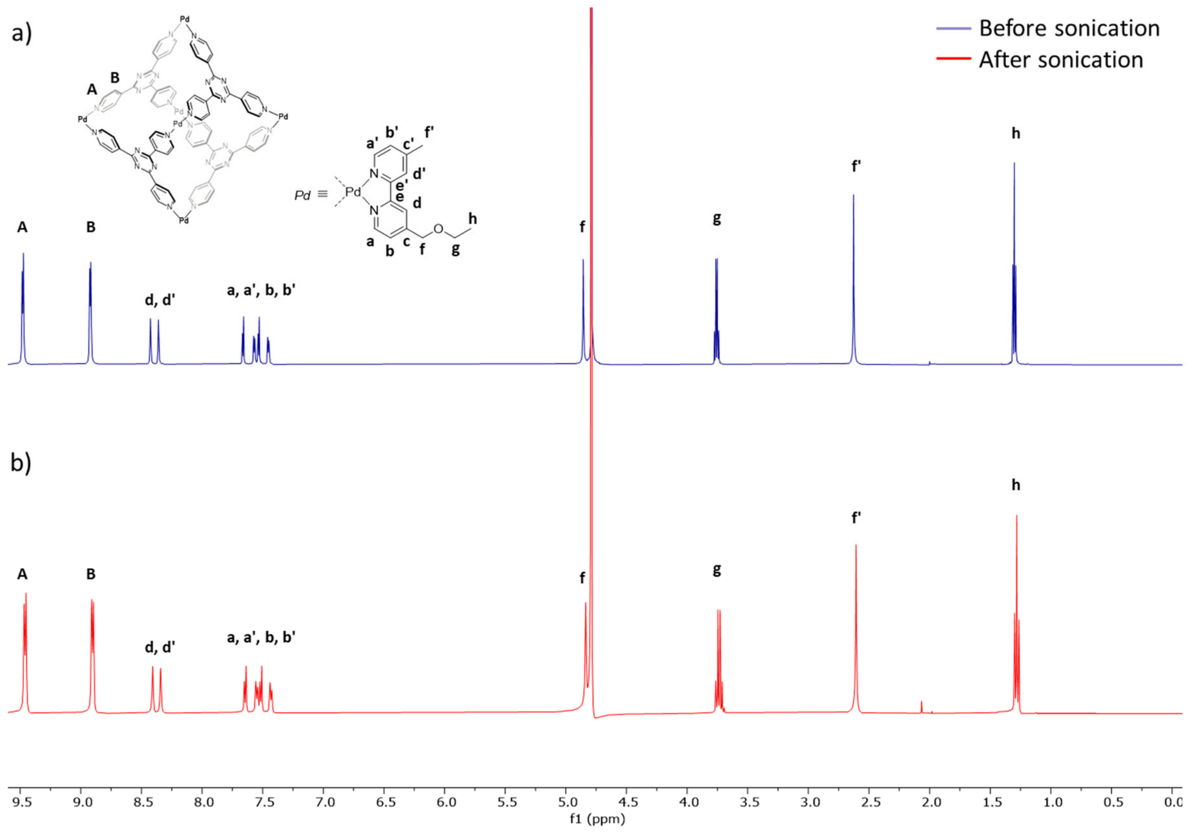

*Figure S 11: a) ^{1}H NMR (D_2O) of cage **2** before (blue) and b) after sonication (red). Sonicated for 3 h (1 s on, 1 s off). No fragmentation of cage **2** can be observed.*

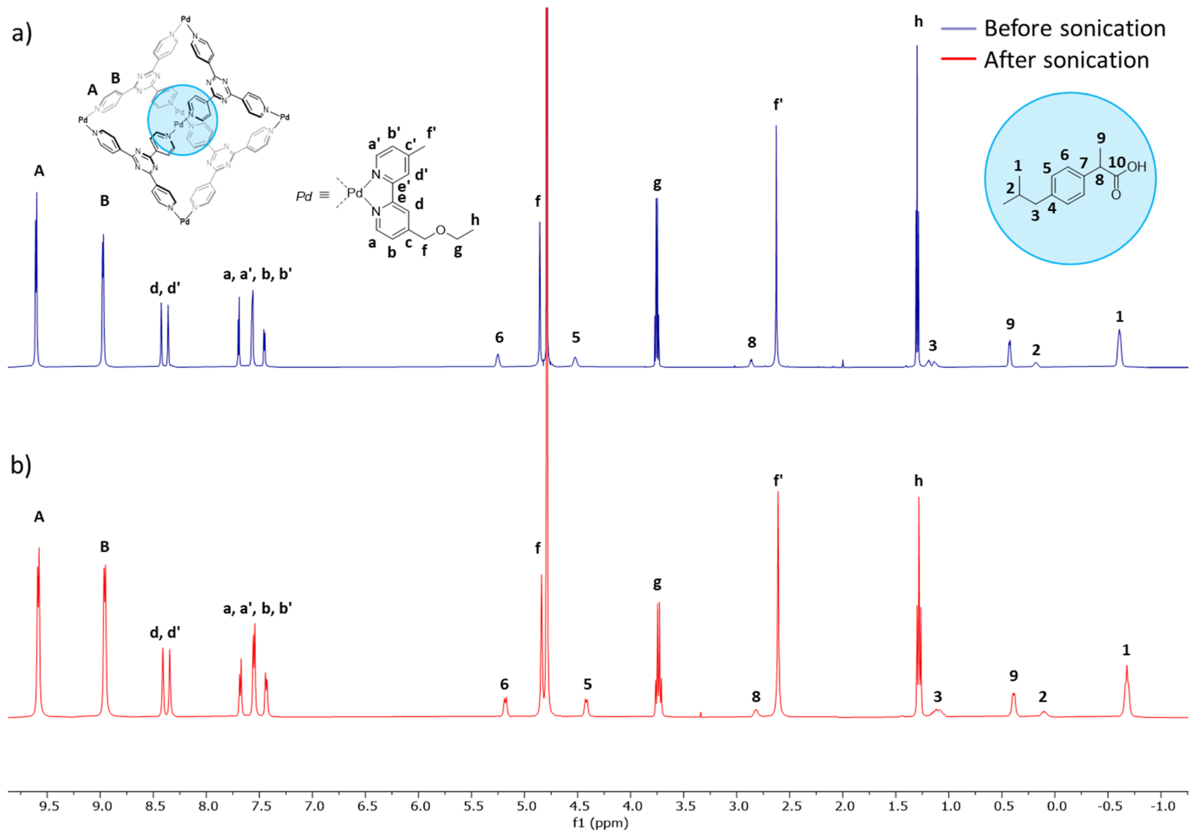

Figure S 12: a) [1]*H NMR (D*$_2$*O) of the cage* ***2•(ibuprofen)***$_2$ *before (blue) and b) after sonication (red). Sonicated for 2 h (1 s on, 1 s off). It can be seen that no guest was released.*

GPC chromatogram in $CHCl_3$

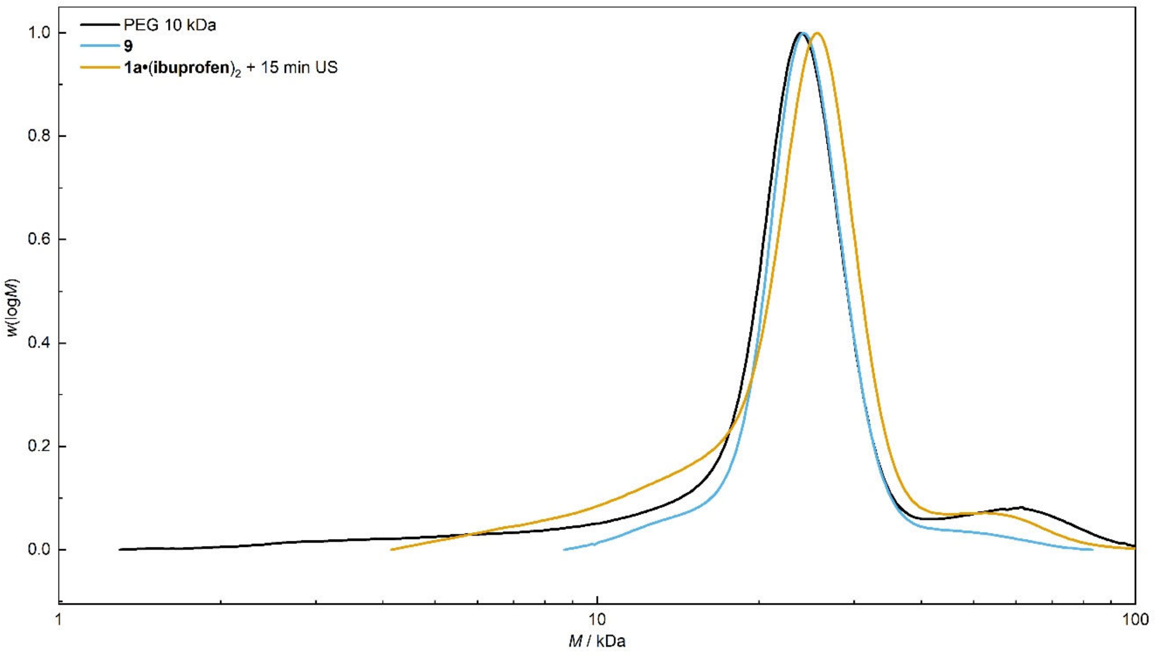

Figure S 13: GPC chromatogram were obtained in CHCl$_3$ *via the RI detector; pristine PEG 10 kDa, cage building block* ***9*** *were not sonicated and* ***1a•(ibuprofen)***$_2$ *was sonicated for only 15 min.*

Spectra

Synthesis

2,4,6-Tris(4-pyridyl)-1,3,5-triazine

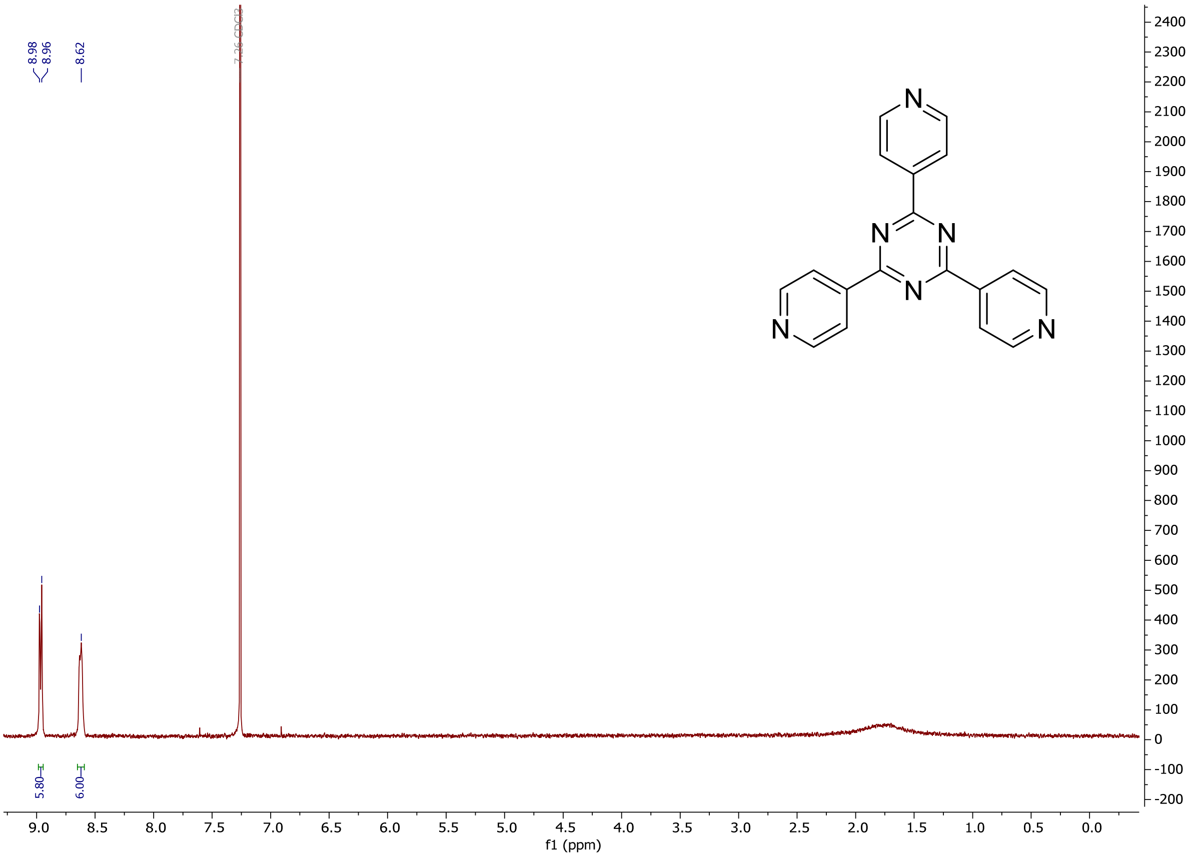

*Figure S 14: ^{1}H NMR (300 MHz, $CDCl_3$) of 2,4,6-tris(4-pyridyl)-1,3,5-triazine (**3**).*

4-Hydroxymethyl-4'-methyl-2,2'-bipyridine

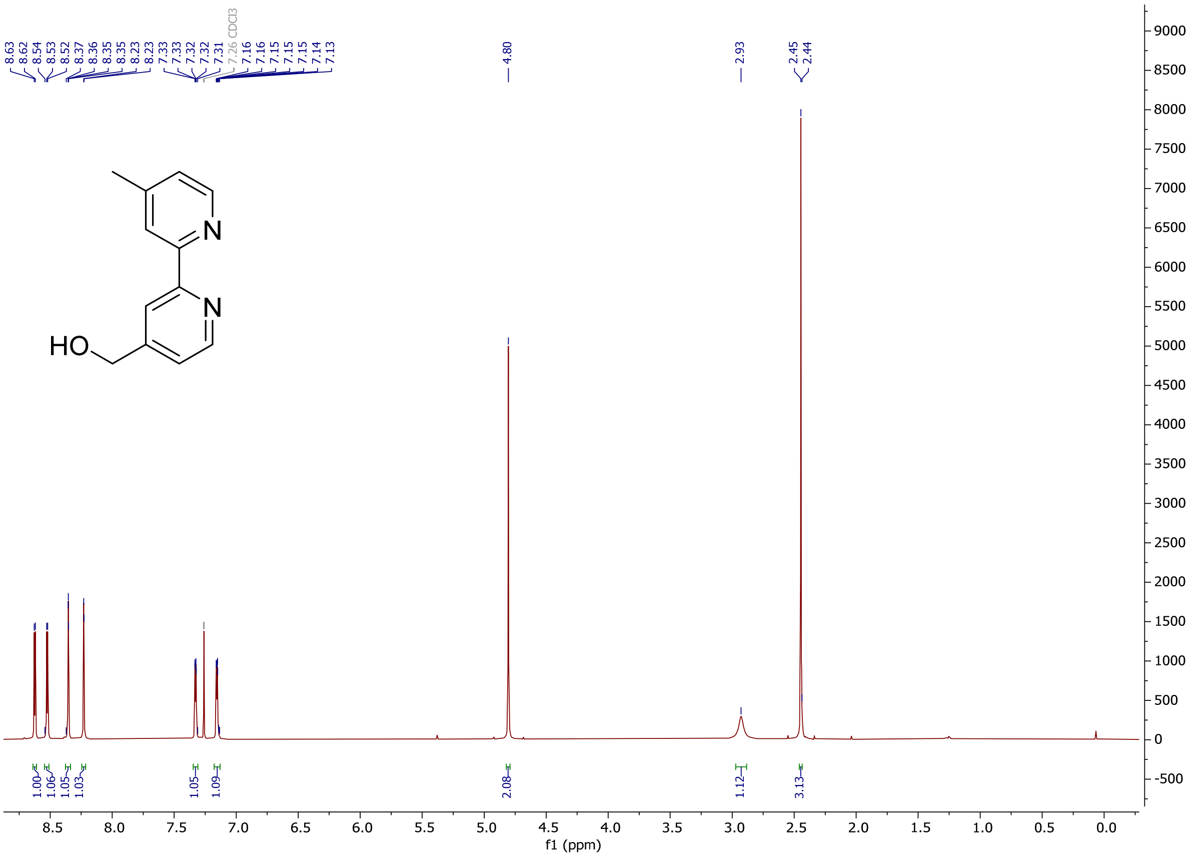

*Figure S 15: ^{1}H NMR (300 MHz, $CDCl_3$) of 4-hydroxymethyl-4'-methyl-2,2'-bipyridine (**4**).*

4-Bromomethyl-4’-methyl-2,2’-bipyridine

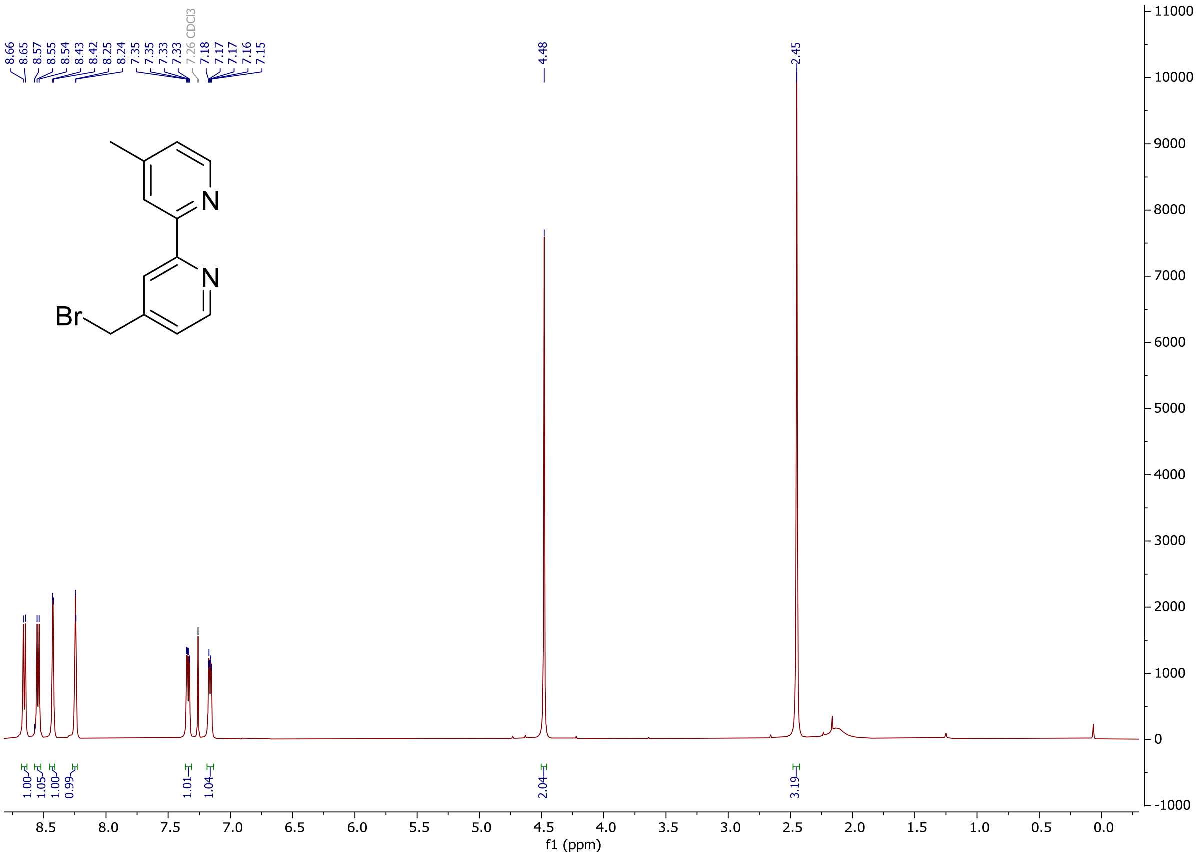

*Figure S 16: ^{1}H NMR (300 MHz, $CDCl_3$) of 4-bromomethyl-4’-methyl-2,2’-bipyridine (**5**).*

4-Ethoxymethyl-4’-methyl-2,2’-bipyridine

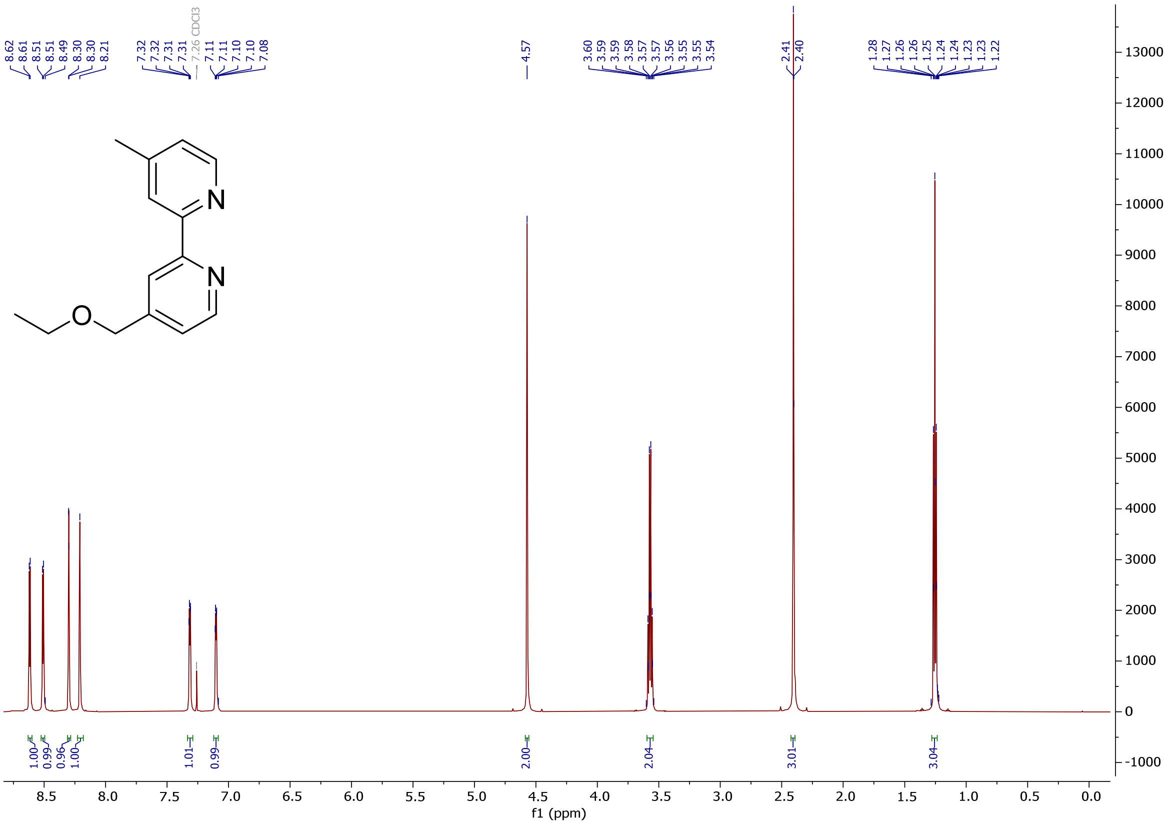

*Figure S 17: ^{1}H NMR (300 MHz, $CDCl_3$) of 4-ethoxymethyl-4’-methyl-2,2’-bipyridine (**6**).*

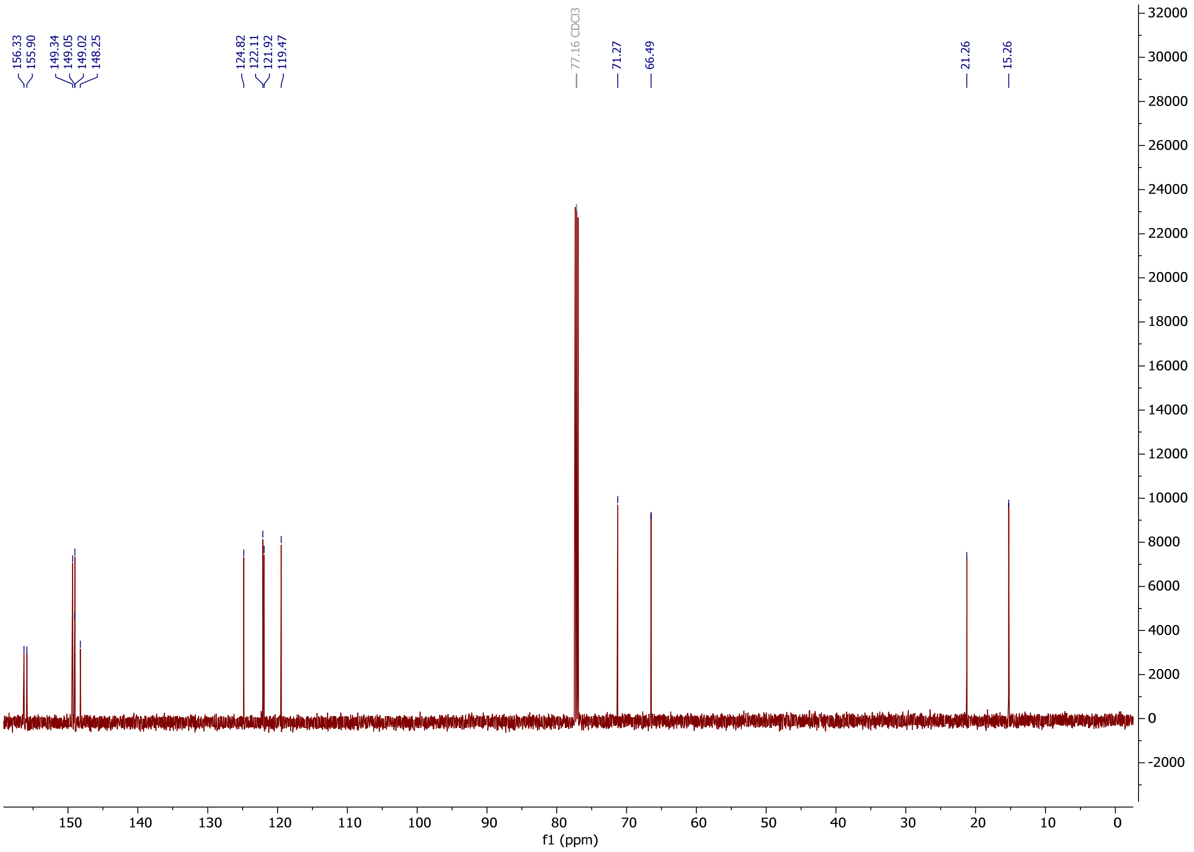

*Figure S 18: $^{13}C\{^{1}H\}$ NMR (151 MHz, $CDCl_3$) of 4-ethoxymethyl-4'-methyl-2,2'-bipyridine (**6**).*

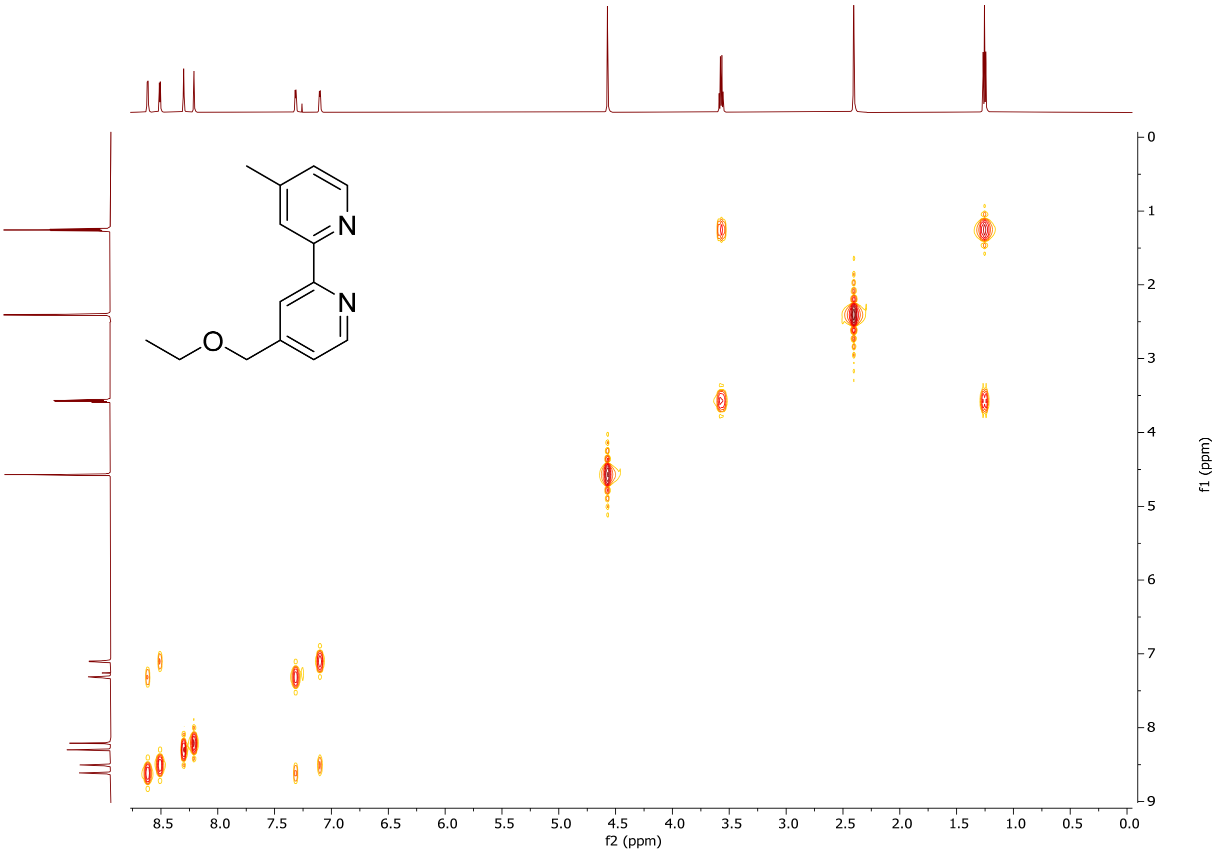

*Figure S 19: ^{1}H-^{1}H COSY NMR (600 MHz, $CDCl_3$) of 4-ethoxymethyl-4'-methyl-2,2'-bipyridine (**6**).*

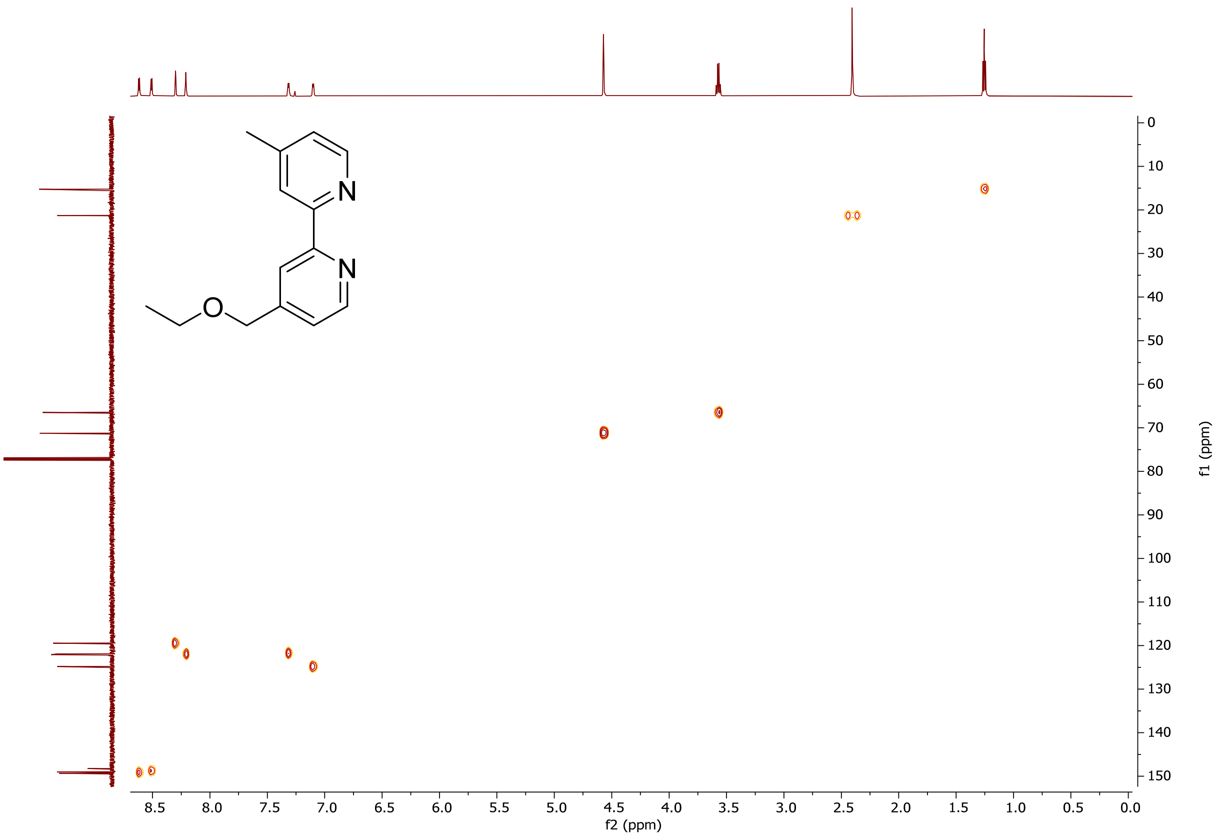

*Figure S 20: ^{1}H-{^{13}C} HSQC NMR (600 MHz, $CDCl_3$) of 4-ethoxymethyl-4'-methyl-2,2'-bipyridine (**6**).*

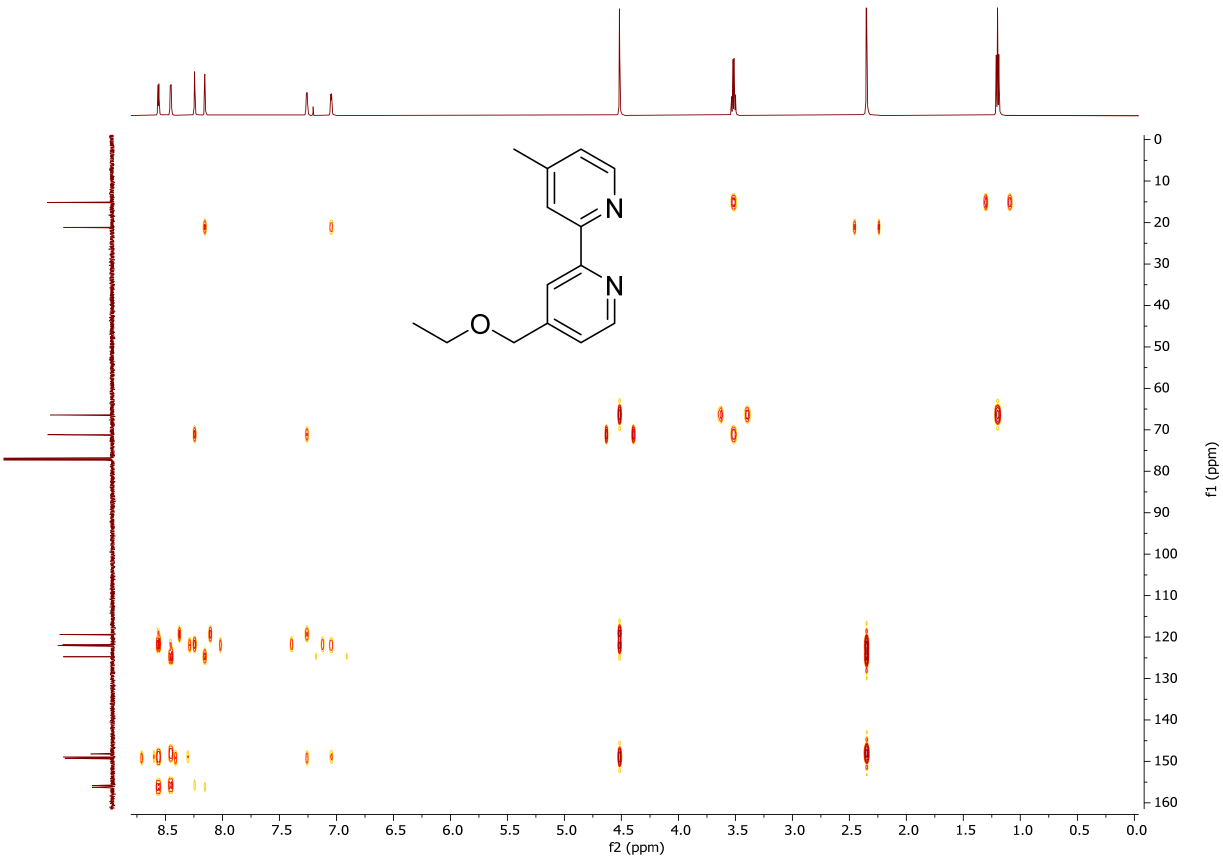

*Figure S 21: ^{1}H-{^{13}C} HMBC NMR (600 MHz, $CDCl_3$) of 4-ethoxymethyl-4'-methyl-2,2'-bipyridine (**6**).*

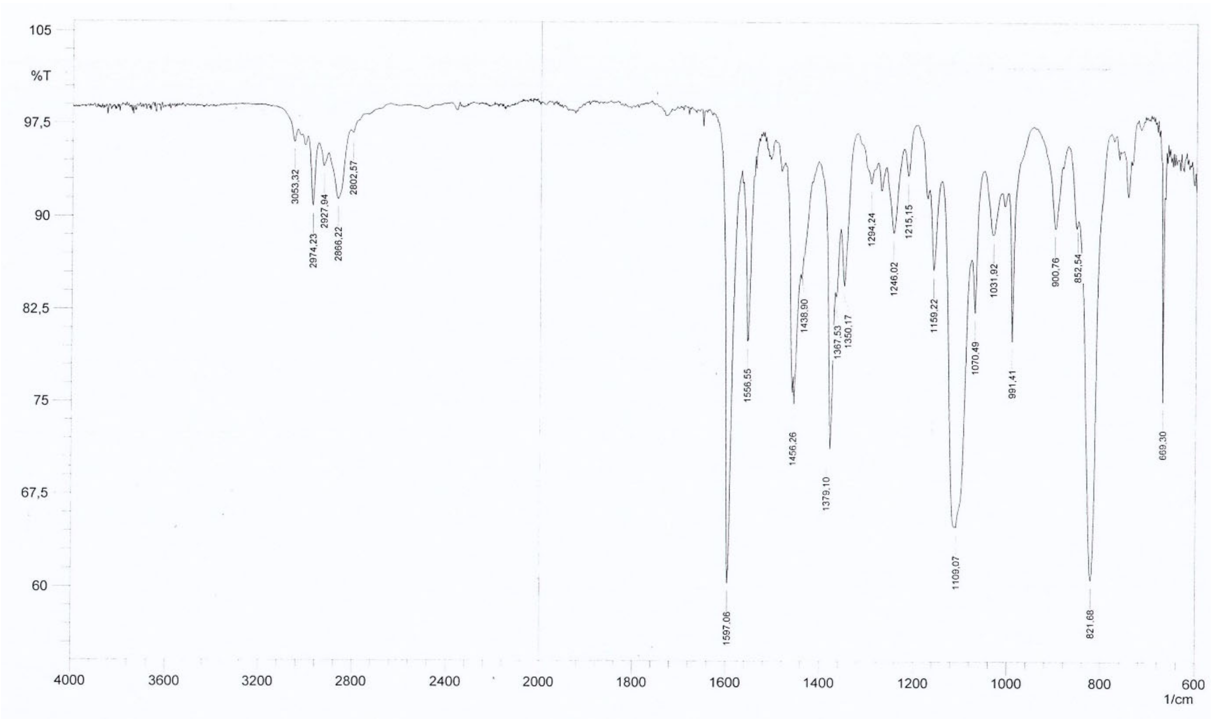

*Figure S 22: IR spectrum of 4-ethoxymethyl-4'-methyl-2,2'-bipyridine (**6**).*

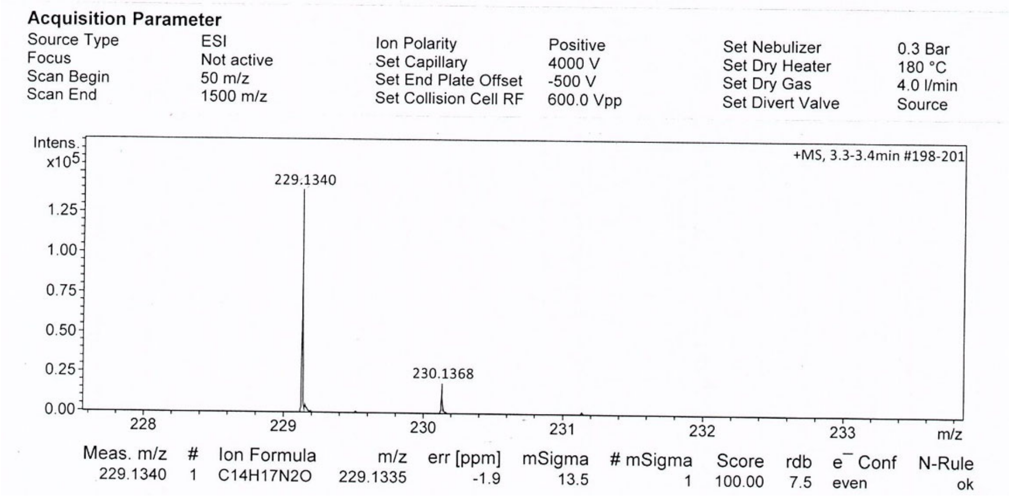

*Figure S 23: HRMS (ESI) spectrum of 4-ethoxymethyl-4'-methyl-2,2'-bipyridine (**6**).*

(4-Ethoxymethyl-4’-methyl-2,2’-bipyridine)-dichloropalladium(II) (7)

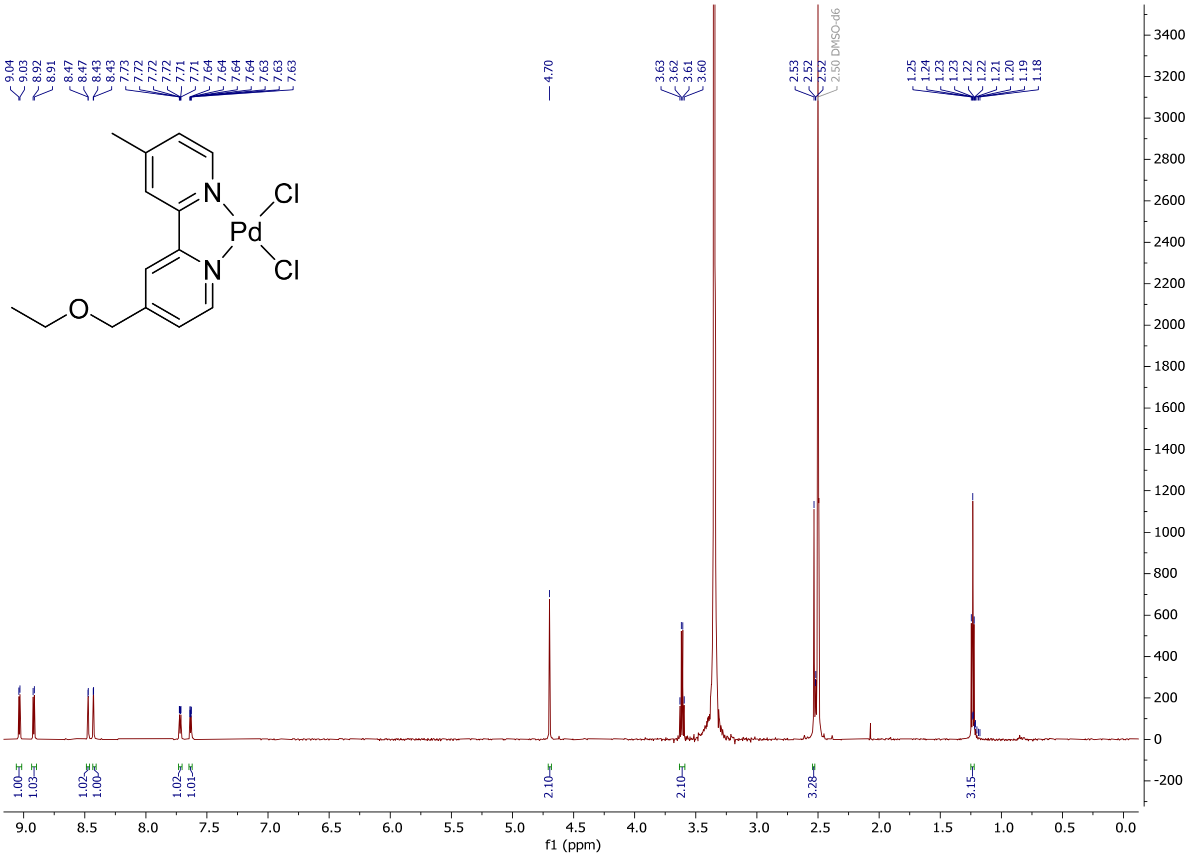

*Figure S 24: ^{1}H NMR (600 MHz, DMSO-d_6) of (4-ethoxymethyl-4’-methyl-2,2’-bipyridine)-dichloropalladium(II) (**7**).*

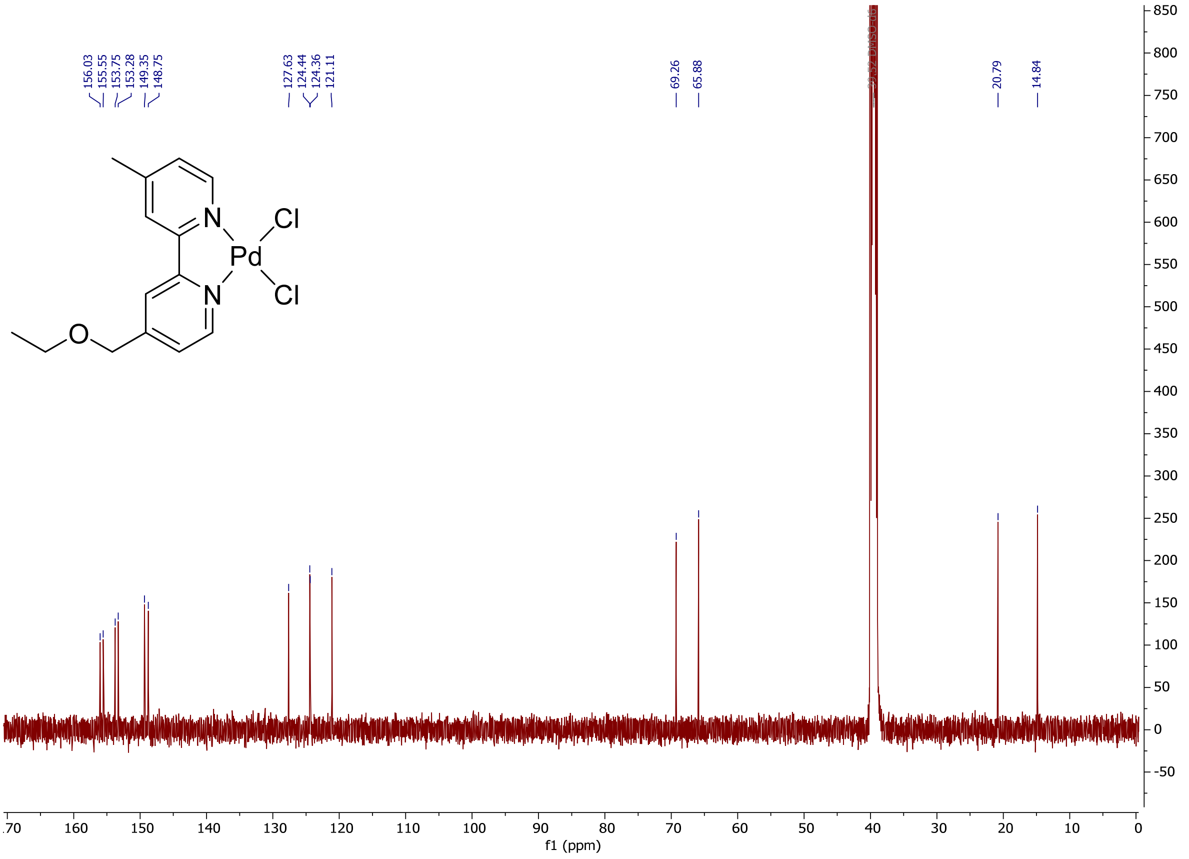

*Figure S 25: $^{13}C\{^{1}H\}$ NMR (126 MHz, d_6-DMSO) of (4-ethoxymethyl-4’-methyl-2,2’-bipyridine)-dichloropalladium(II) (**7**).*

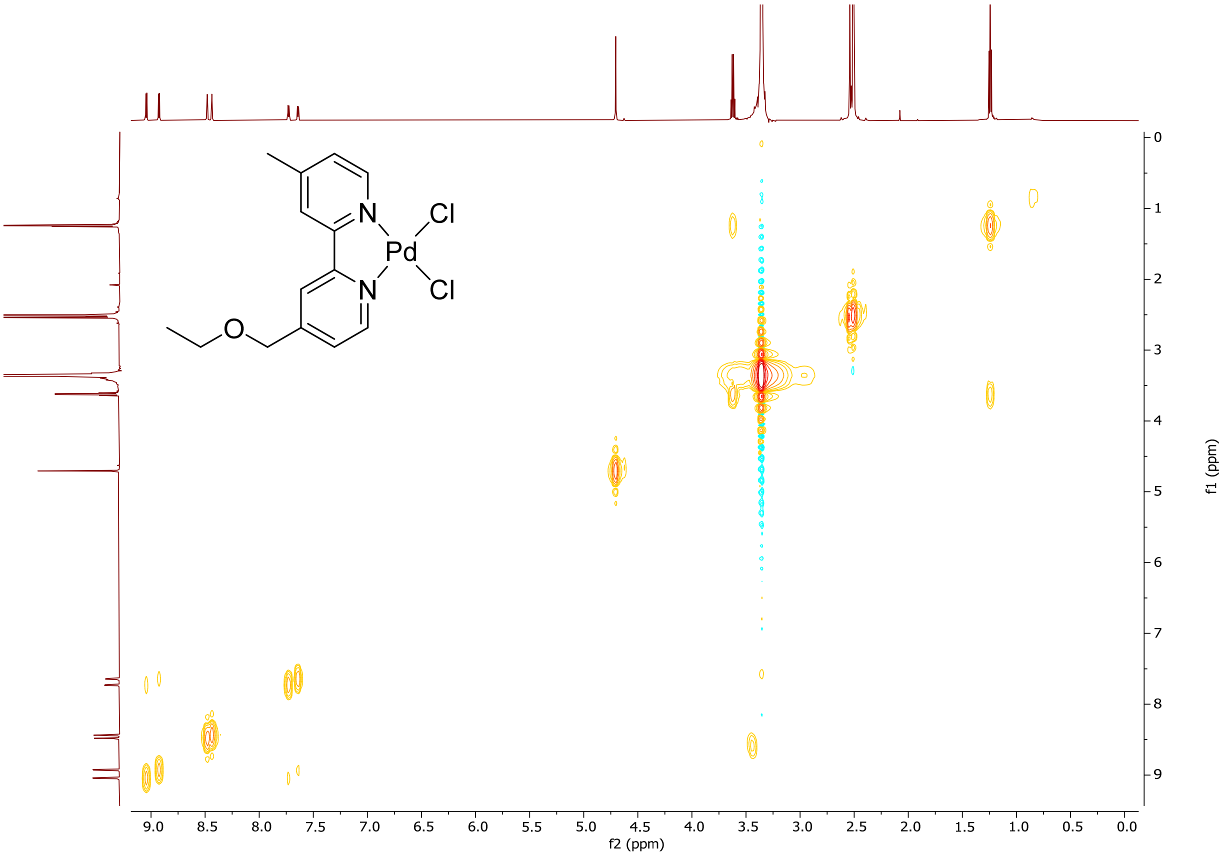

*Figure S 26: ¹H-¹H COSY NMR (600 MHz, DMSO-d_6) of (4-ethoxymethyl-4'-methyl-2,2'-bipyridine)-dichloropalladium(II) (**7**).*

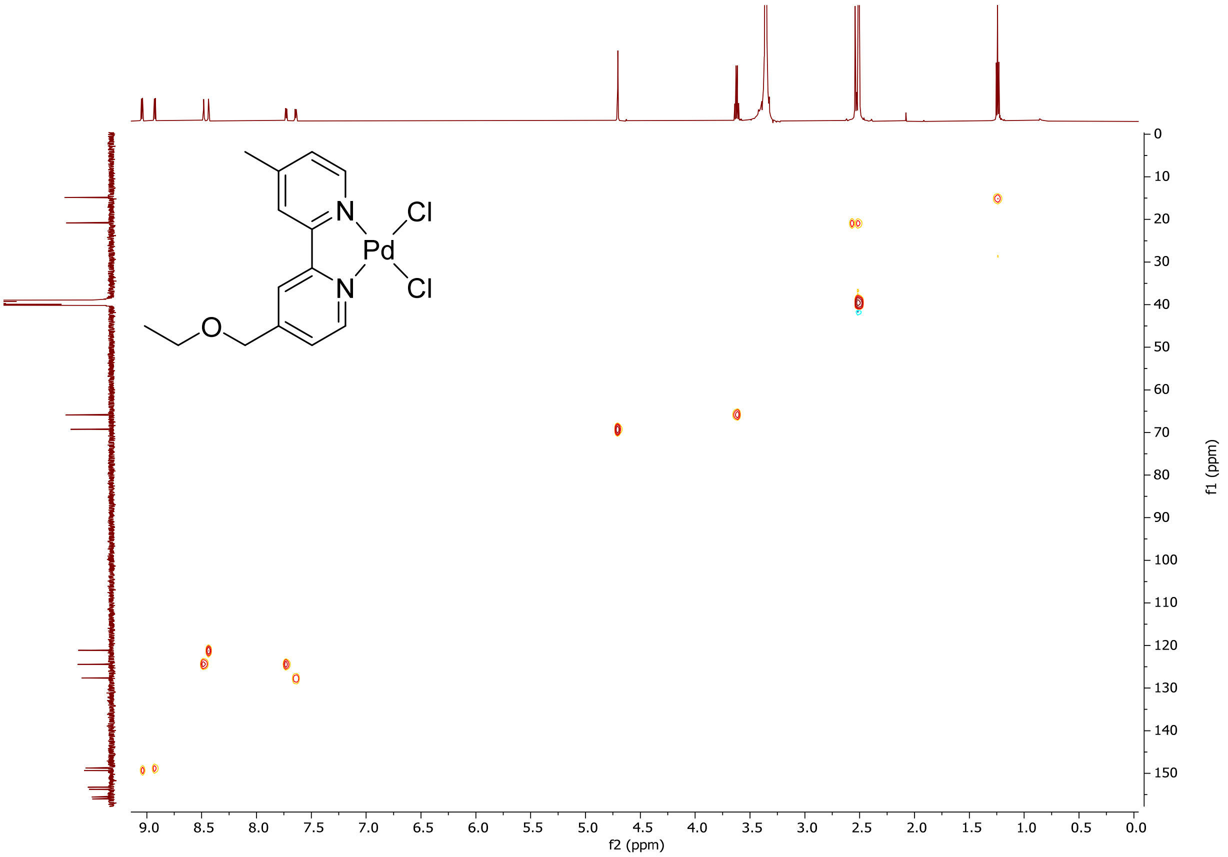

*Figure S 27: ¹H-{¹³C} HSQC NMR (600 MHz, DMSO-d_6) of (4-ethoxymethyl-4'-methyl-2,2'-bipyridine)-dichloropalladium(II) (**7**).*

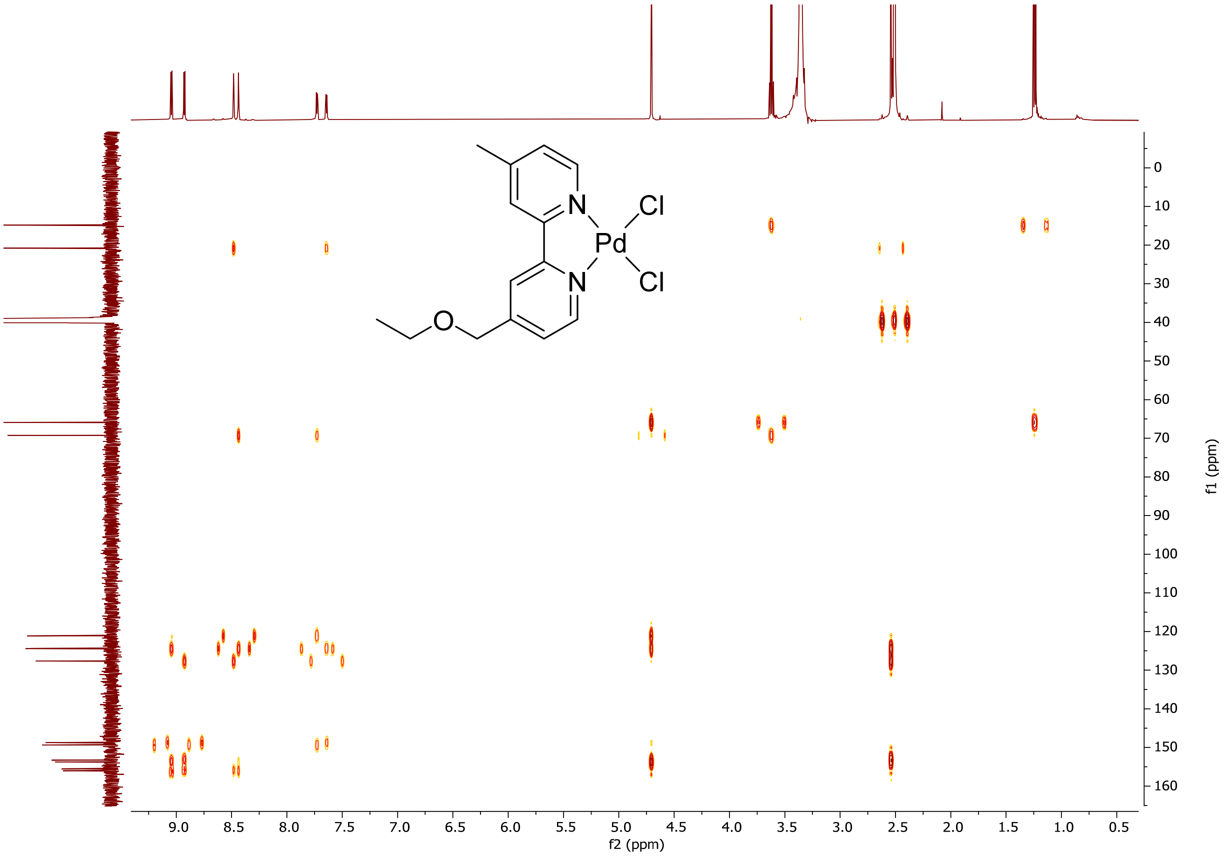

Figure S 28: ^{1}H-{^{13}C} HMBC NMR (600 MHz, $CDCl_3$) of (4-ethoxymethyl-4'-methyl-2,2'-bipyridine)-dichloropalladium(II) (**7**).

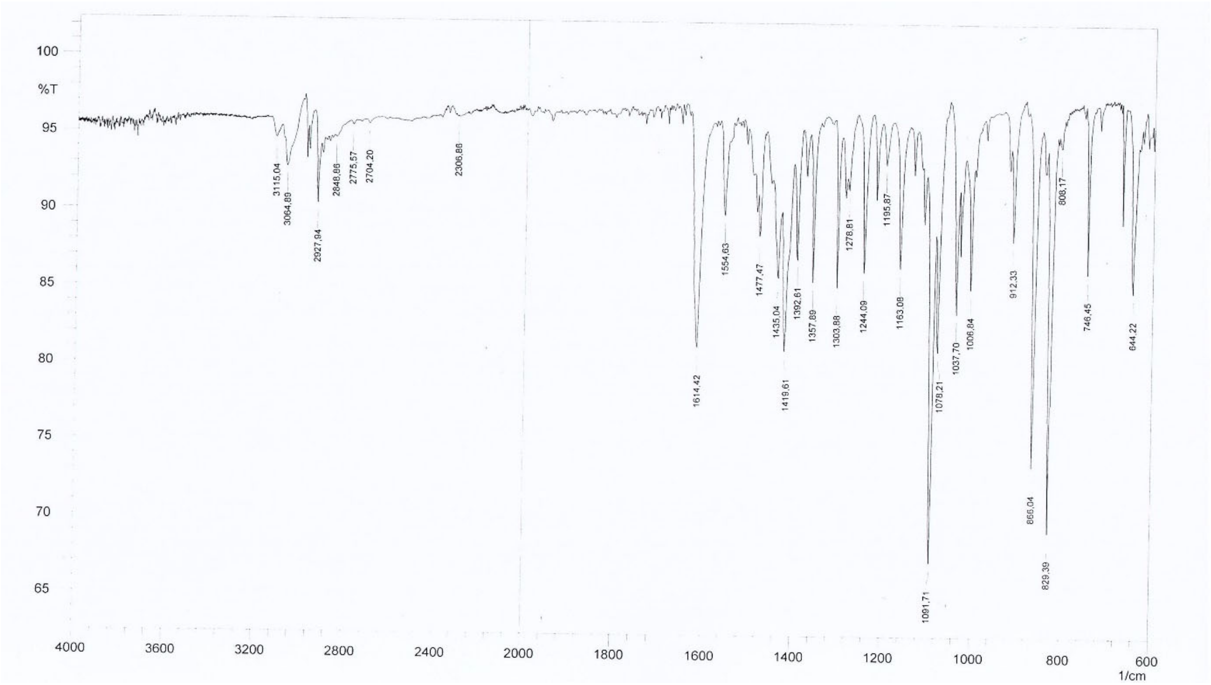

Figure S 29: IR spectrum of (4-ethoxymethyl-4'-methyl-2,2'-bipyridine)-dichloropalladium(II) (**7**).

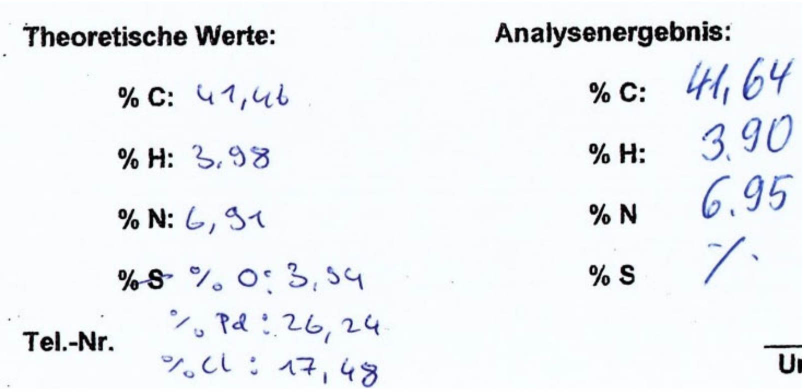

Theoretische Werte:

% C: 41,46

% H: 3,98

% N: 6,91

% ~~S~~ % O: 3,94

% Pd: 26,24

% Cl: 17,48

Tel.-Nr.

Analysenergebnis:

% C: 41,64

% H: 3,90

% N 6,95

% S /.

Ui

*Figure S 30: Elemental analysis of (4-ethoxymethyl-4’-methyl-2,2’-bipyridine)-dichloropalladium(II) (**7**).*

(4-Ethoxymethyl-4’-methyl-2,2’-bipyridine)-dinitratopalladium(II) (8)

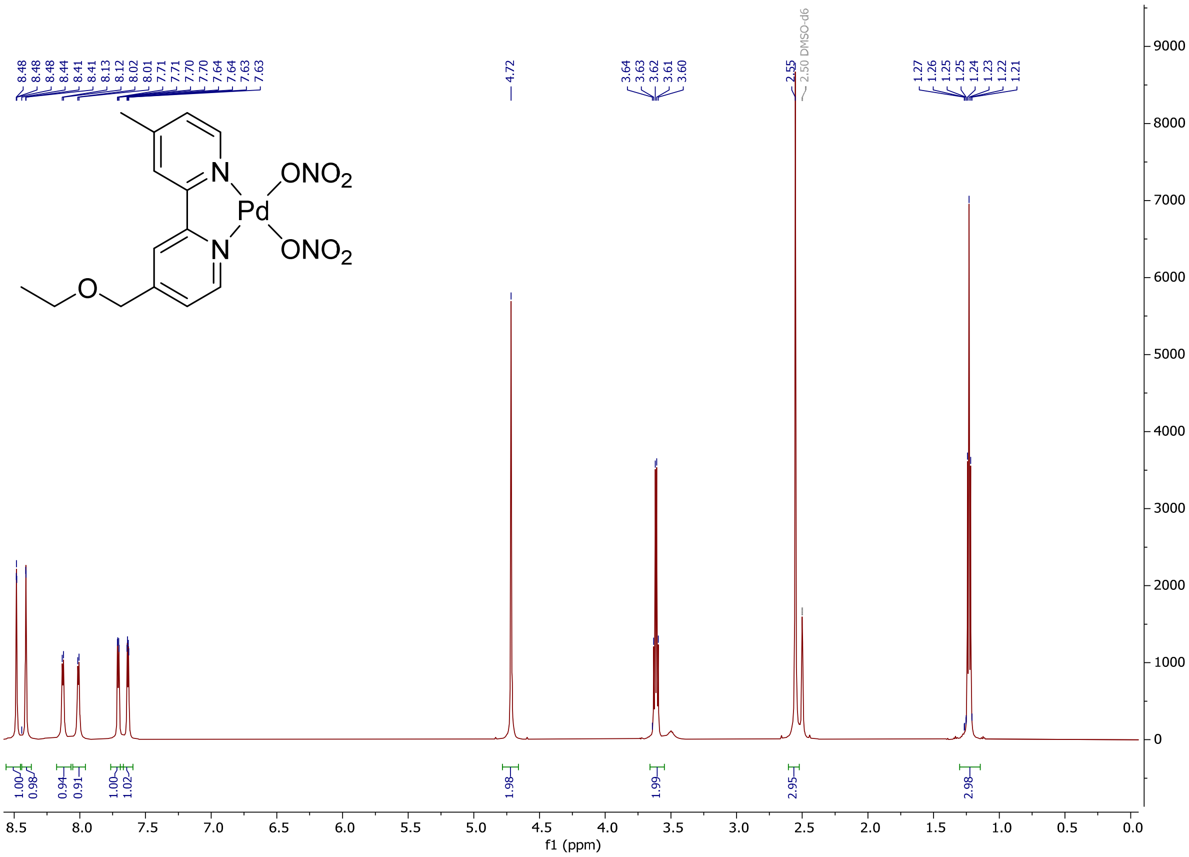

*Figure S 31: ^{1}H NMR (600 MHz, DMSO-d_6) of (4-ethoxymethyl-4’-methyl-2,2’-bipyridine)-dinitratopalladium(II) (**8**).*

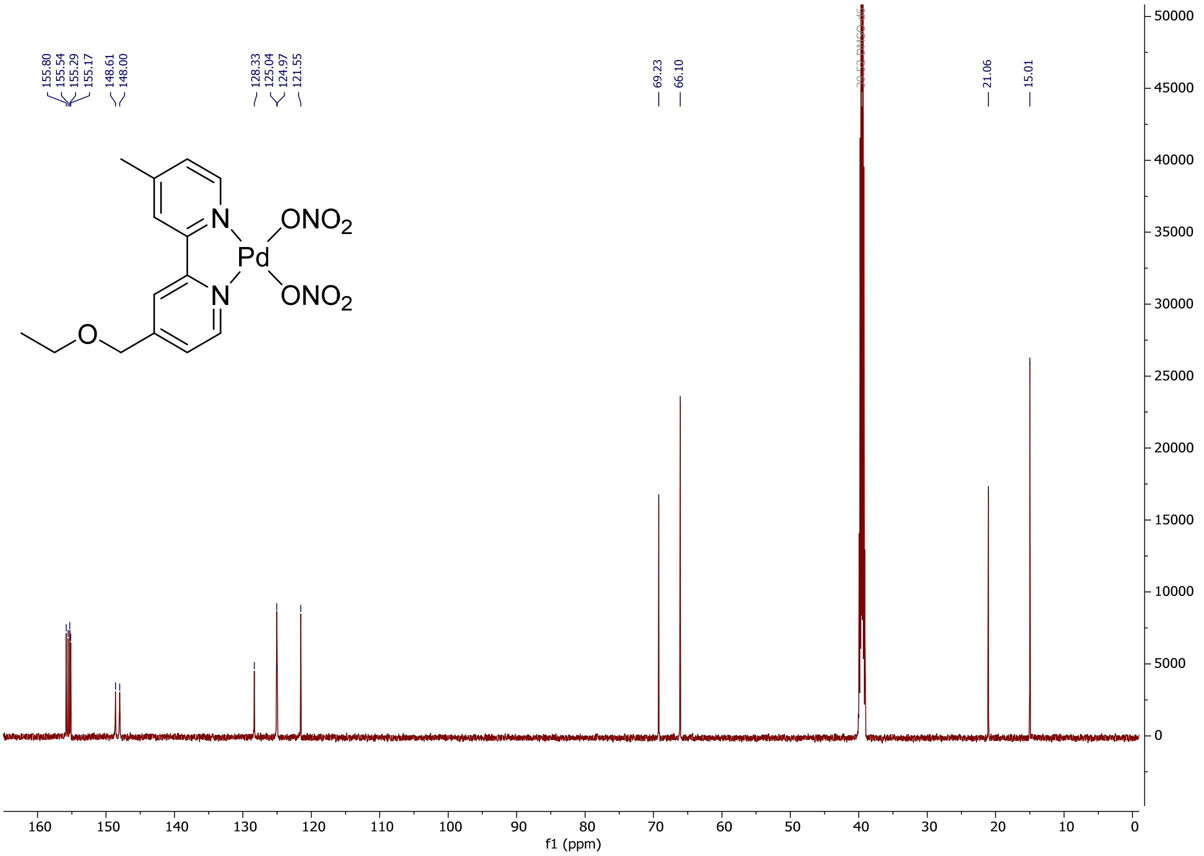

*Figure S 32: $^{13}C\{^{1}H\}$ NMR (151 MHz, DMSO-d6) of (4-ethoxymethyl-4'-methyl-2,2'-bipyridine)-dinitratopalladium(II) (**8**).*

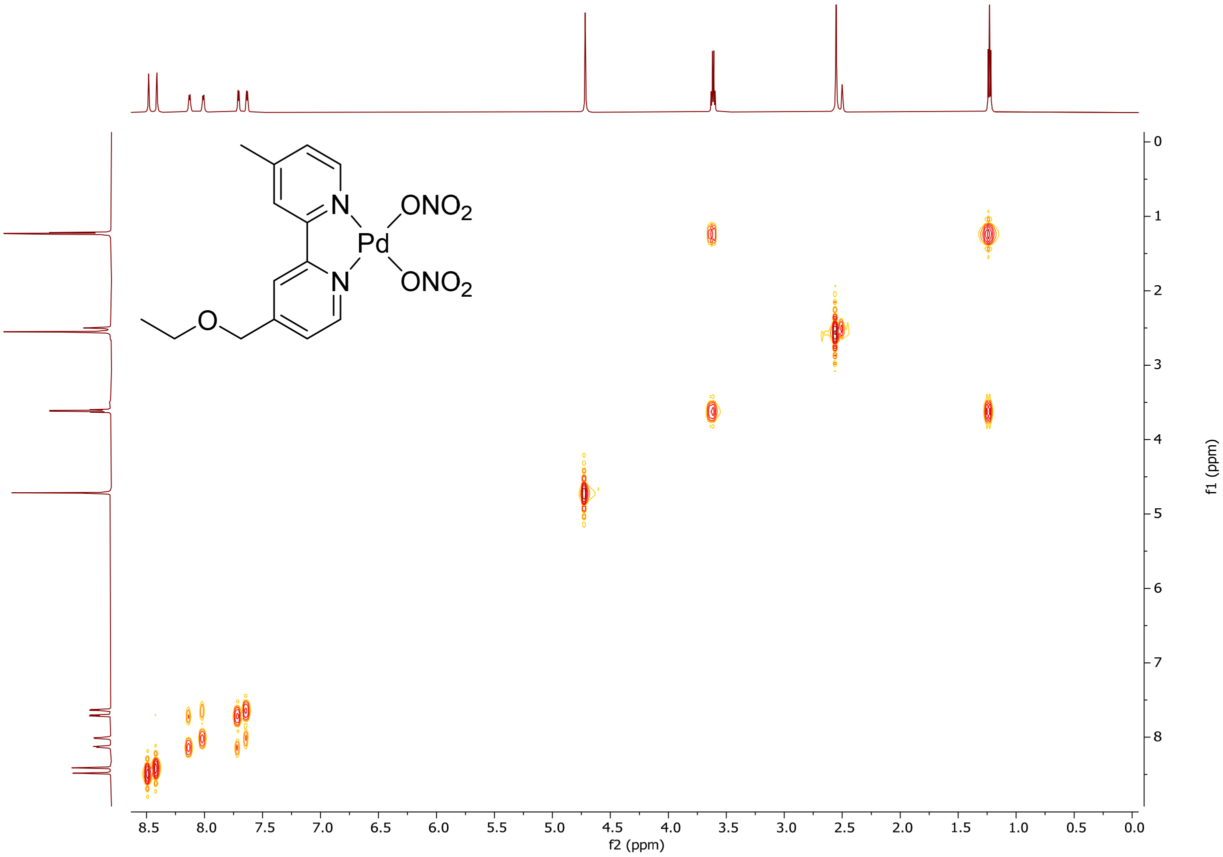

*Figure S 33: ^{1}H-^{1}H COSY NMR (600 MHz, DMSO-d_6) of (4-ethoxymethyl-4'-methyl-2,2'-bipyridine)-dinitratopalladium(II) (**8**).*

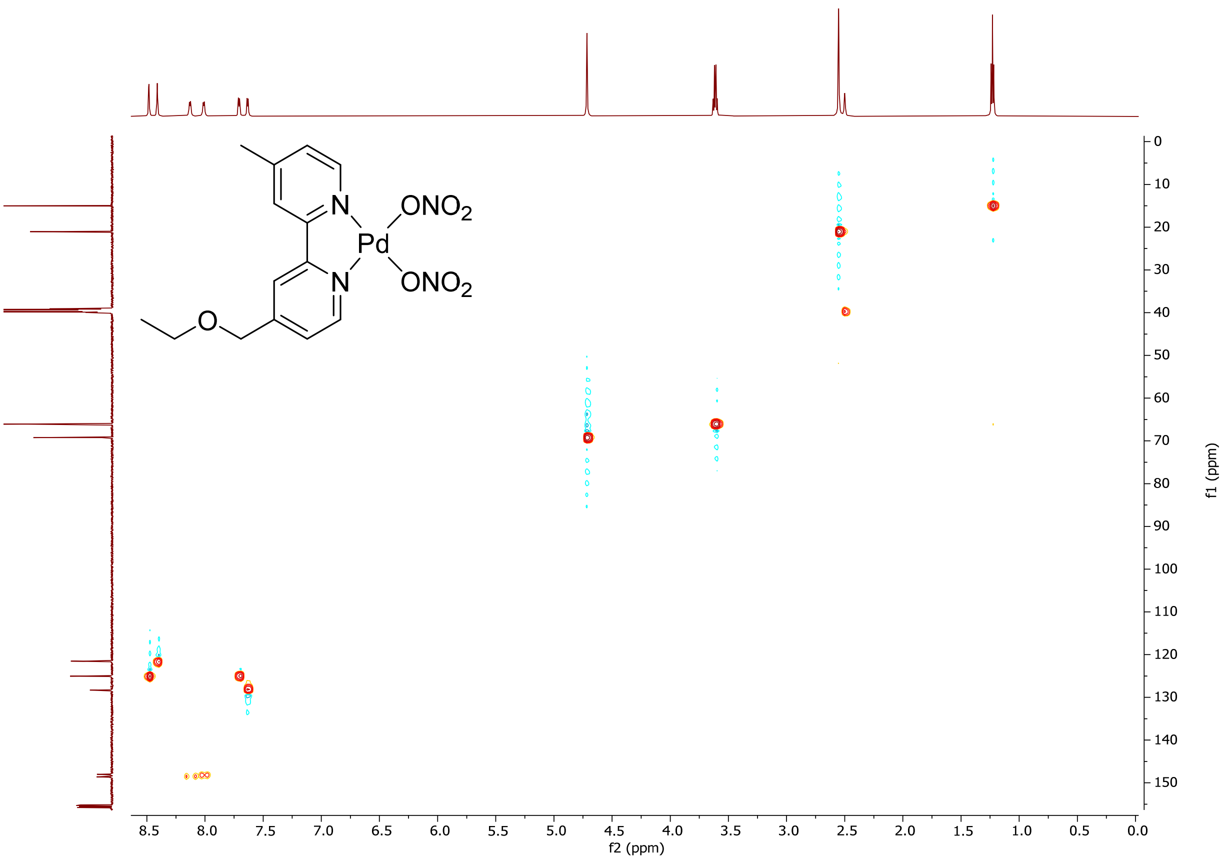

*Figure S 34: ^{1}H-{^{13}C} HSQC NMR (600 MHz, DMSO-d_6) of (4-ethoxymethyl-4'-methyl-2,2'-bipyridine)-dinitratopalladium(II) (**8**).*

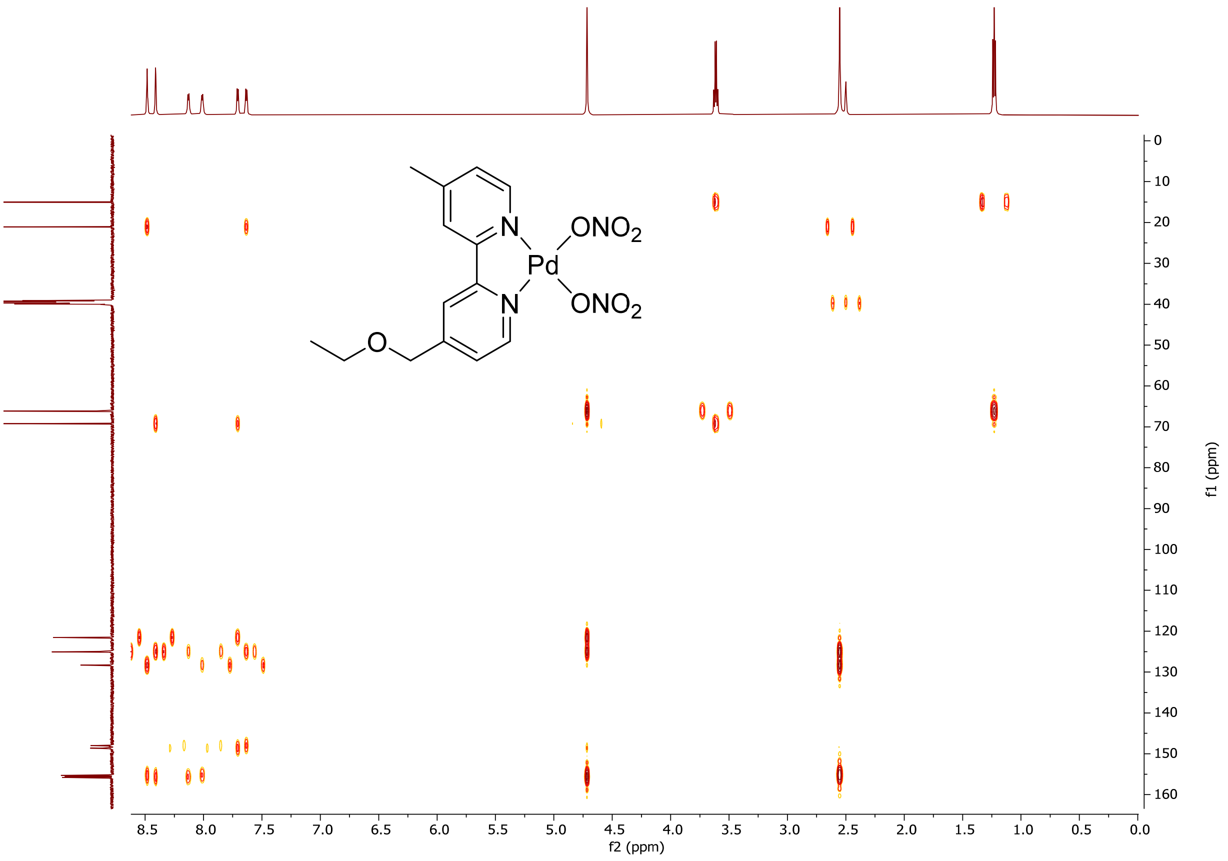

*Figure S 35: ^{1}H-{^{13}C} HMBC NMR (600 MHz, DMSO-d_6) of (4-ethoxymethyl-4'-methyl-2,2'-bipyridine)-dinitratopalladium(II) (**8**).*

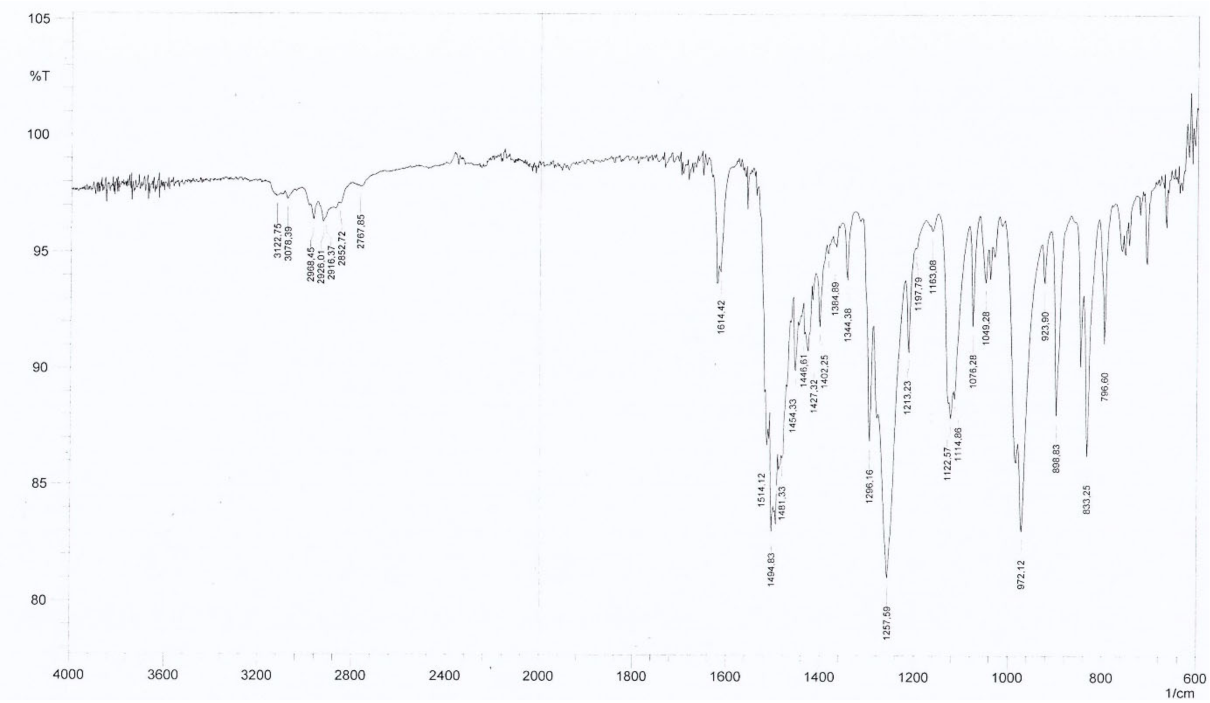

*Figure S 36: IR spectrum of (4-ethoxymethyl-4'-methyl-2,2'-bipyridine)-dinitratopalladium(II) (**8**).*

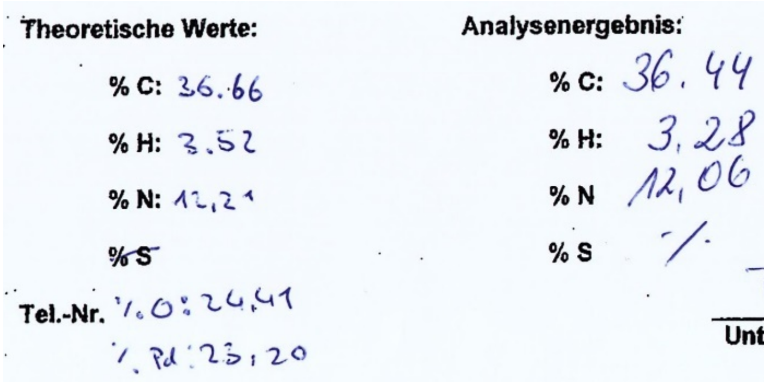

Theoretische Werte:		Analysenergebnis:	
% C:	36.66	% C:	36.44
% H:	3.52	% H:	3,28
% N:	12,21	% N	12,06
~~% S~~		% S	./.
Tel.-Nr.	% O: 24,41		
	% Pd: 23,20		

*Figure S 37: Elemental analysis of (4-ethoxymethyl-4'-methyl-2,2'-bipyridine)-dinitratopalladium(II) (**8**).*

Cage 2

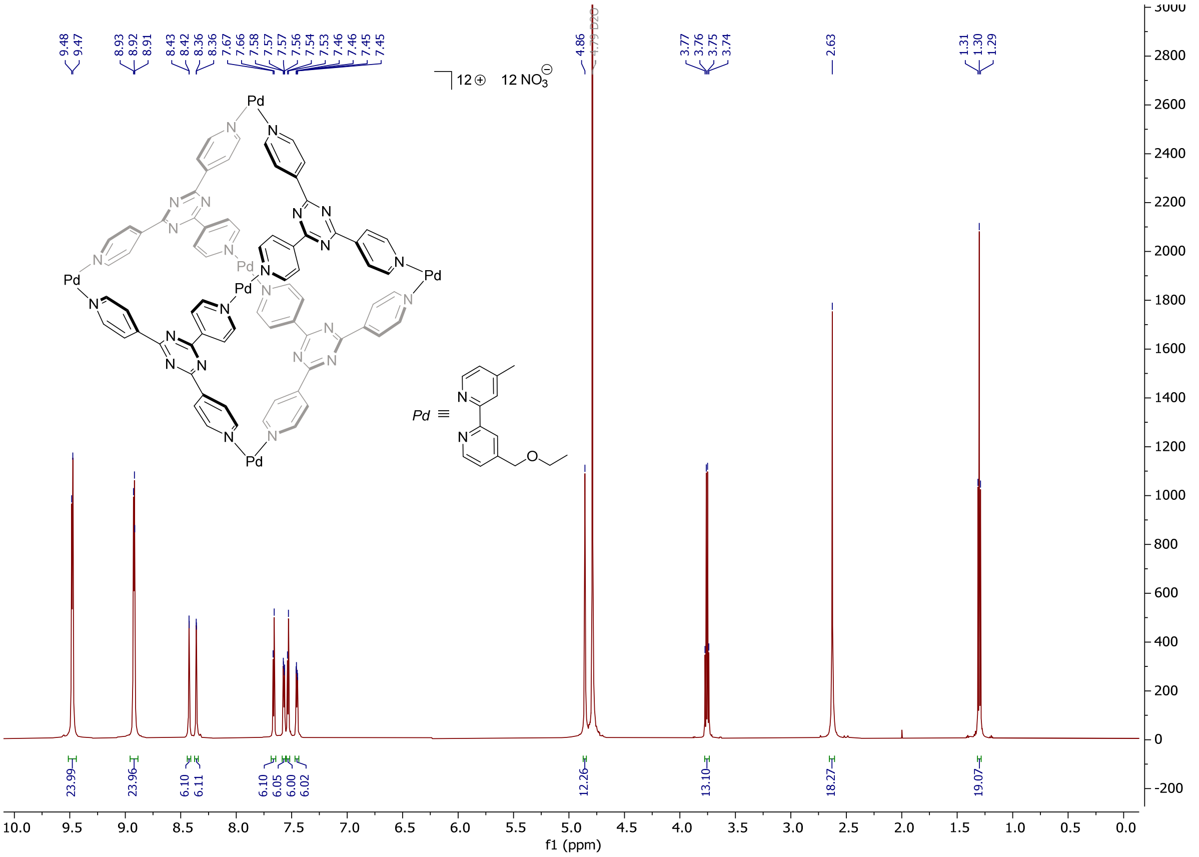

*Figure S 38: ^{1}H NMR (600 MHz, D_2O) of cage **2**.*

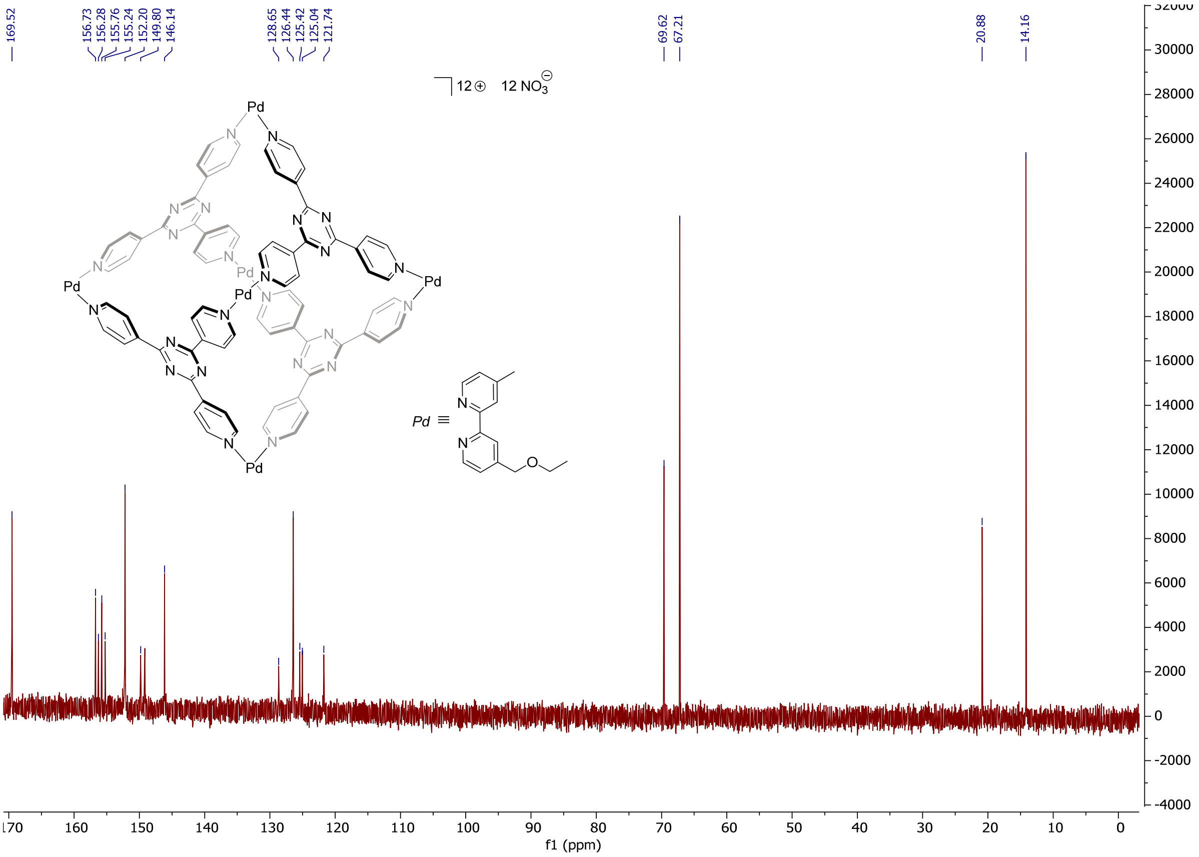

*Figure S 39: $^{13}C\{^{1}H\}$ NMR (151 MHz, D_2O) of cage **2.***

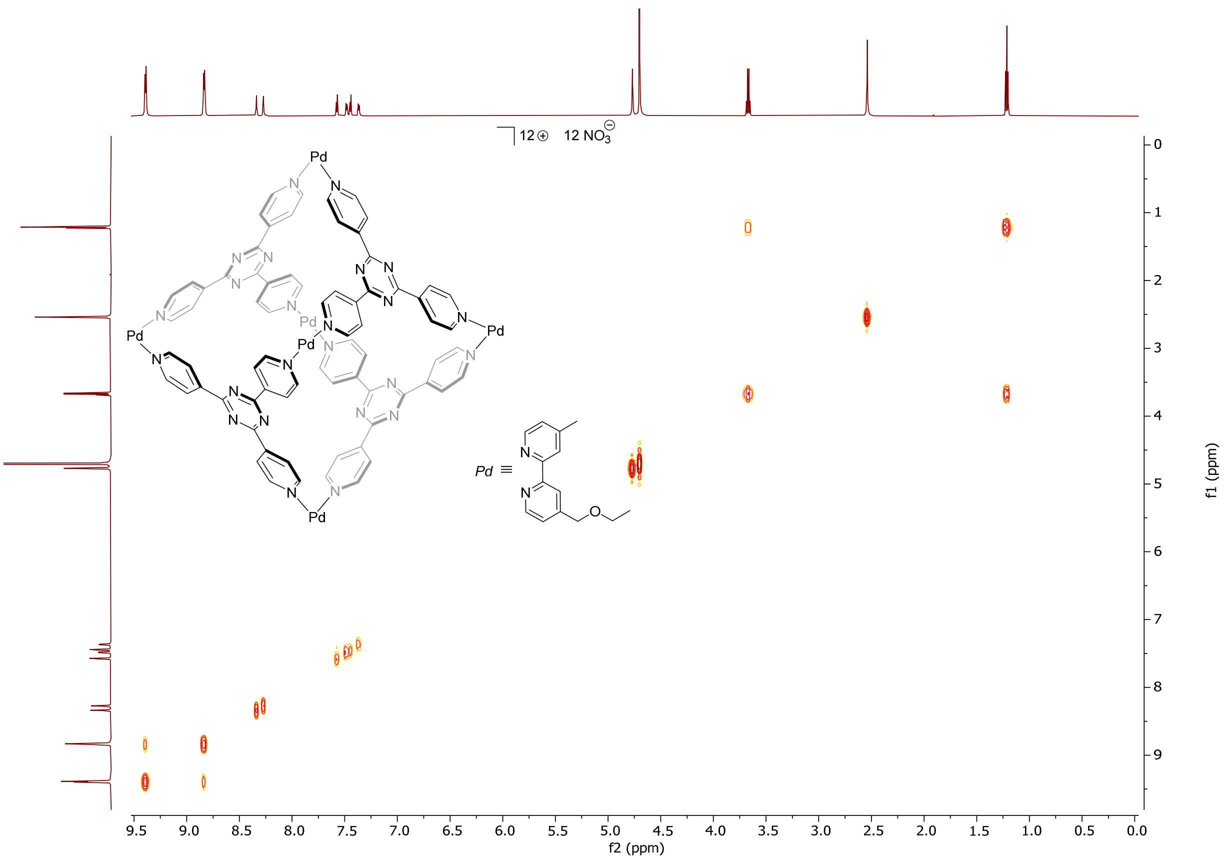

Figure S 40: ^{1}H-^{1}H COSY NMR (600 MHz, D_2O) of cage **2**.

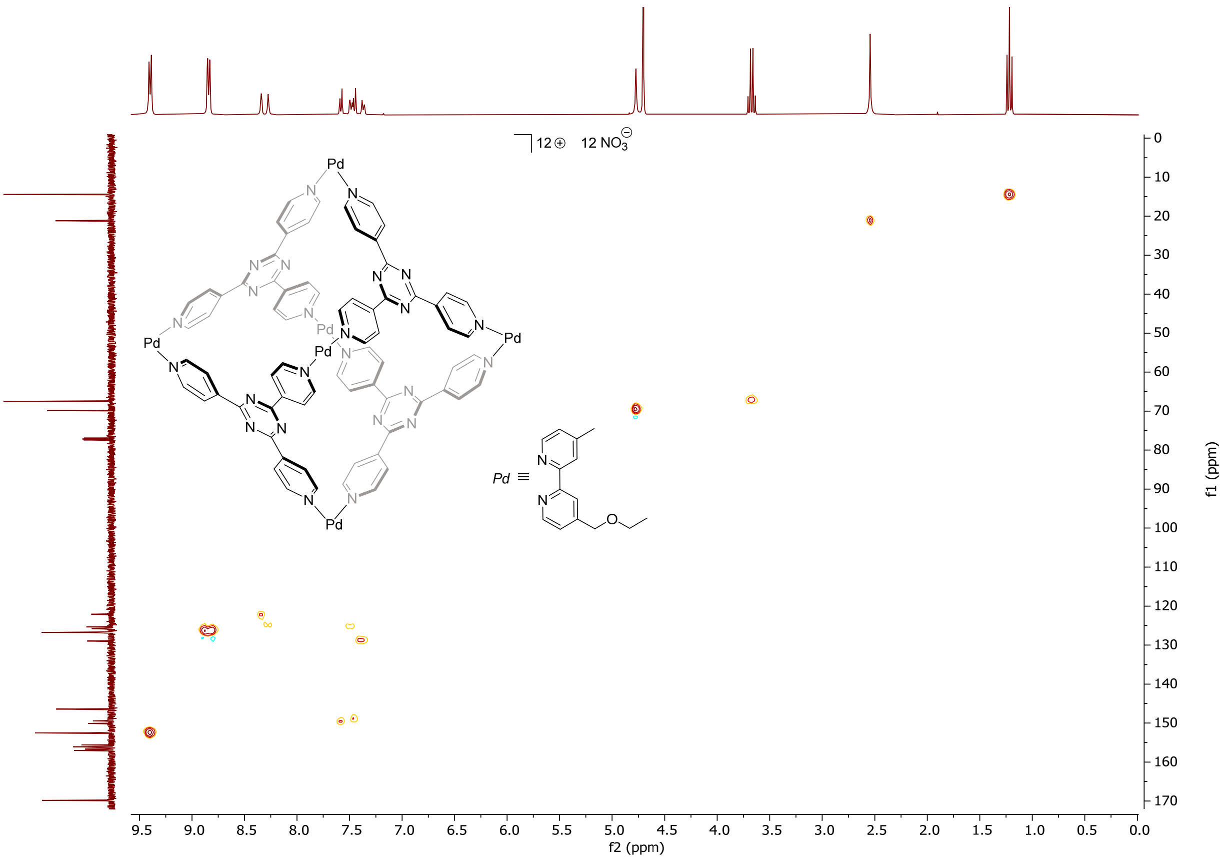

Figure S 41: ^{1}H-{^{13}C} HSQC NMR (600 MHz, D_2O) of cage **2**.

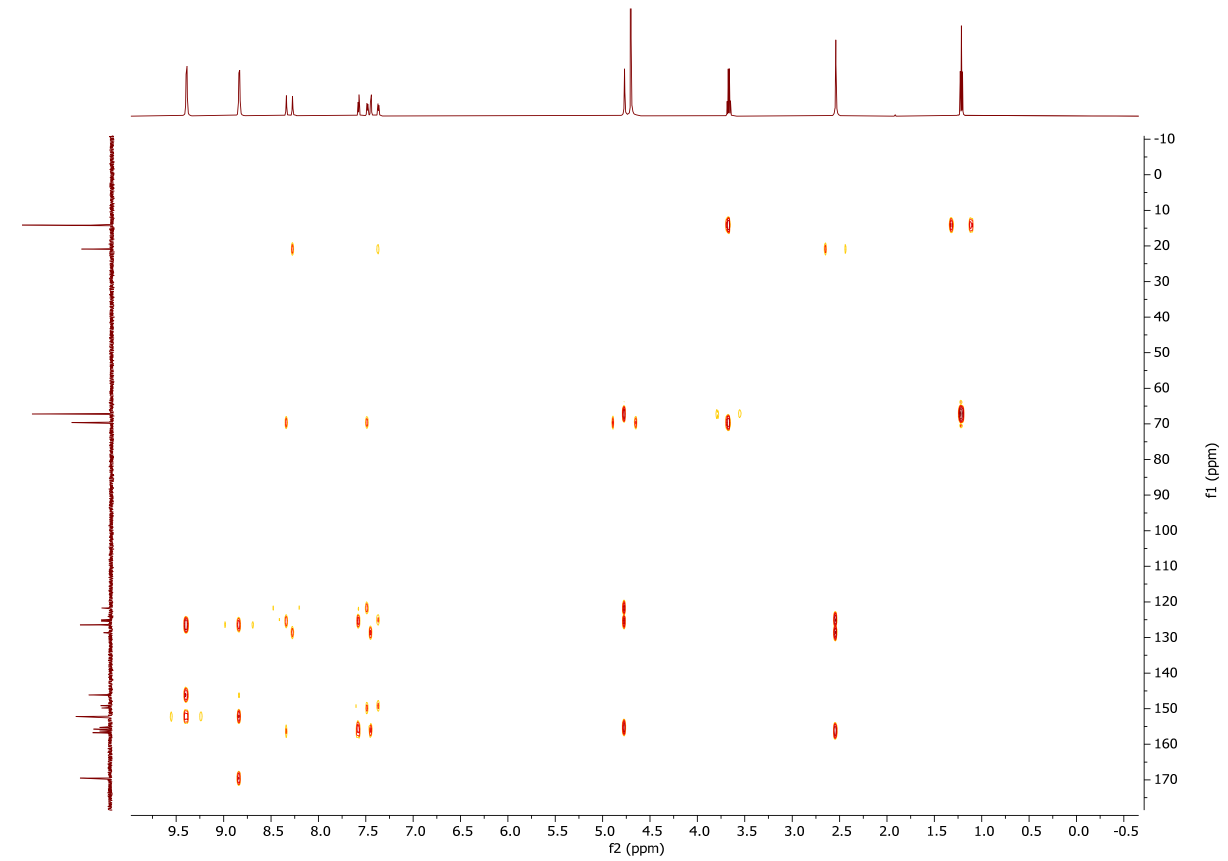

*Figure S 42: ^{1}H-{^{13}C} HMBC NMR (600 MHz, D_2O) of cage **2**.*

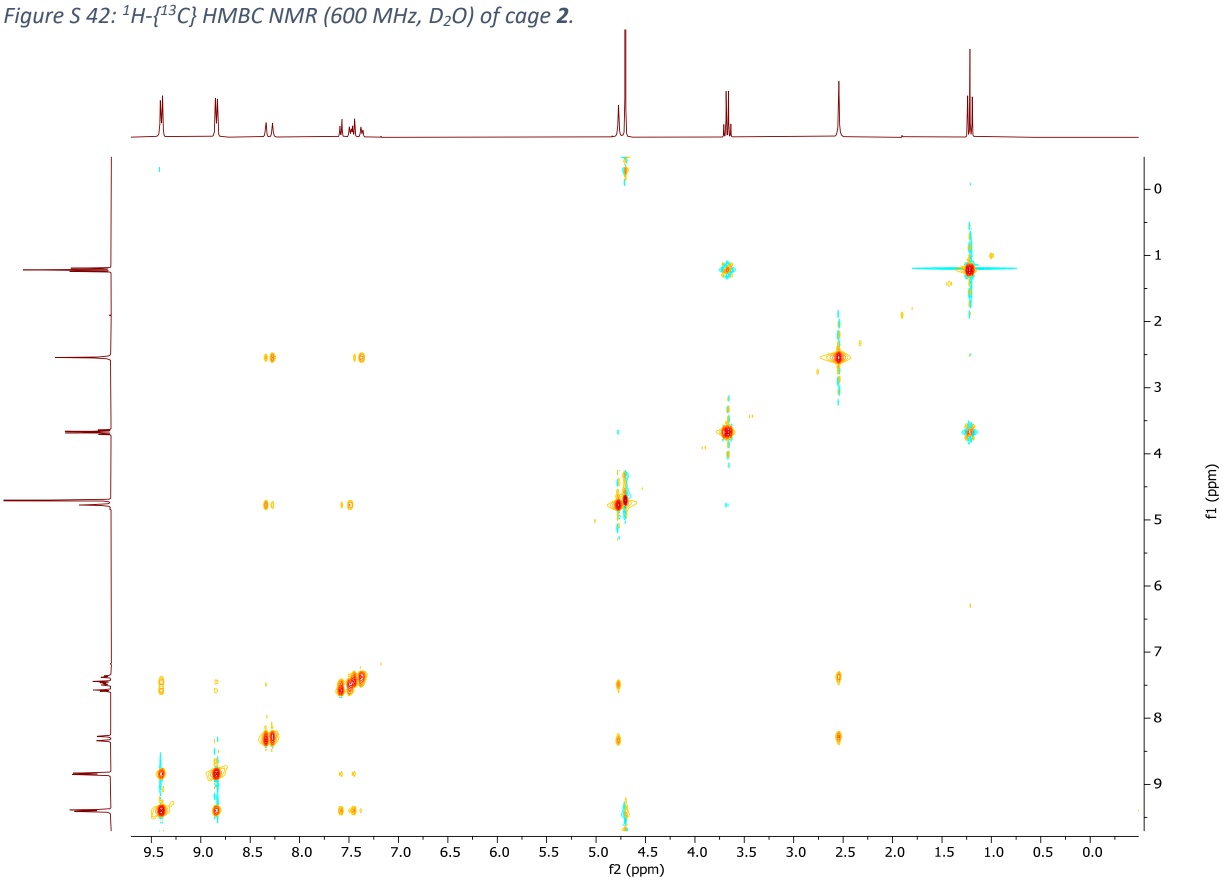

*Figure S 43: ^{1}H-^{1}H NOESY NMR (300 MHz, D_2O) of cage **2**. Baseline correction with Whittaker Smoother: Filter=1, Smooth factor=256.*

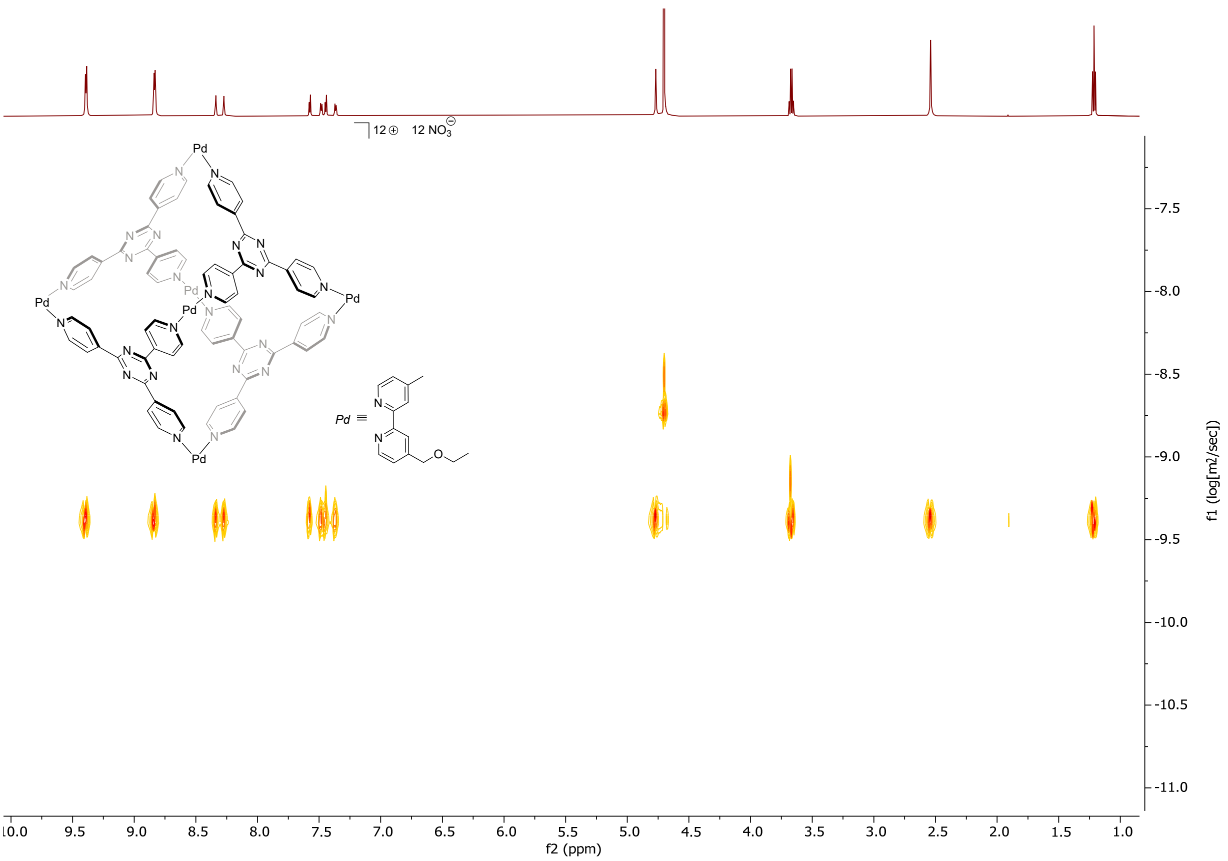

*Figure S 44: DOSY NMR (600 MHz, D$_2$O) of cage **2**.*

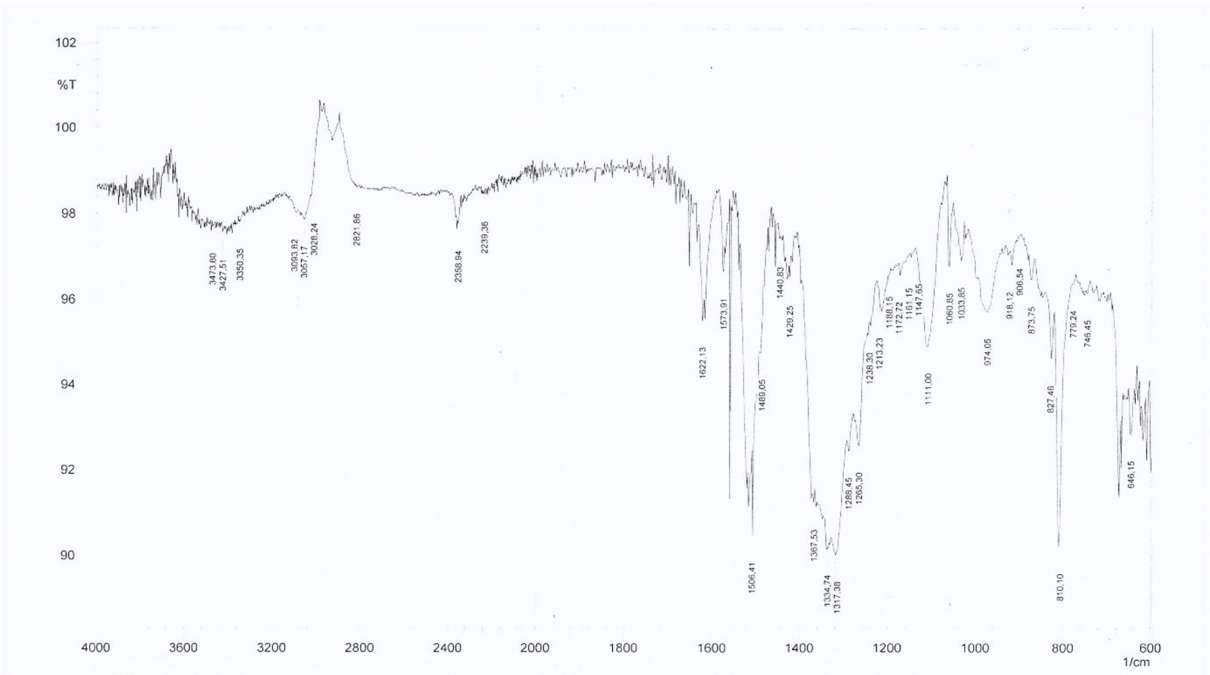

*Figure S 45: IR spectrum of cage **2**.*

4-Methoxypolyethyleneglycolmethyl-4’-methyl-2,2’-bipyridine (9)

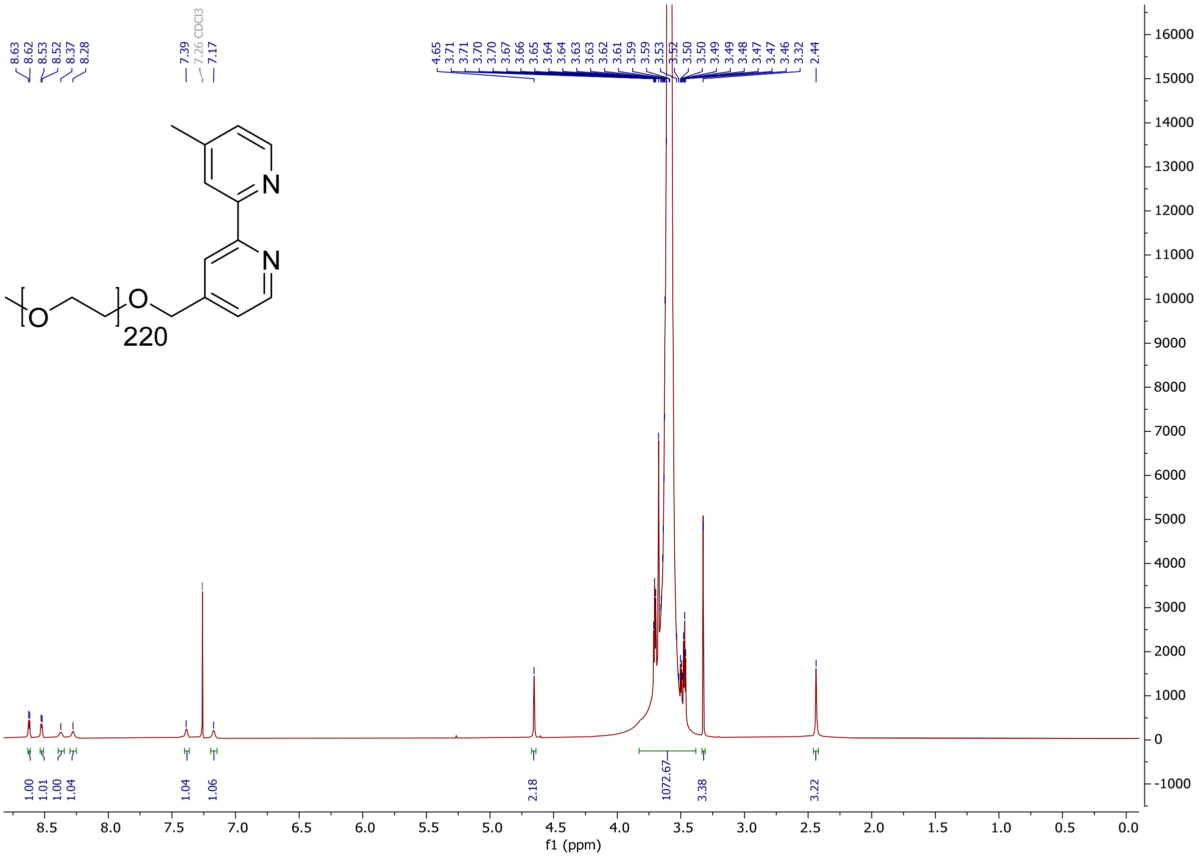

*Figure S 46: ^{1}H NMR (600 MHz, D_2O) of 4-methoxypolyethyleneglycolmethyl-4’-methyl-2,2’-bipyridine (**9**).*

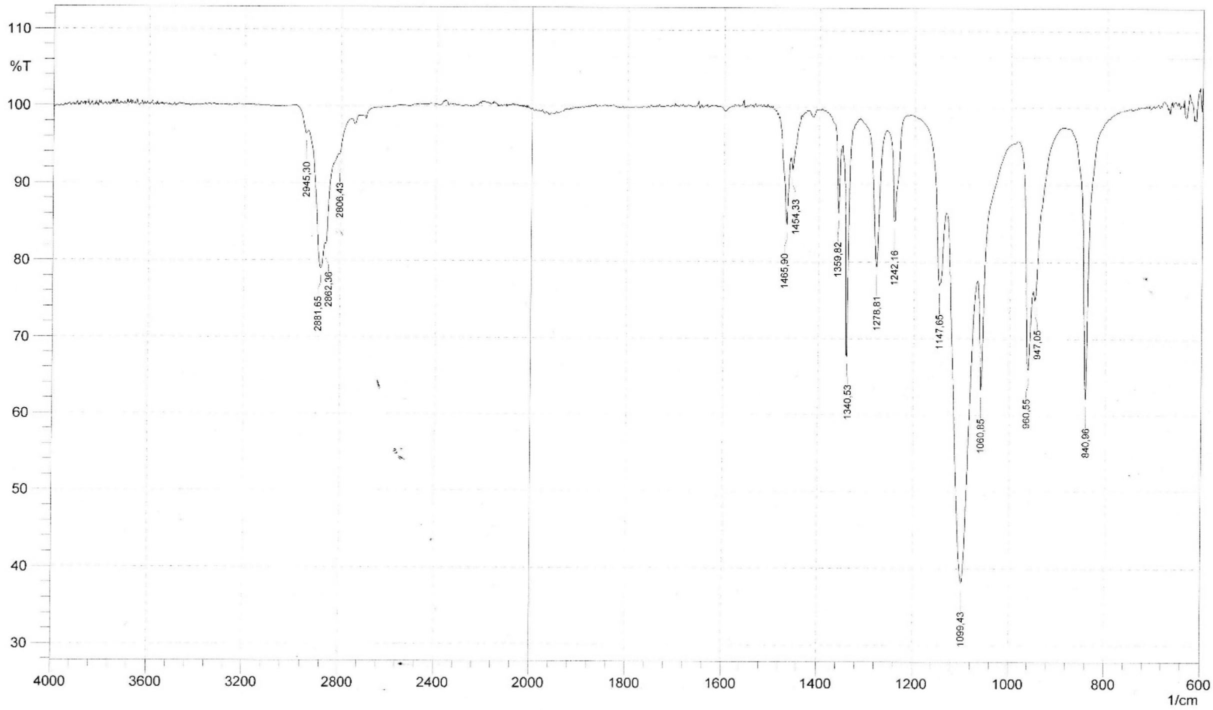

*Figure S 47: IR spectrum of 4-methoxypolyethyleneglycolmethyl-4’-methyl-2,2’-bipyridine (**9**).*

(4-Methoxypolyethyleneglycolmethyl-4'-methyl-2,2'-bibyridine)-dichloropalladium(II) (**10**)

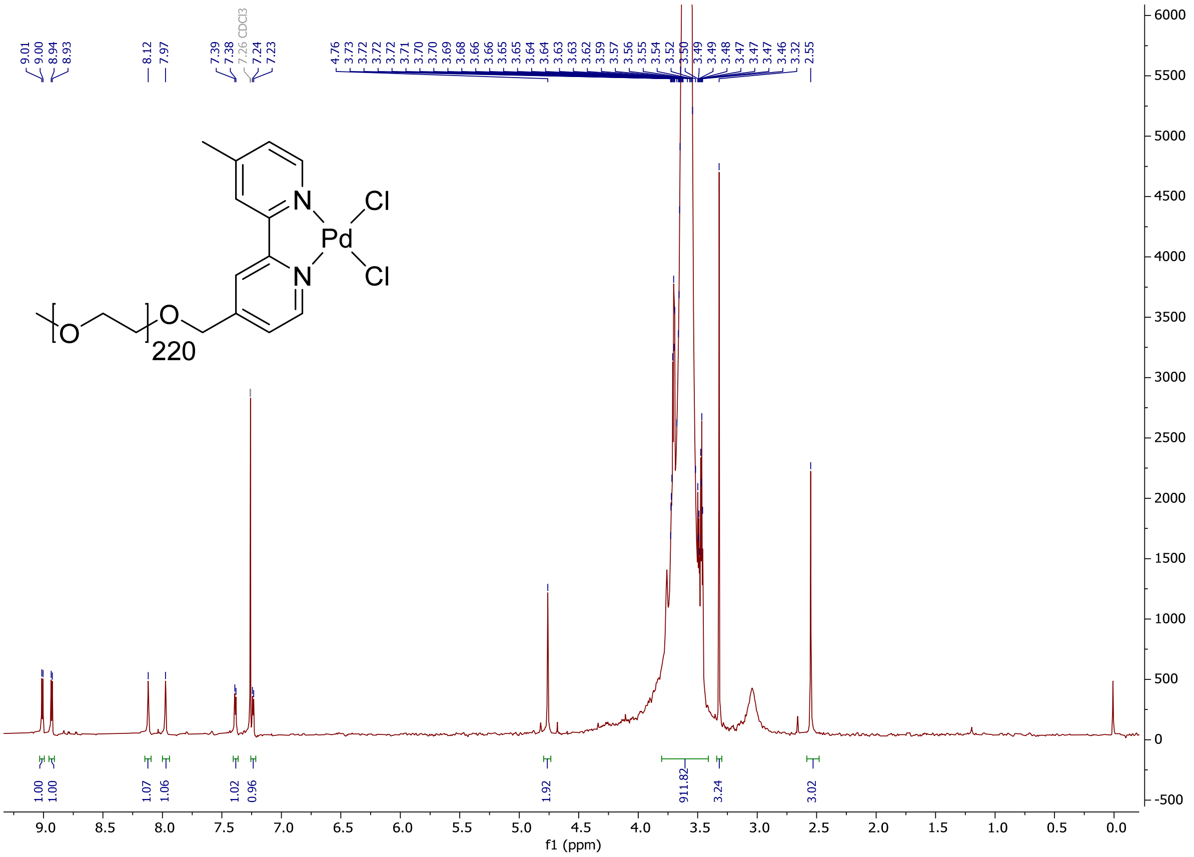

*Figure S 48: ^{1}H NMR (600 MHz, $CDCl_3$) of (4-methoxypolyethyleneglycolmethyl-4'-methyl-2,2'-bipyridine)-dichloropalladium(II) (**10**).*

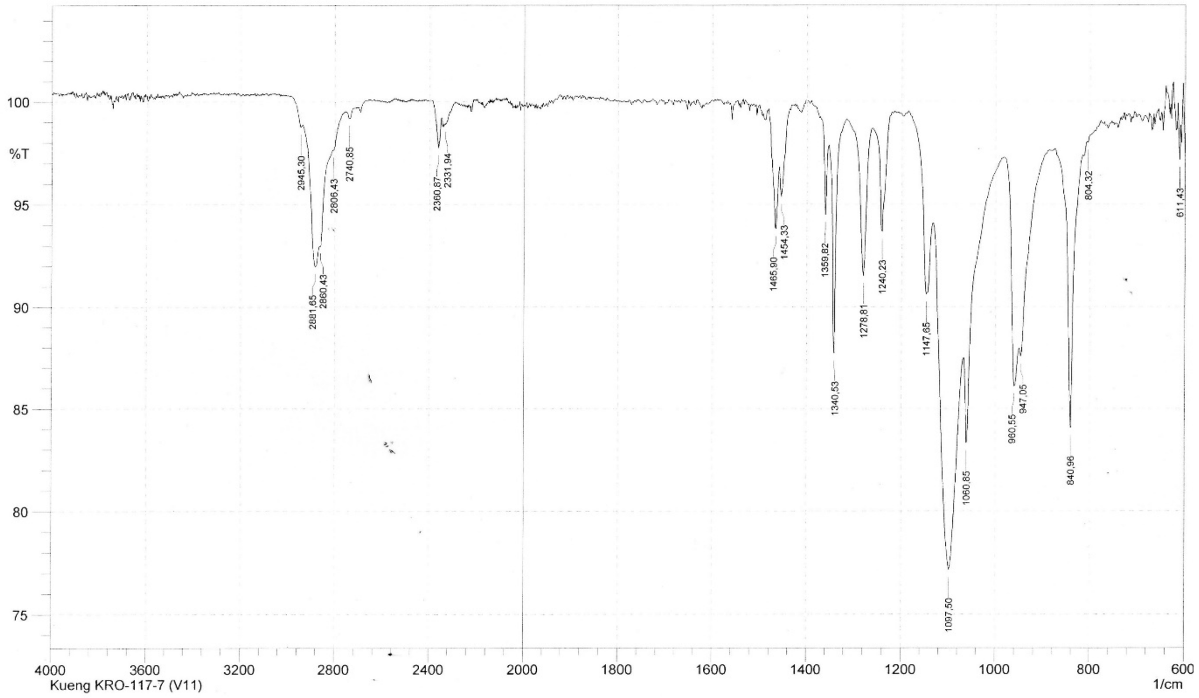

*Figure S 49: IR spectrum of (4-methoxypolyethyleneglycolmethyl-4'-methyl-2,2'-bipyridine)-dichloropalladium(II) (**10**).*

(4-Methoxypolyethyleneglycolmethyl-4'-methyl-2,2'-bipyridine)-dinitratopalladium(II) (**11**)

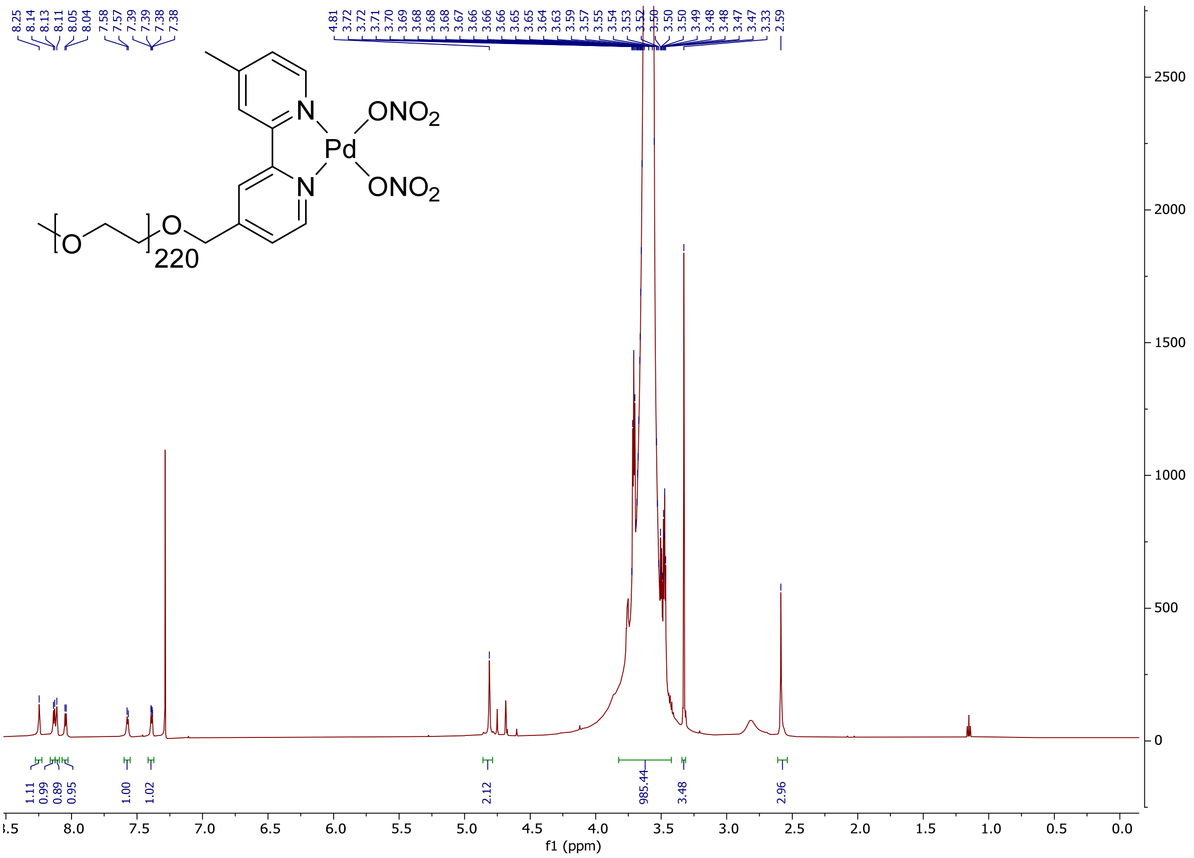

*Figure S 50: ^{1}H NMR (600 MHz, $CDCl_3$) of (4-methoxypolyethyleneglycolmethyl-4'-methyl-2,2'-bipyridine)-dinitratopalladium(II) (**11**).*

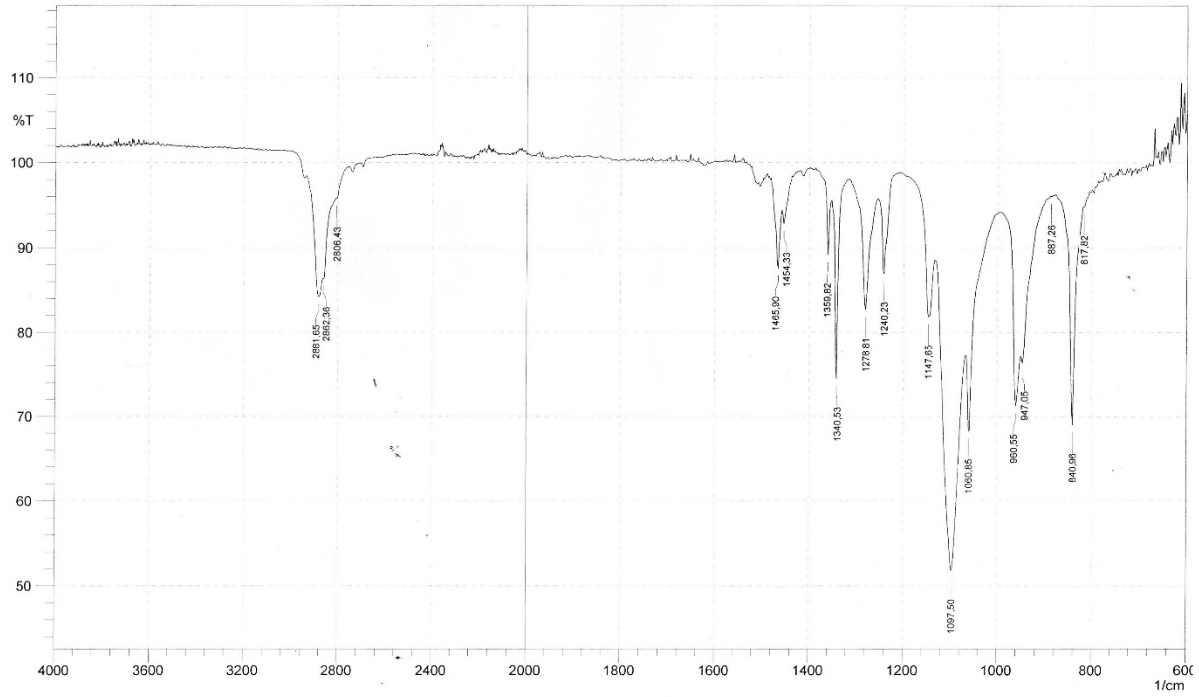

*Figure S 51: IR spectrum of (4-methoxypolyethyleneglycolmethyl-4'-methyl-2,2'-bipyridine)-dinitratopalladium(II) (**11**).*

Cage 1a

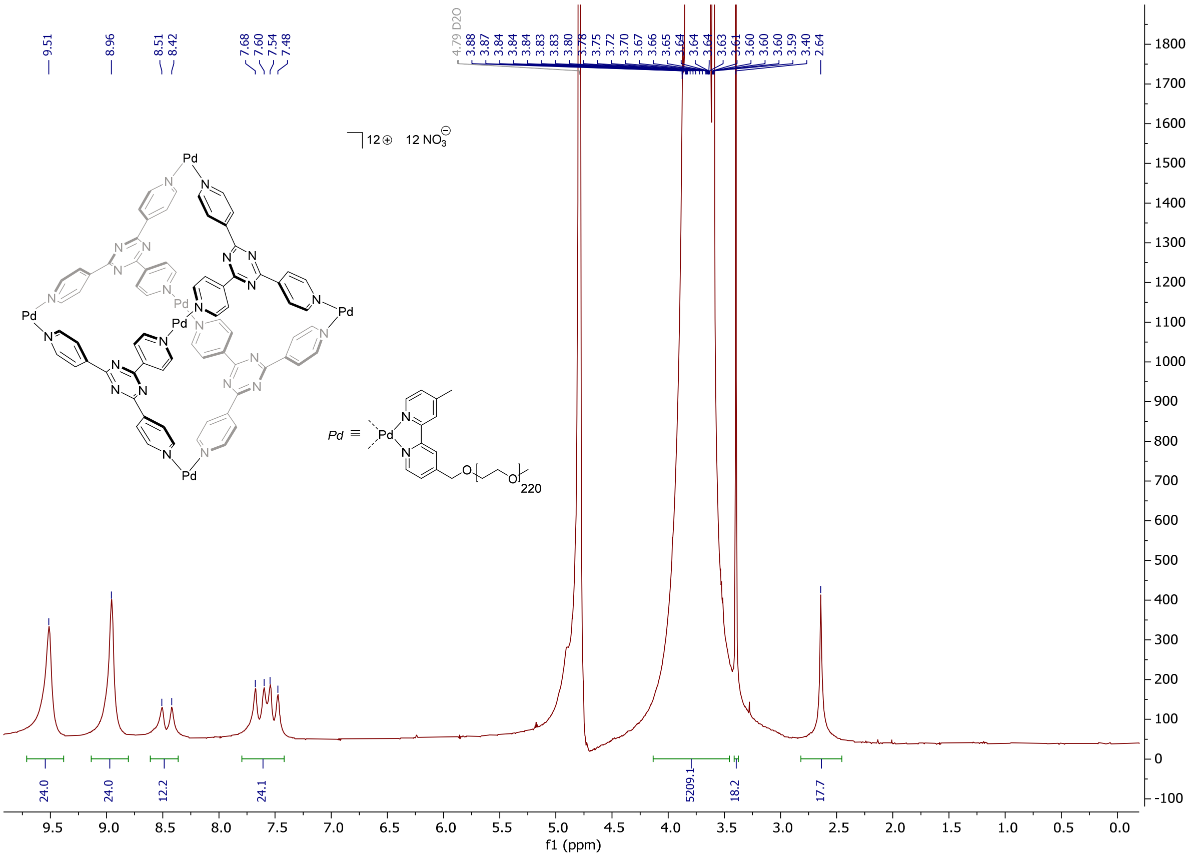

*Figure S 52: ^{1}H NMR (600 MHz, D_2O) of cage **1a**.*

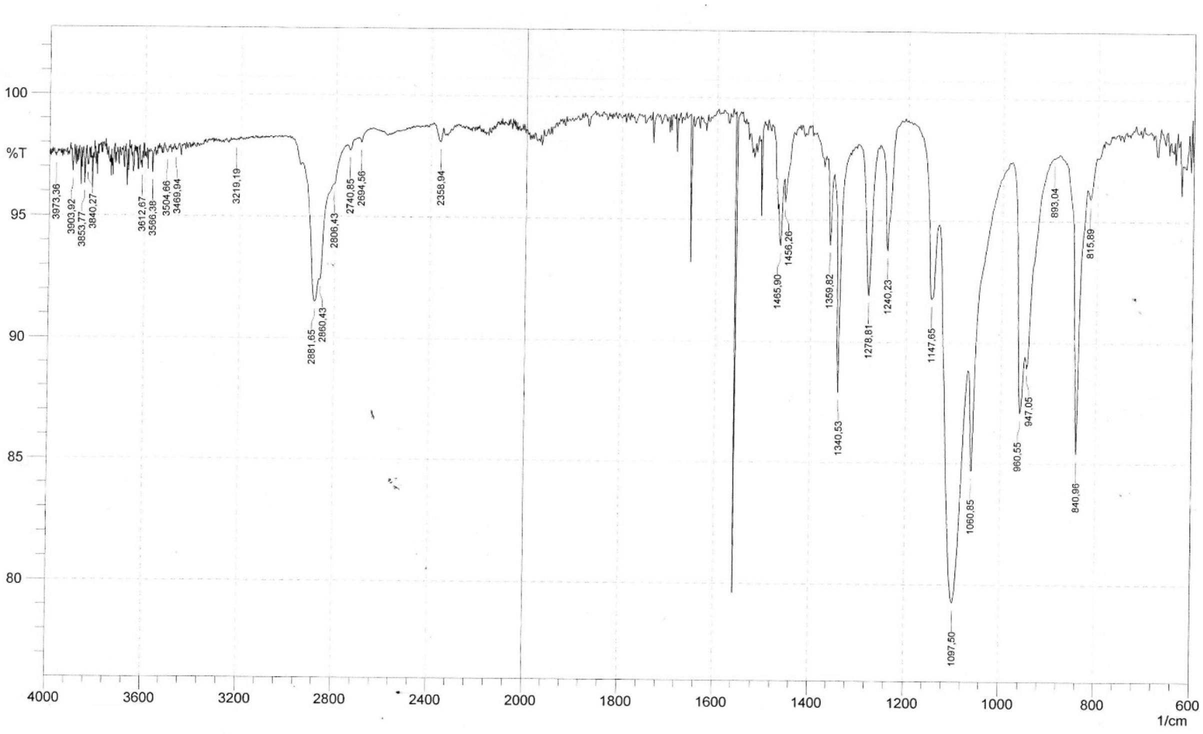

*Figure S 53: IR spectrum of cage **1a**.*

4-Methoxypolyethyleneglycolmethyl-4’-methyl-2,2’-bipyridine (12)

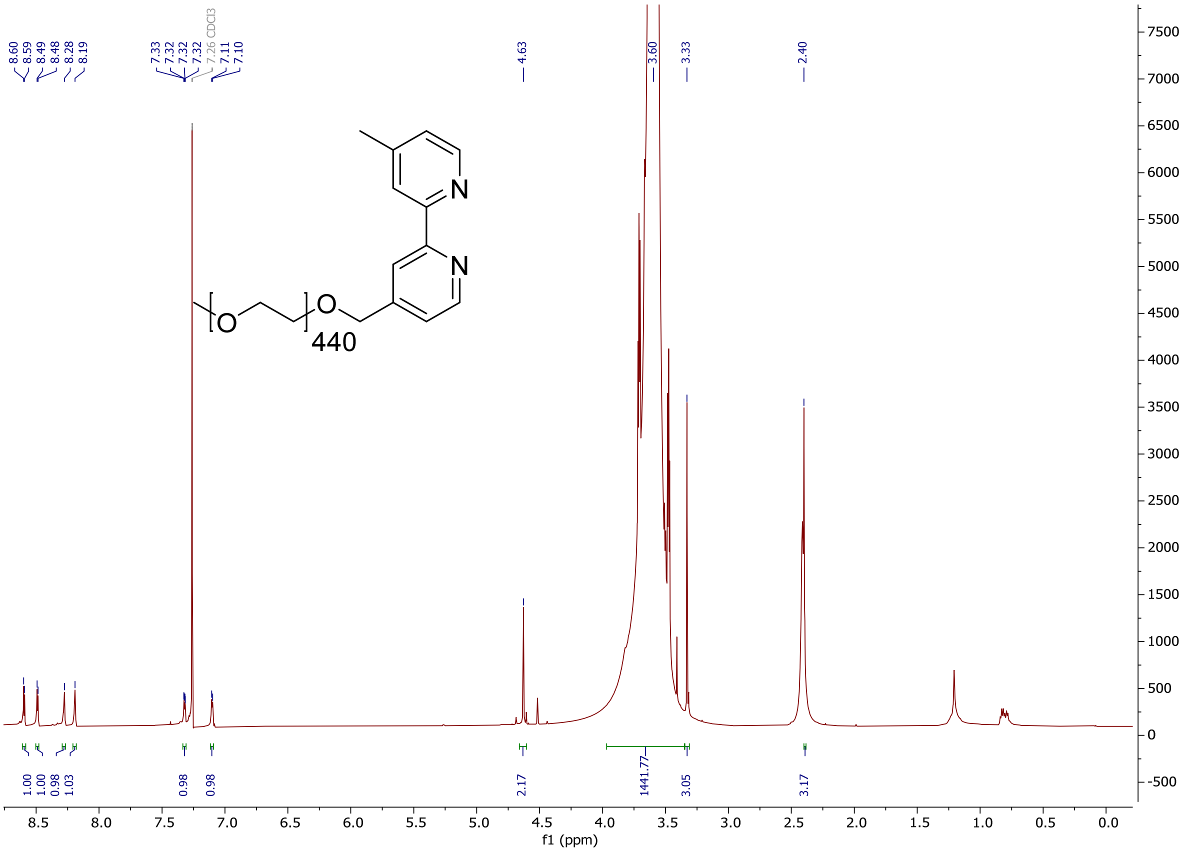

*Figure S 54: ^{1}H NMR (600 MHz, $CDCl_3$) of 4-methoxypolyethyleneglycolmethyl-4’-methyl-2,2’-bipyridine (**12**).*

(4-Methoxypolyethyleneglycolmethyl-4‘-methyl-2,2‘-bibyridine)-dichloropalladium(II) (13)

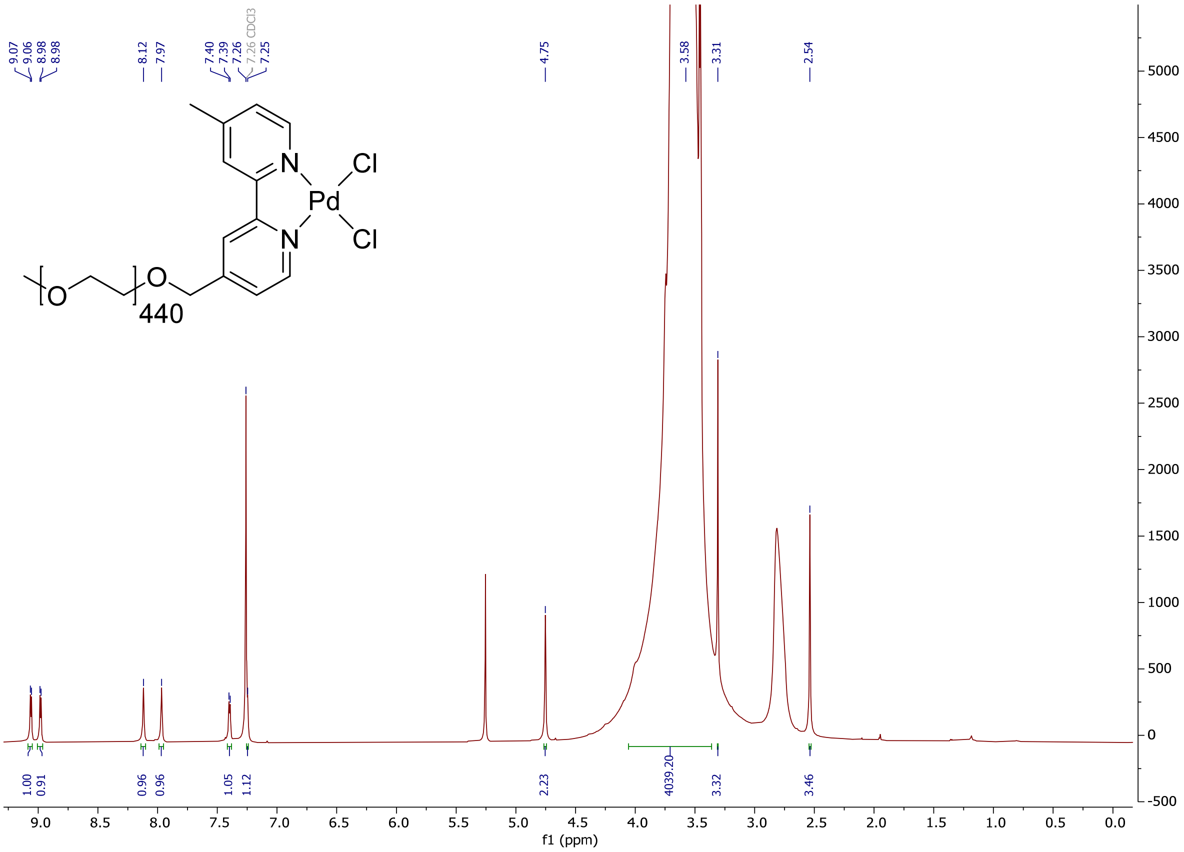

*Figure S 55: ^1H NMR (600 MHz, $CDCl_3$) of (4-methoxypolyethyleneglycolmethyl-4’-methyl-2,2’-bipyridine)-dichloropalladium(II) (**13**).*

(4-Methoxypolyethyleneglycolmethyl-4’-methyl-2,2’-bipyridine)-dinitratopalladium(II) (**14**)

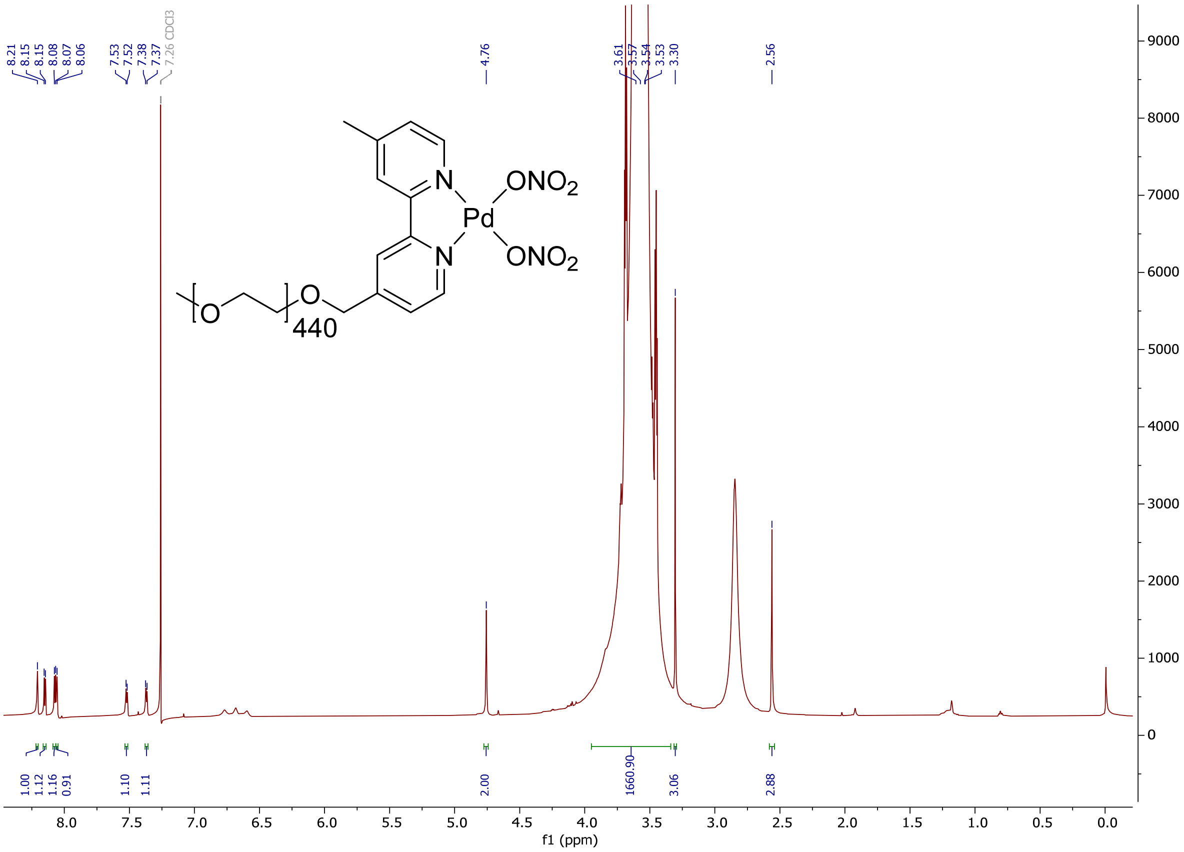

*Figure S 56: ¹H NMR (600 MHz, $CDCl_3$) of (4-methoxypolyethyleneglycolmethyl-4’-methyl-2,2’-bipyridine)-dinitratopalladium(II) (**14**).*

Cage **1b**

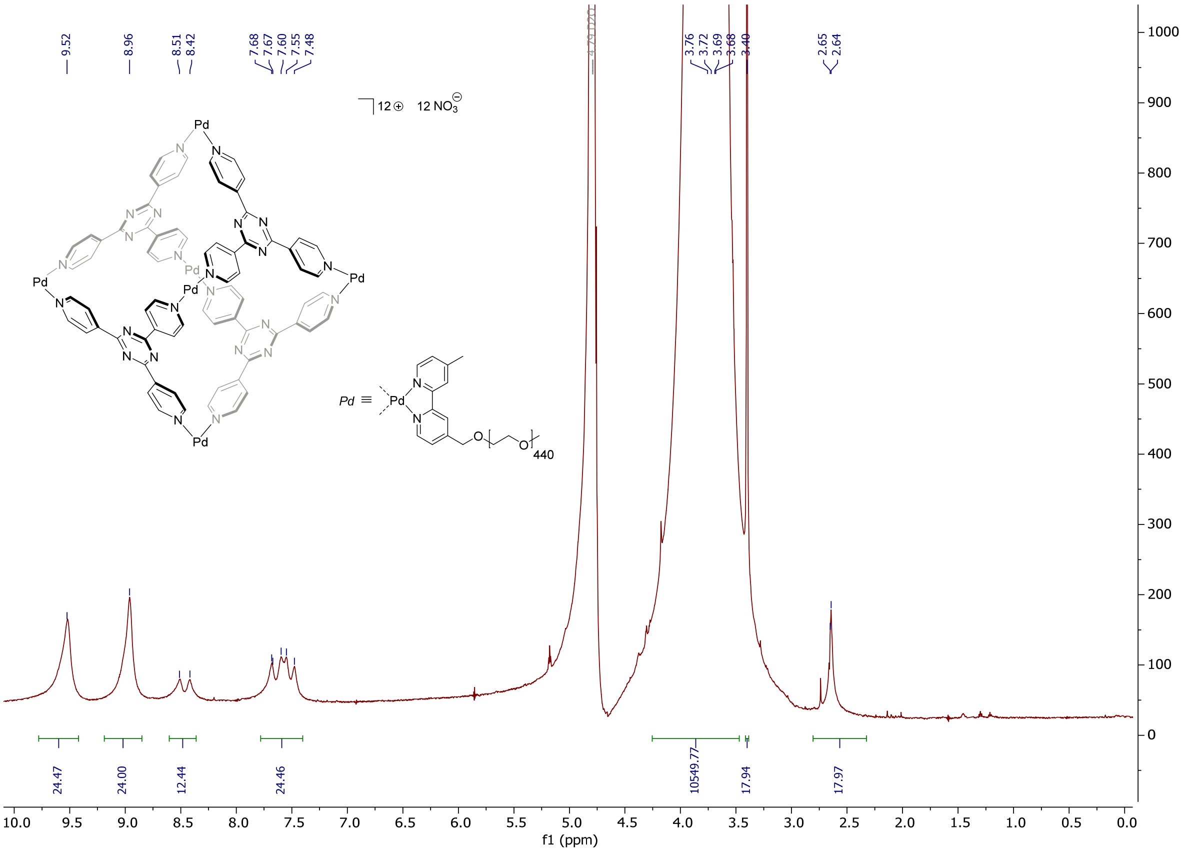

*Figure S 57: ^{1}H NMR (600 MHz, D_2O) of cage **1b**.*

Spectra encapsulation and release studies

Cage **1a**

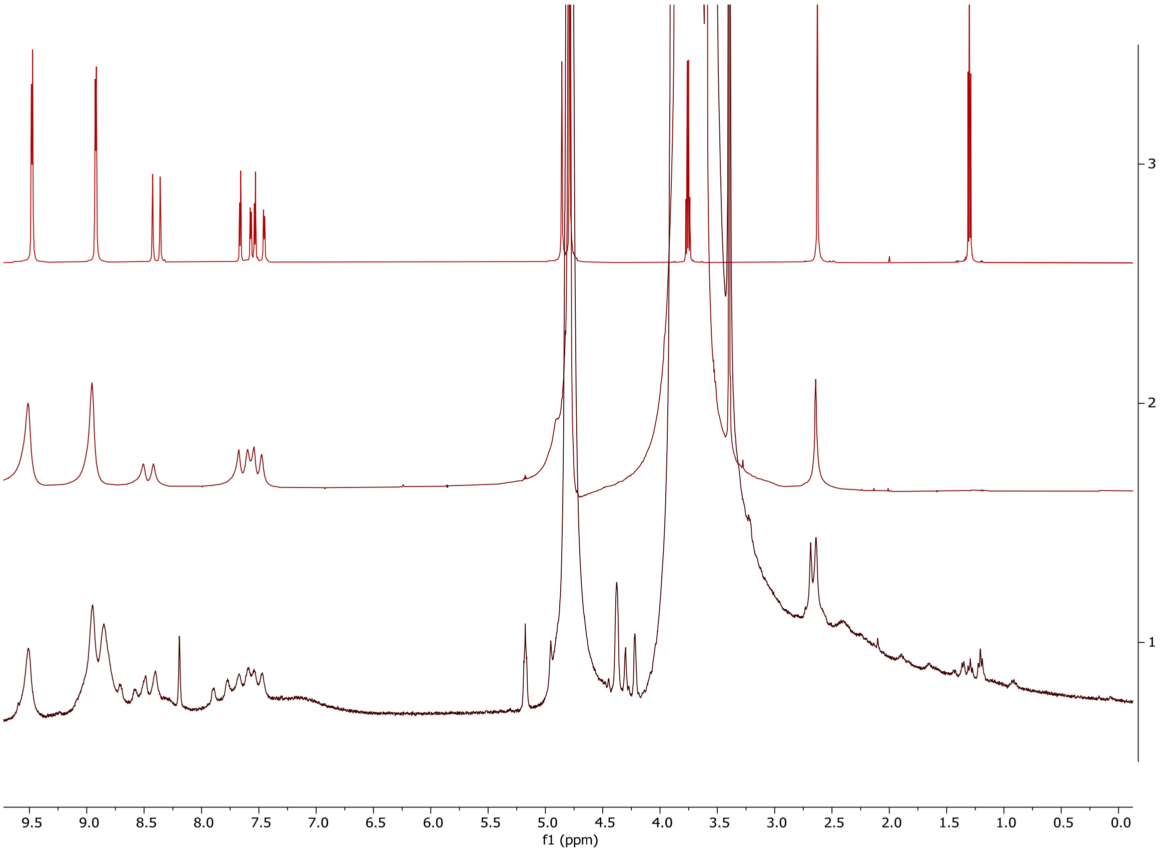

*Figure S 58: ^{1}H NMR: Cage **2** (top), cage **1a** before sonication (mid), cage **1a** after sonication (bottom).*

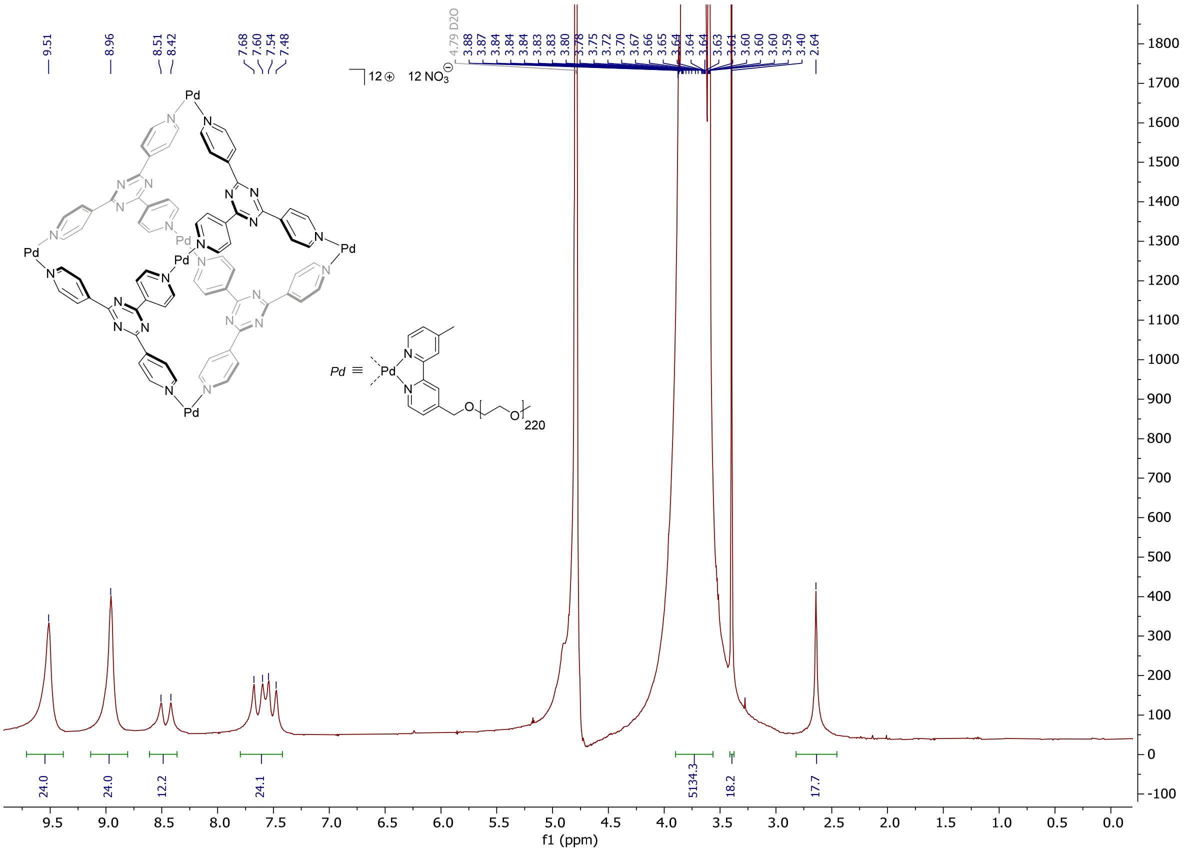

*Figure S 59: ^{1}H NMR (600 MHz, D_2O) of cage **1a** before sonication.*

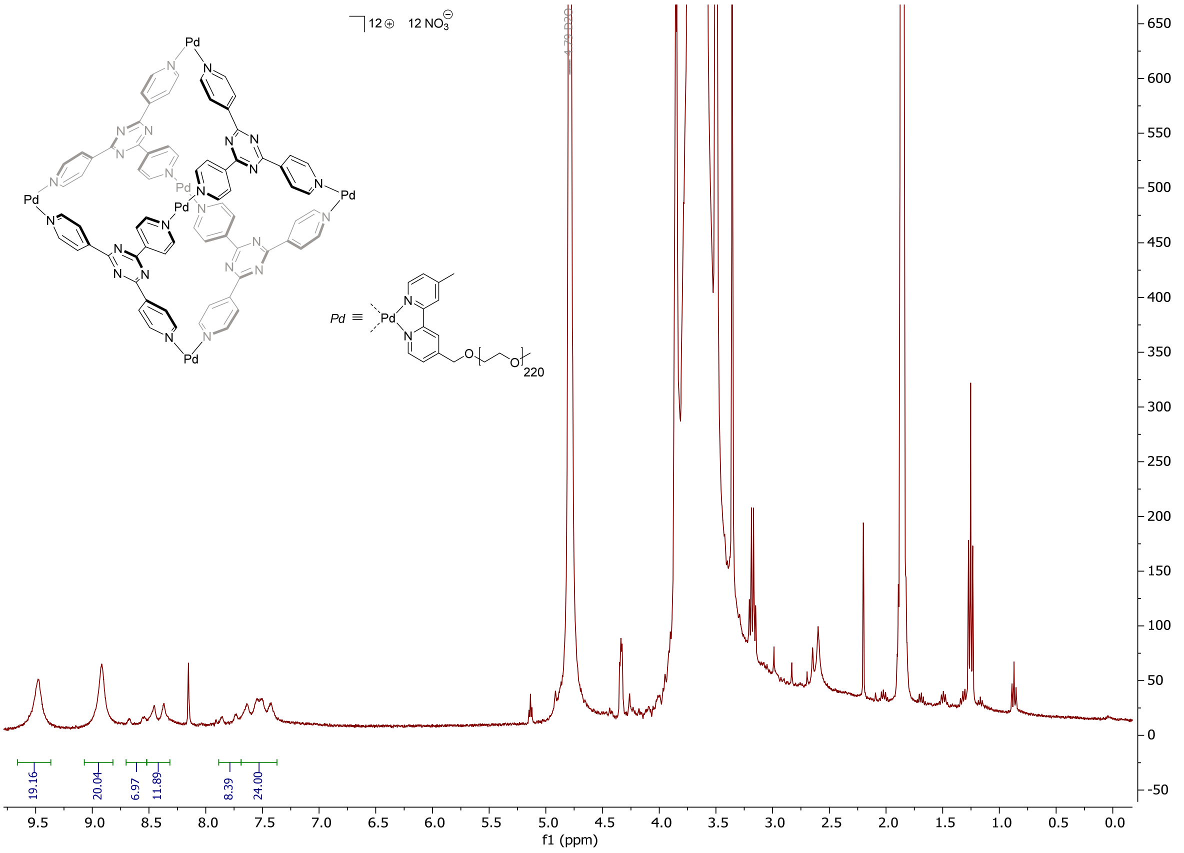

*Figure S 60: ^{1}H NMR (400 MHz, D_2O) of cage **1a** after 1 h sonication.*

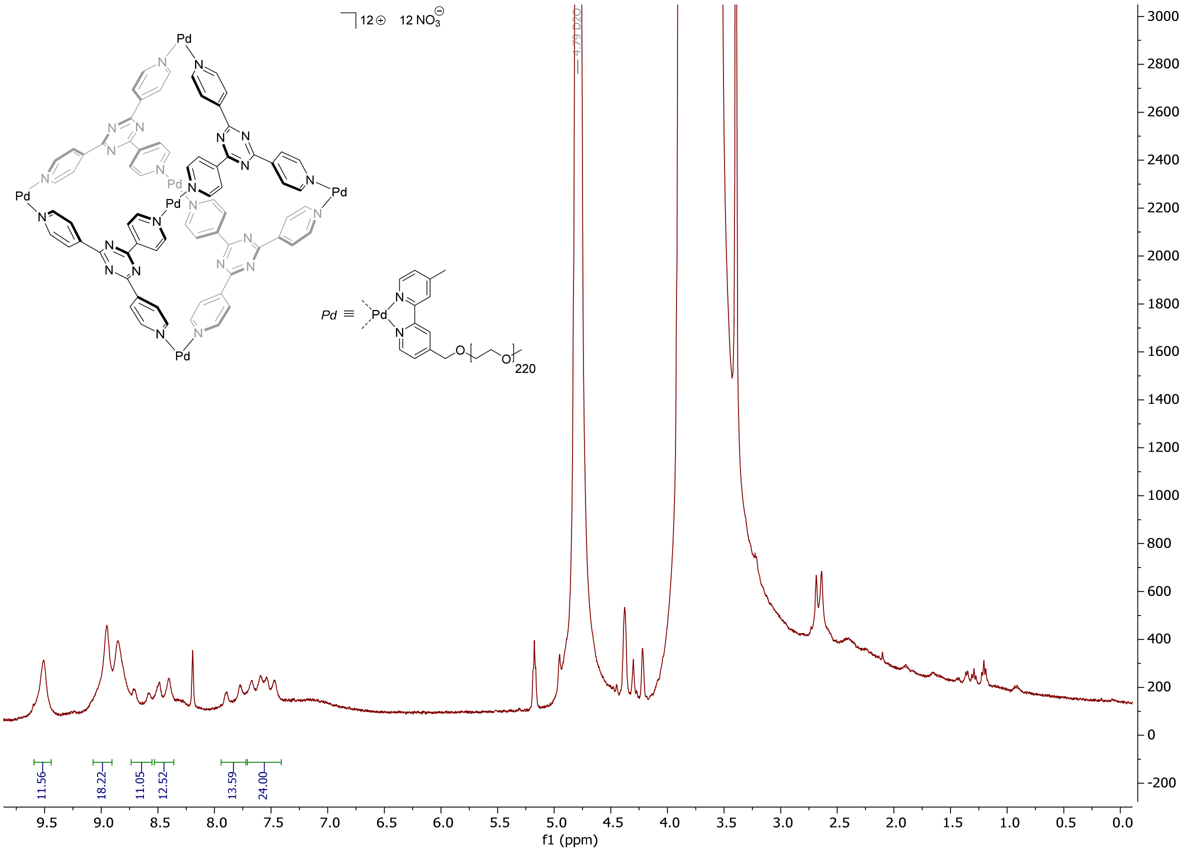

*Figure S 61: ^1H NMR (400 MHz, D_2O) of cage **1a** after 3 h sonication.*

Cage 1b

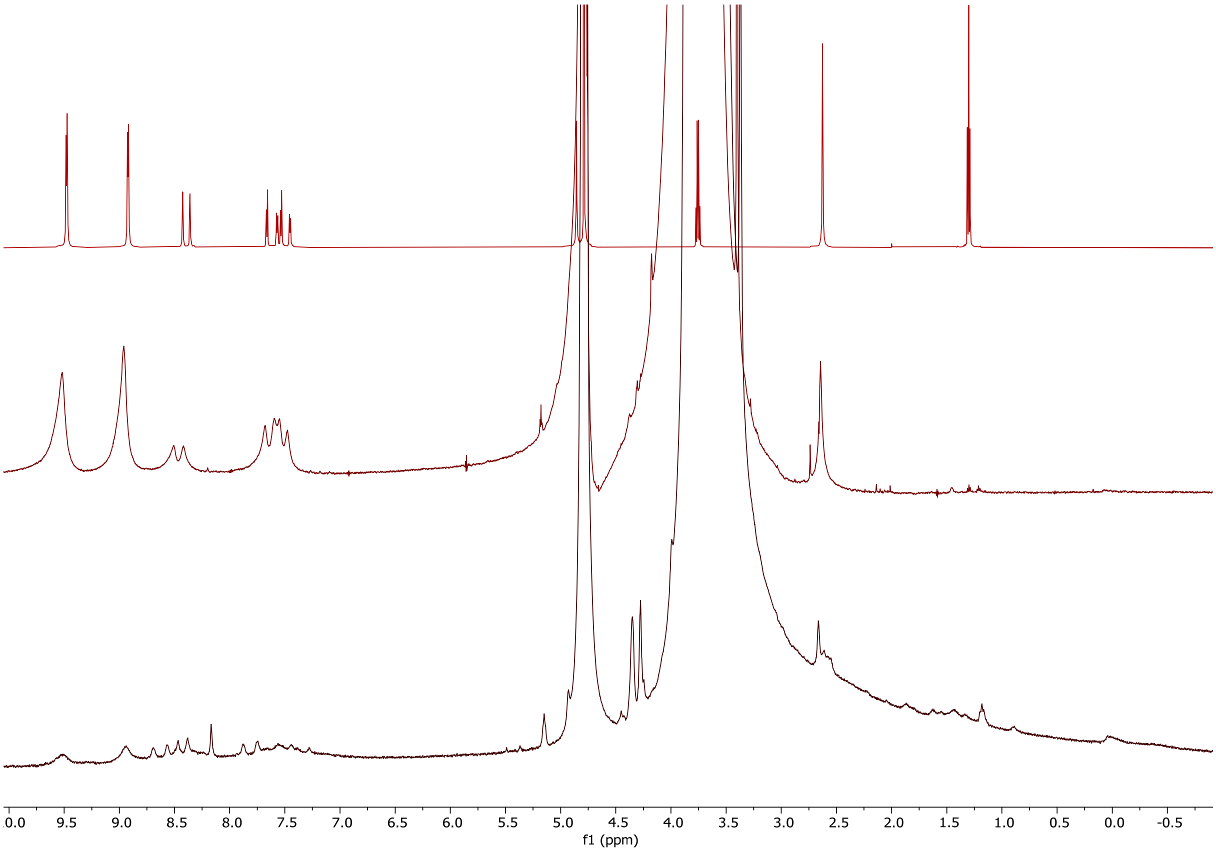

*Figure S 62: ^{1}H NMR: Cage **2** (top), cage **1b** before sonication (mid), cage **1b** after sonication (bottom).*

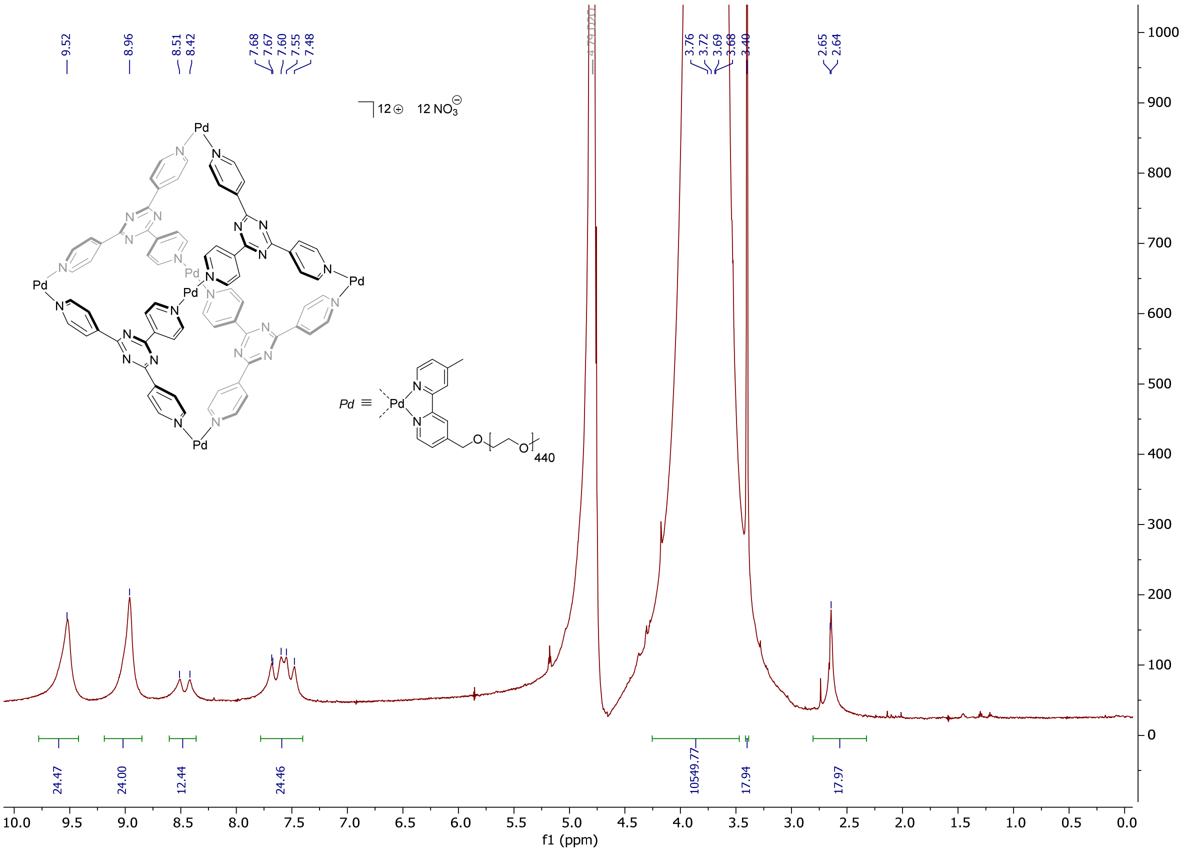

*Figure S 63: ^{1}H NMR (600 MHz, D_2O) of cage **1b** before sonication.*

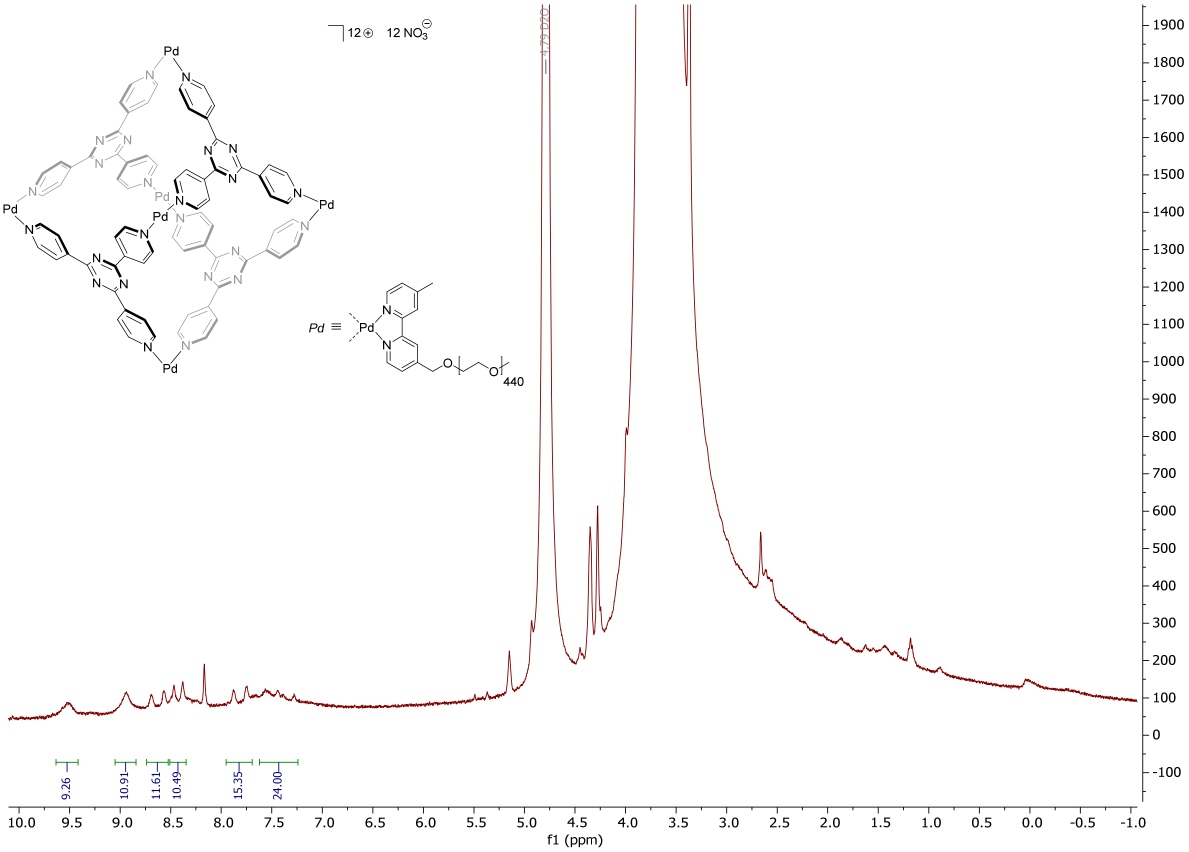

*Figure S 64: ^{1}H NMR (400 MHz, D_2O) of cage **1b** after 1 h sonication.*

Comparison of spectra of cage **2•(progesterone)** & cage **1a•(progesterone)** before sonication and cage **1a•(progesterone)** after sonication

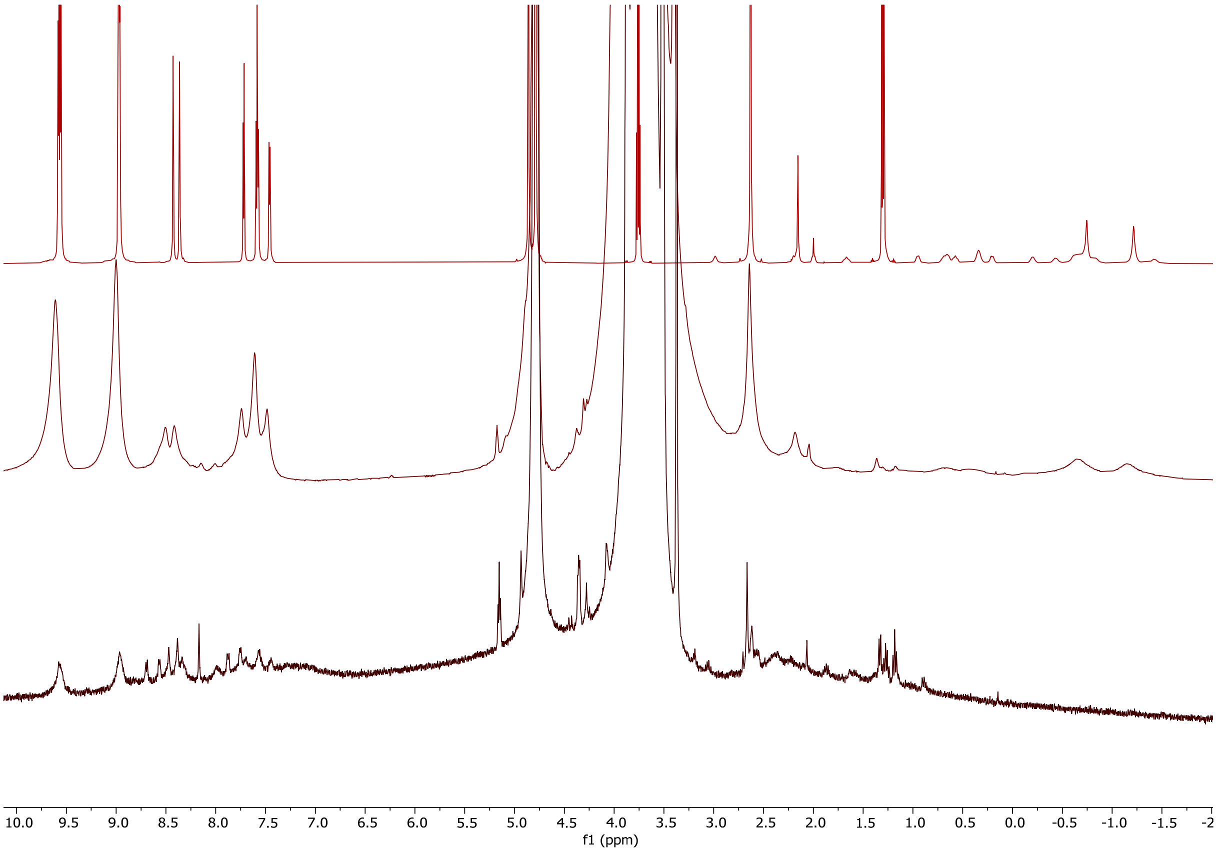

*Figure S 65: ¹H NMR: Cage **2•(progesterone)** (top), cage **1a•(progesterone)** before sonication (mid), cage **1a•(progesterone)** after sonication (bottom).*

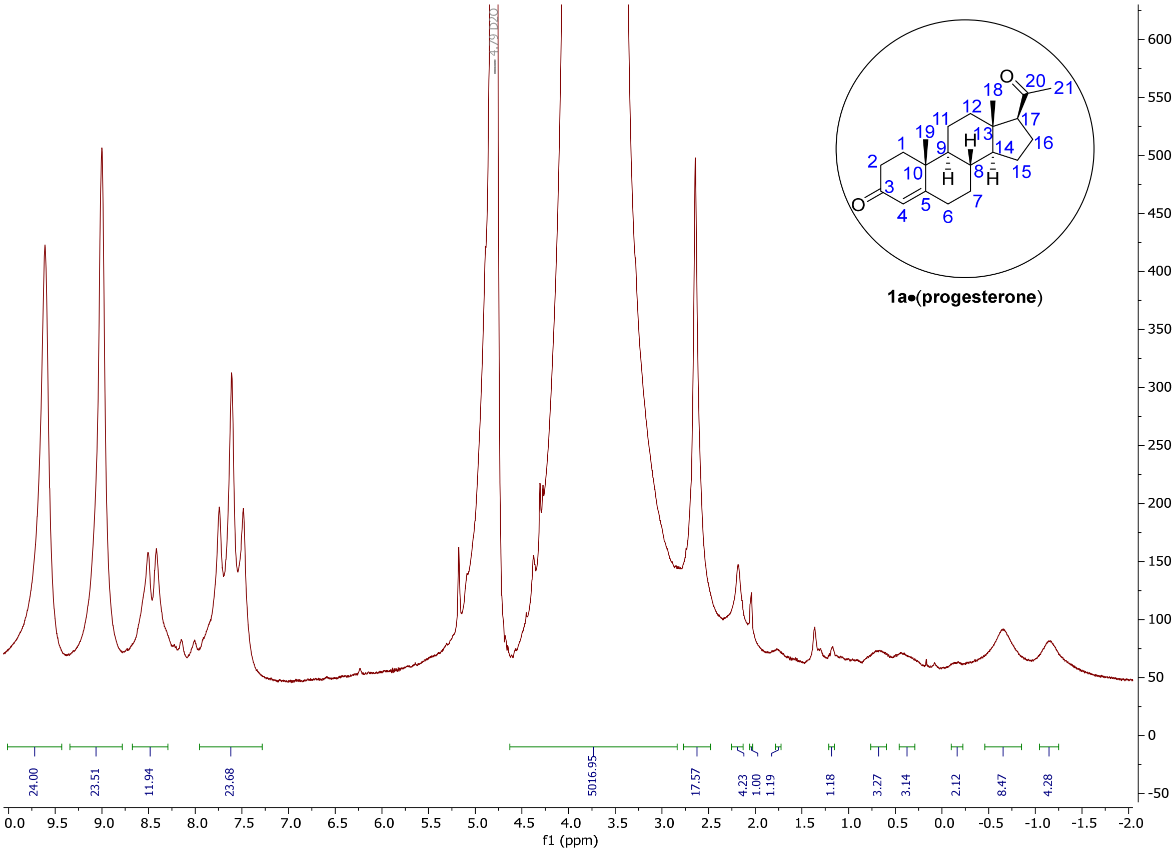

*Figure S 66: ^{1}H NMR (600 MHz, D_2O) of **1a•(progesterone)** before sonication.*

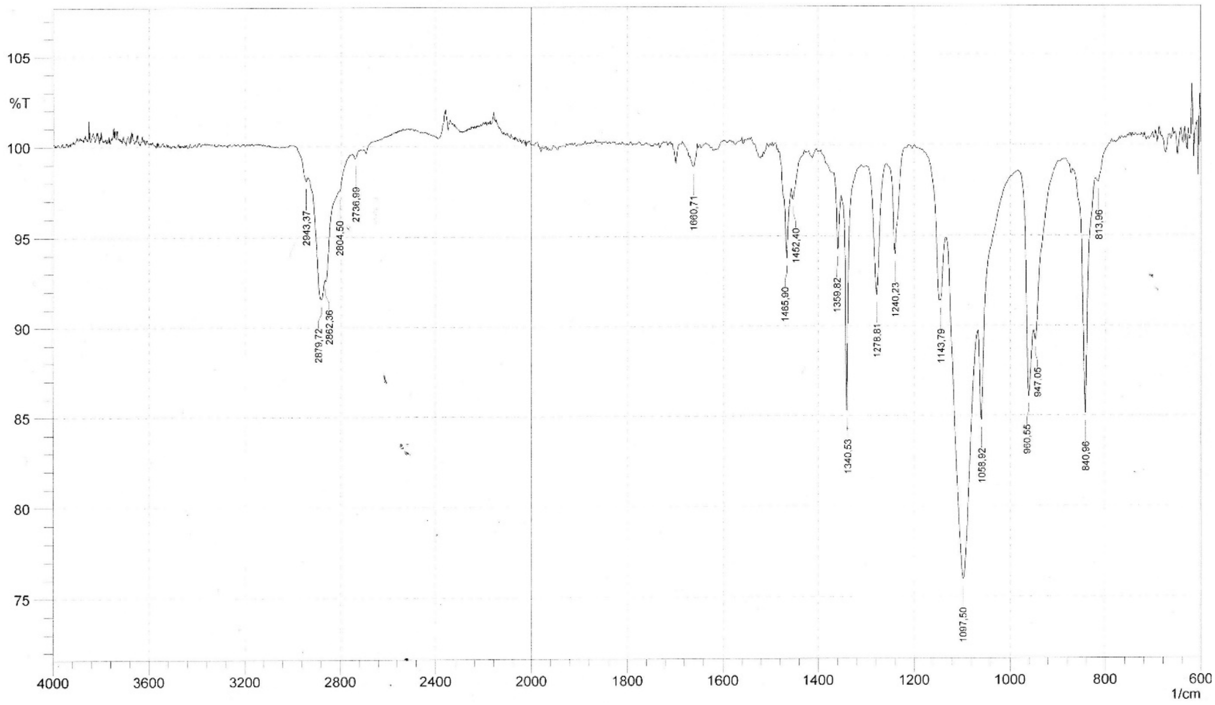

*Figure S 67: IR spectrum **1a•(progesterone)** before sonication.*

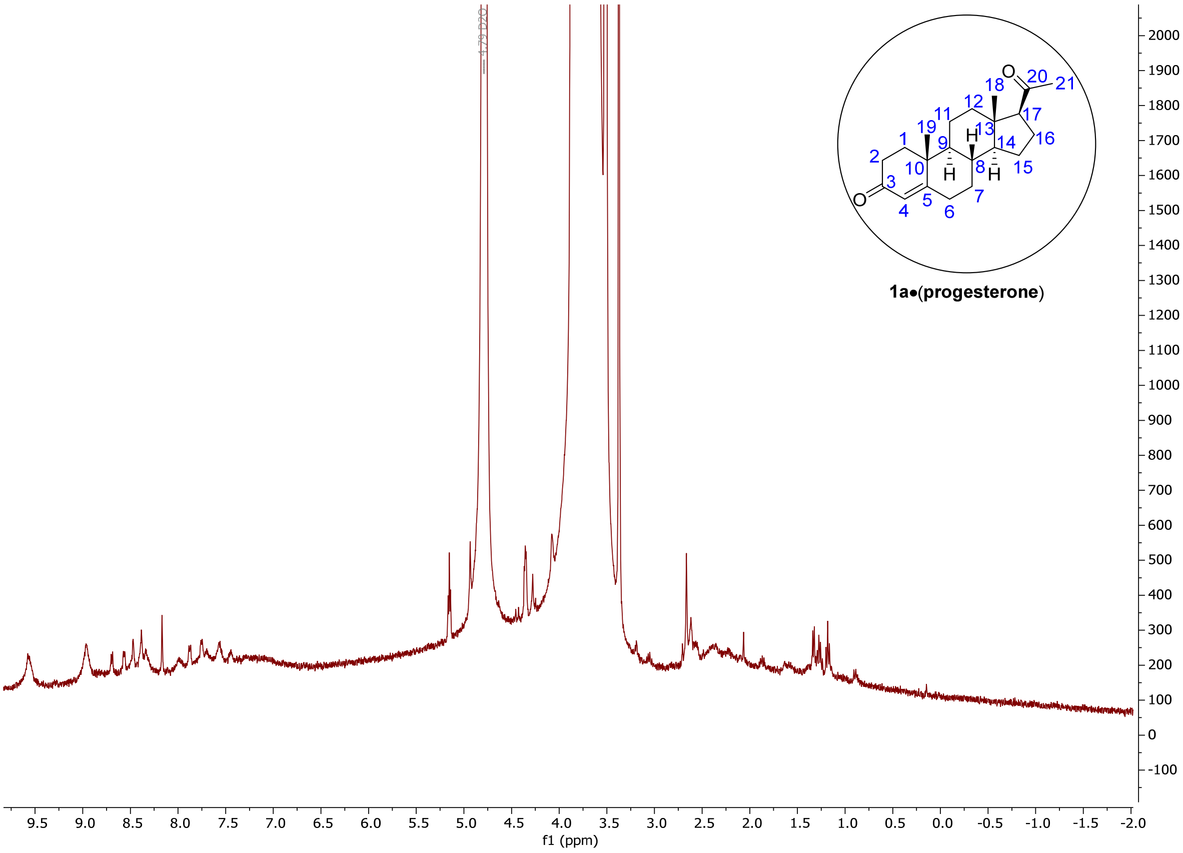

*Figure S 68: ^{1}H NMR (400 MHz, D_2O) of **1a•(progesterone)** after sonication.*

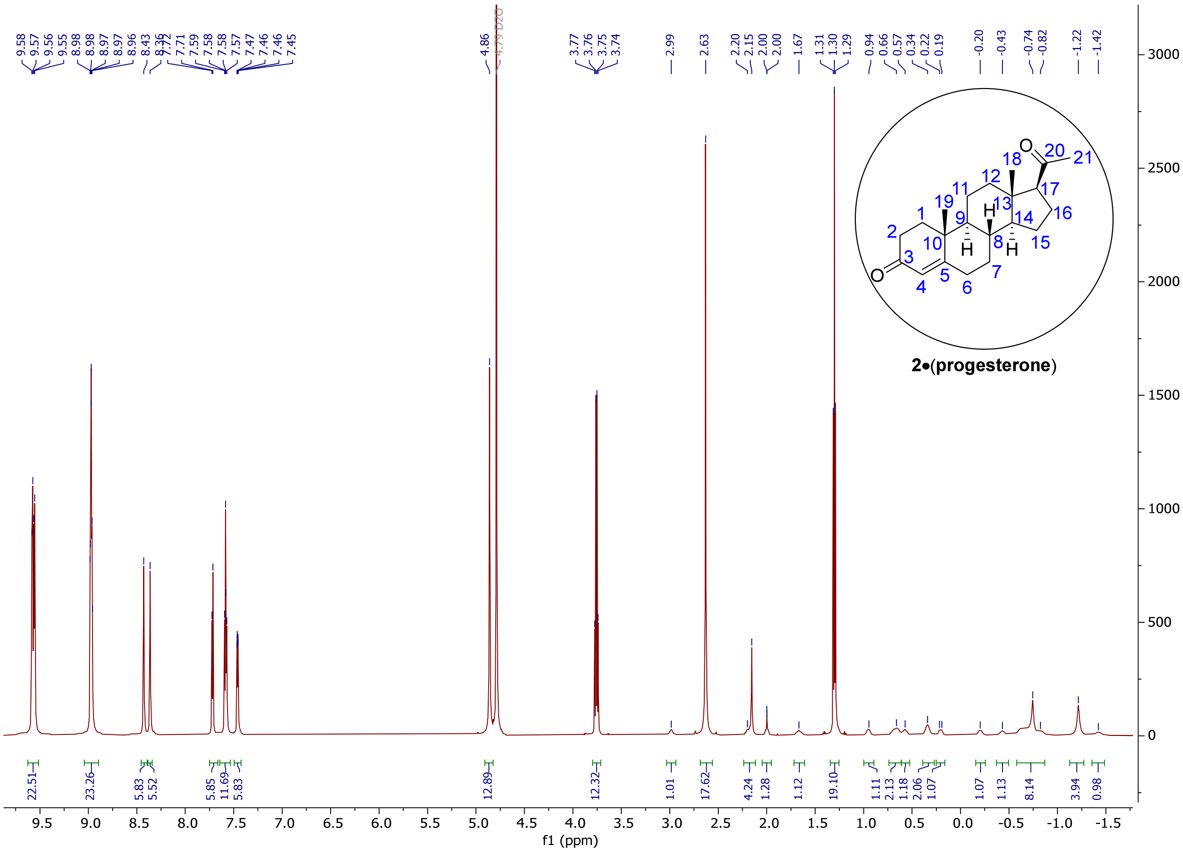

*Figure S 69: ^{1}H NMR (600 MHz, D_2O) of **2•(progesterone)**.*

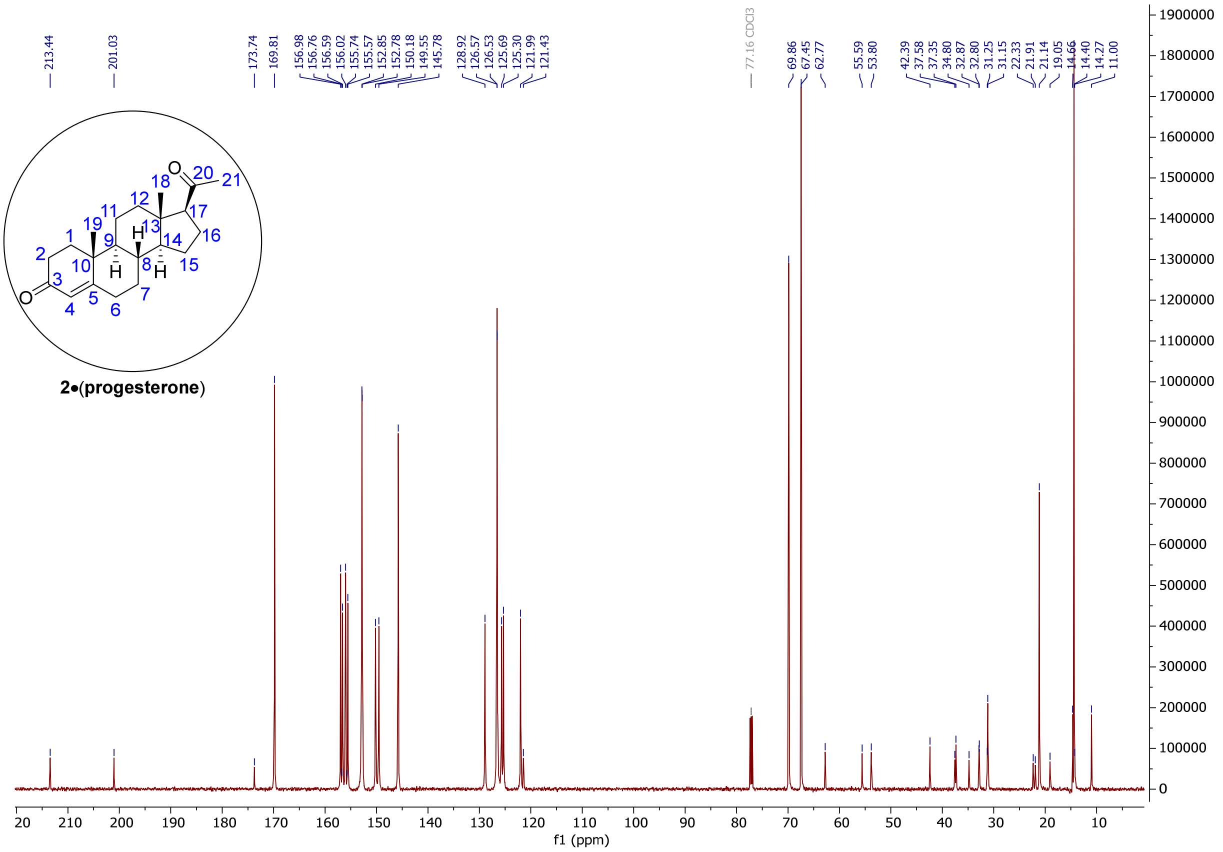

*Figure S 70: $^{13}C\{^{1}H\}$ NMR (151 MHz, D_2O) of **2•(progesterone)**.*

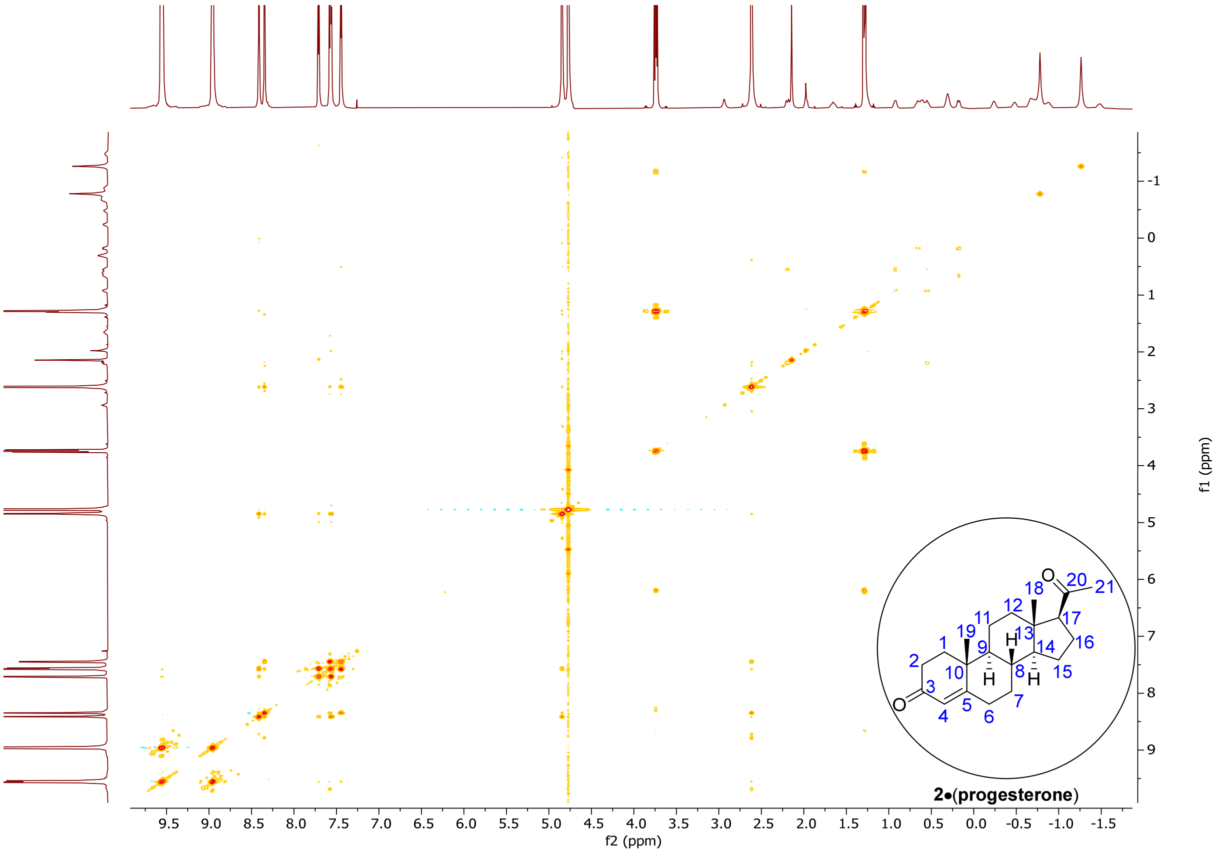

*Figure S 71: ^{1}H-^{1}H COSY NMR (600 MHz, D_2O) of **2•(progesterone)**.*

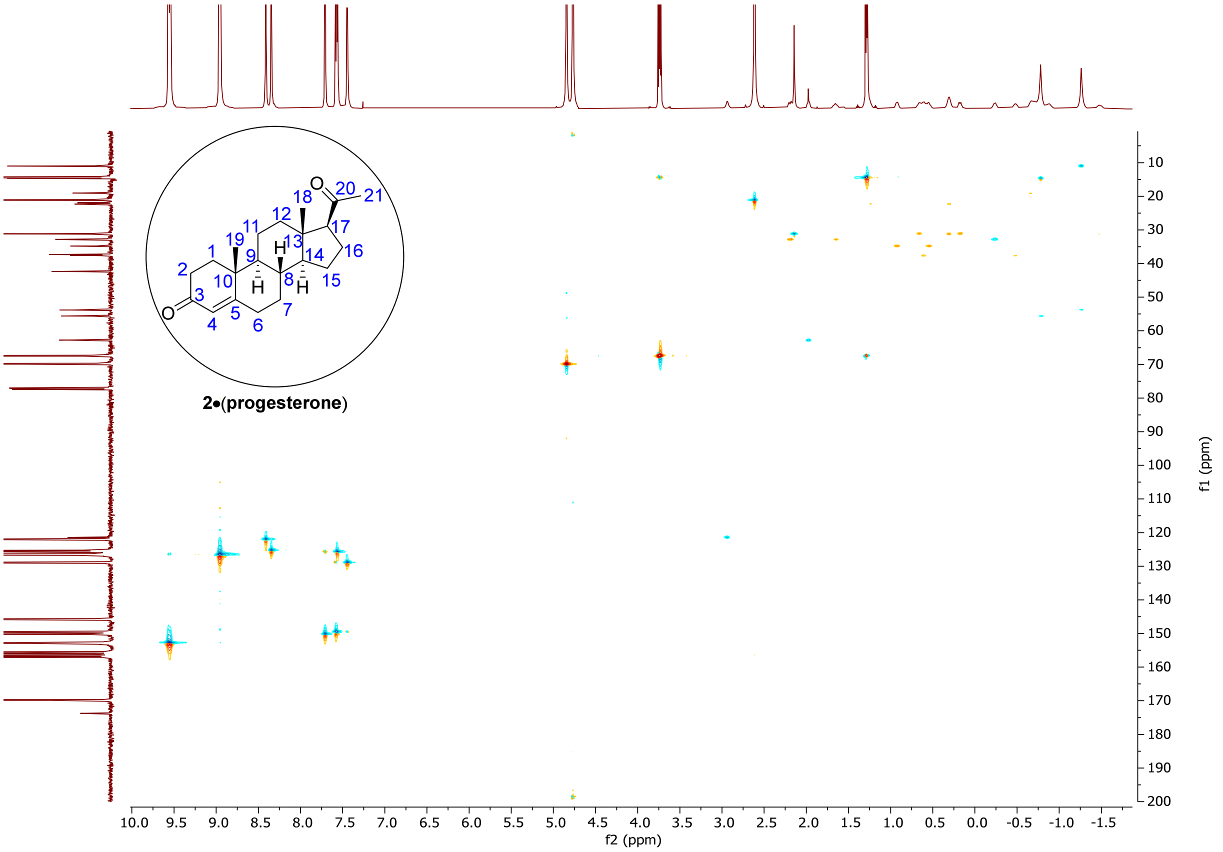

*Figure S 72: ^{1}H-{^{13}C} HSQC NMR (600 MHz, D_2O) of **2•(progesterone)**.*

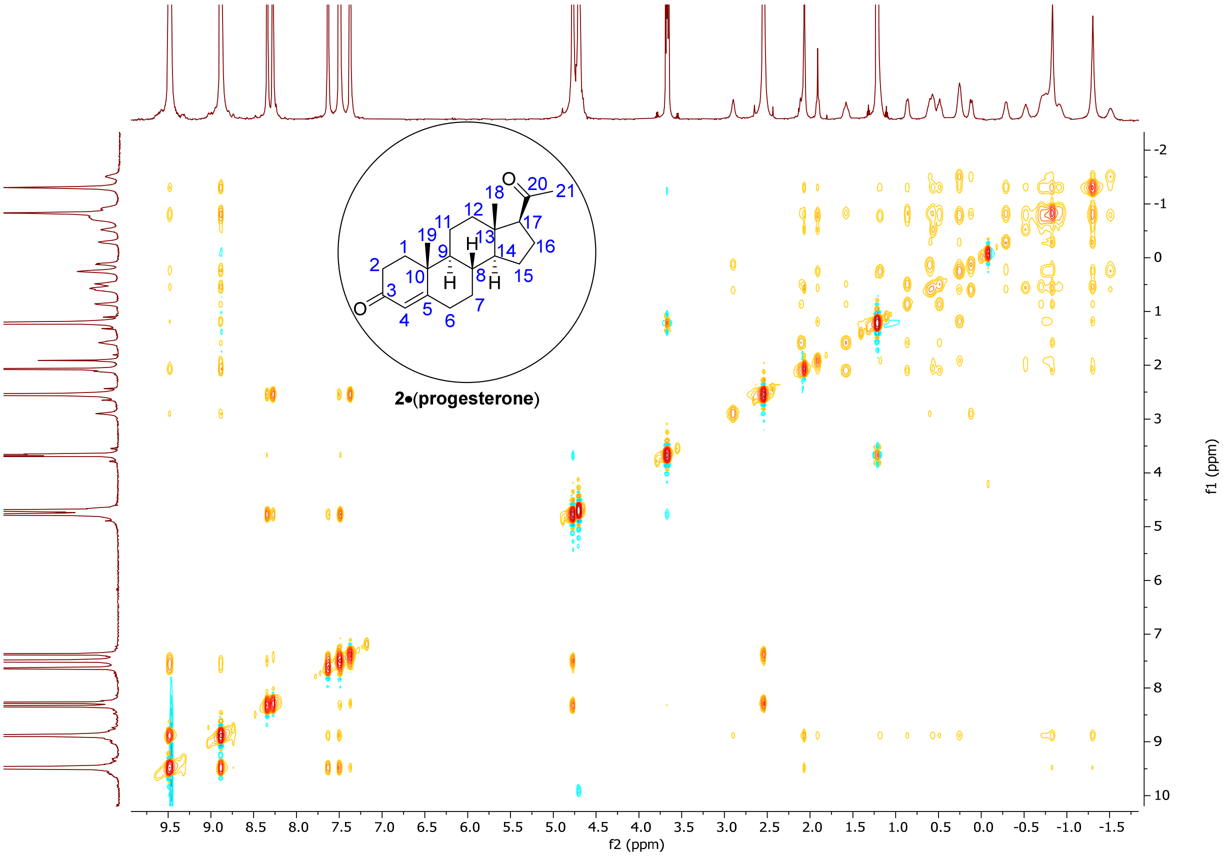

*Figure S 73: ^{1}H NOESY NMR (600 MHz, D_2O) of **2•(progesterone)**.*

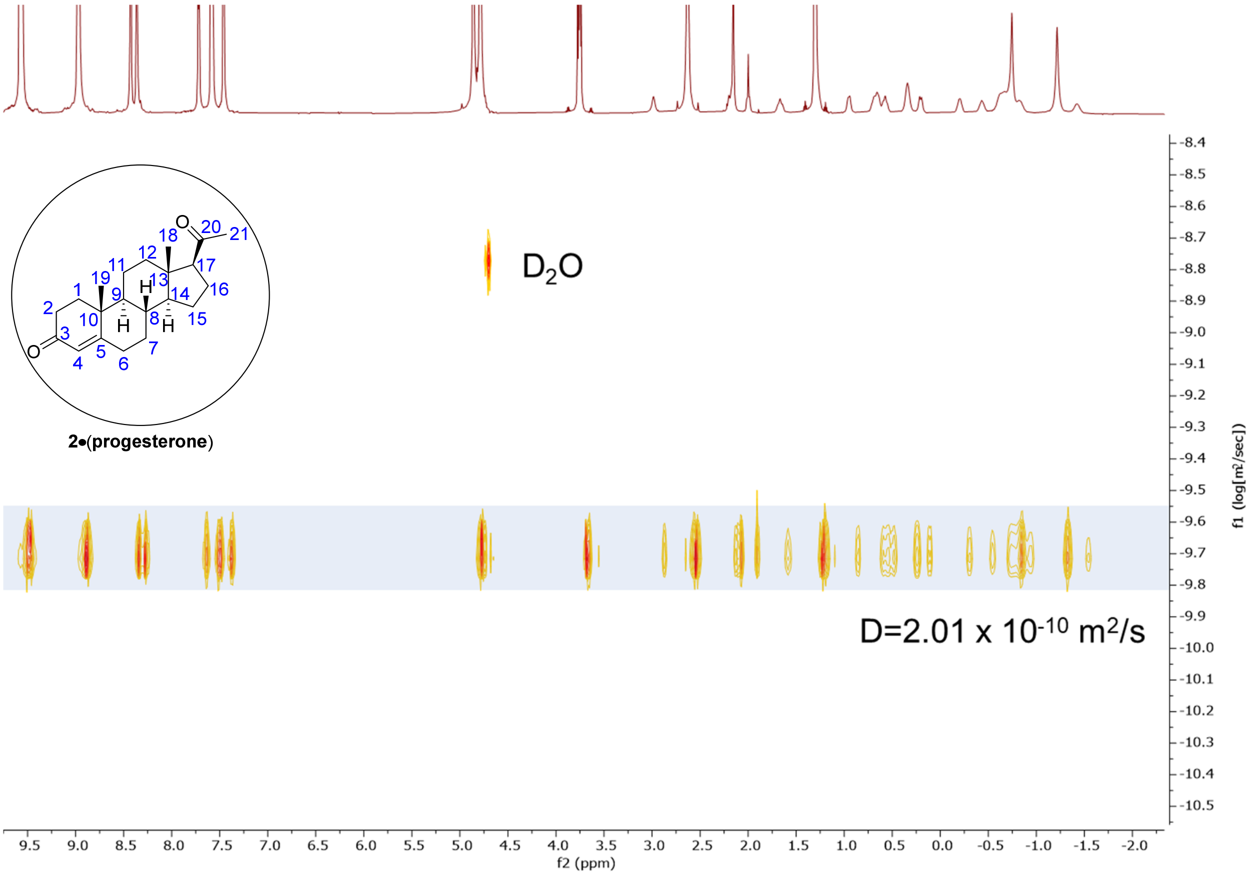

*Figure S 74: DOSY NMR (600 MHz, D$_2$O) of **2•(progesterone)**.*

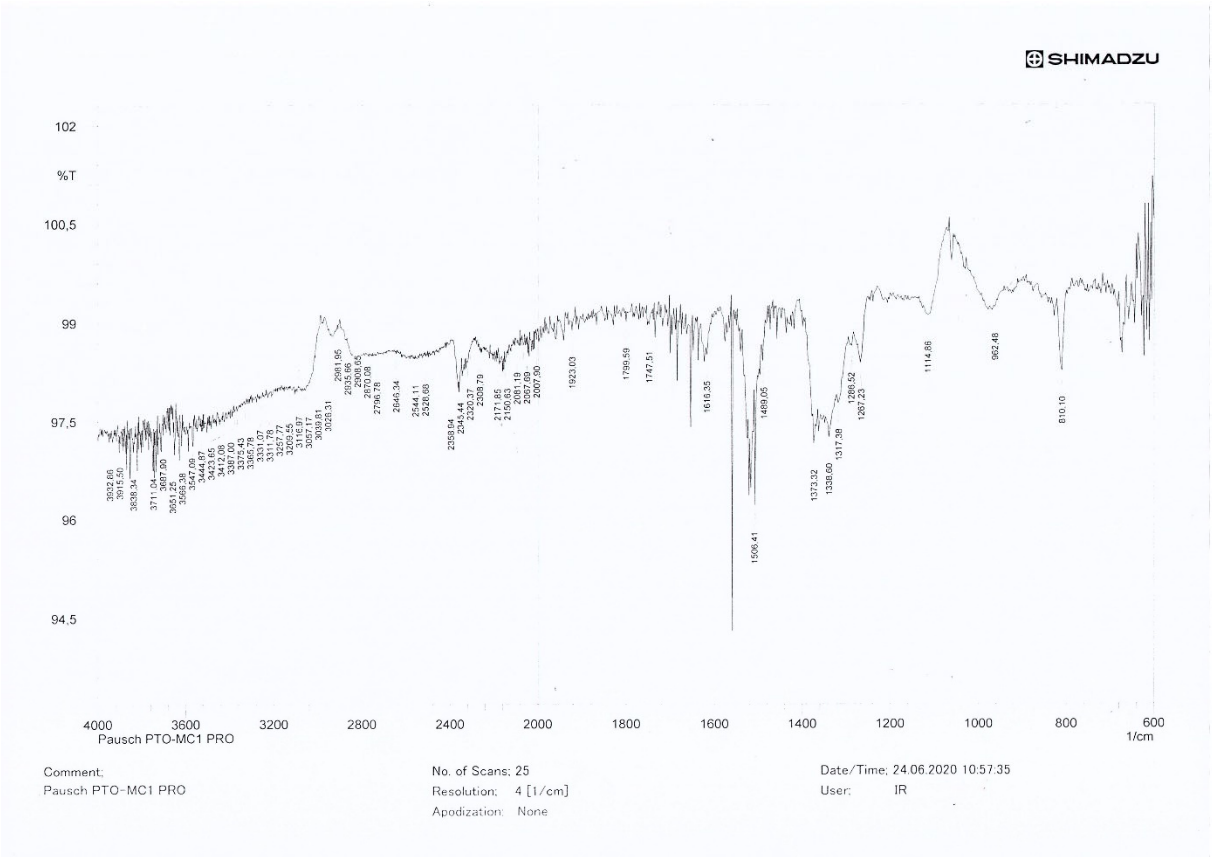

*Figure S 75: IR spectrum of **2•(progesterone)**.*

Comparison of spectra of **2•**(**ibuprofen**)$_2$ & **1a•**(**ibuprofen**)$_2$ before sonication (mid) and **1a•**(**ibuprofen**)$_2$ after sonication

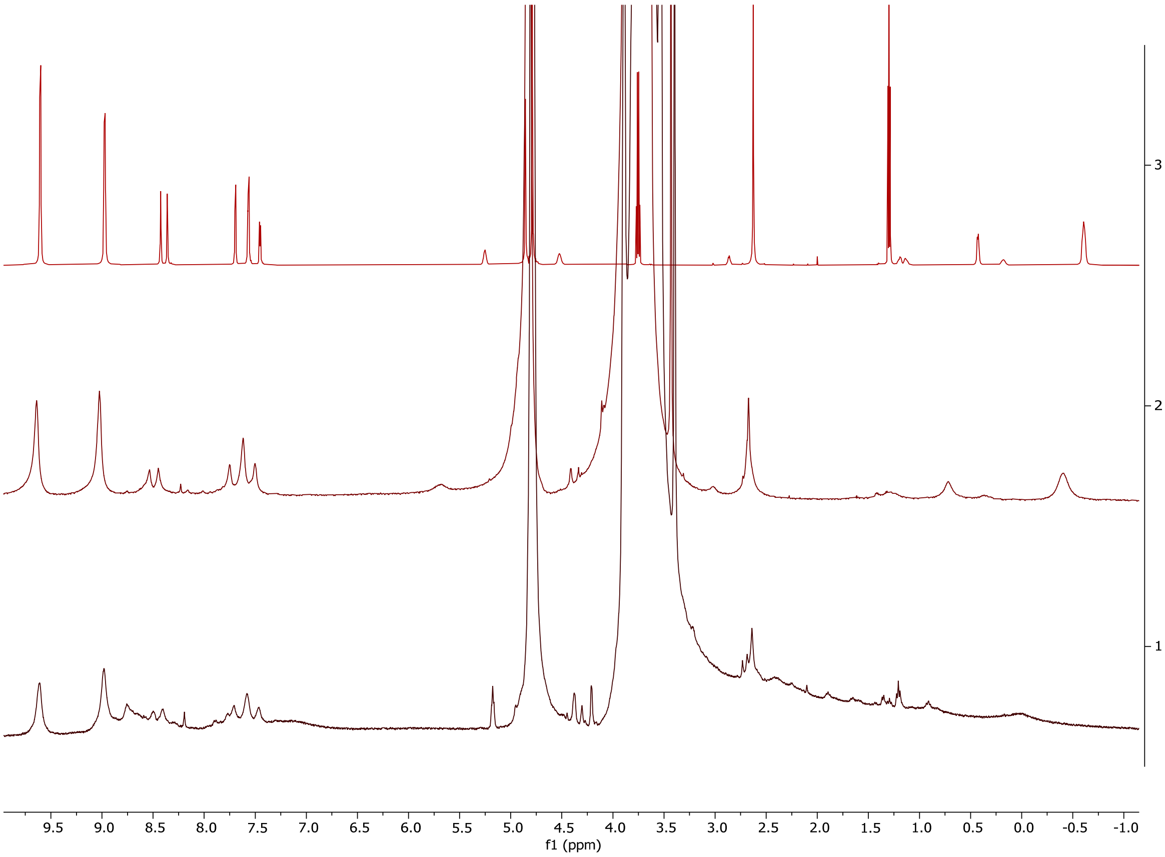

*Figure S 76: ^{1}H NMR: **2•(ibuprofen)**$_2$ (top), **1a•(ibuprofen)**$_2$ before sonication (mid), **1a•(ibuprofen)**$_2$ after sonication (bottom).*

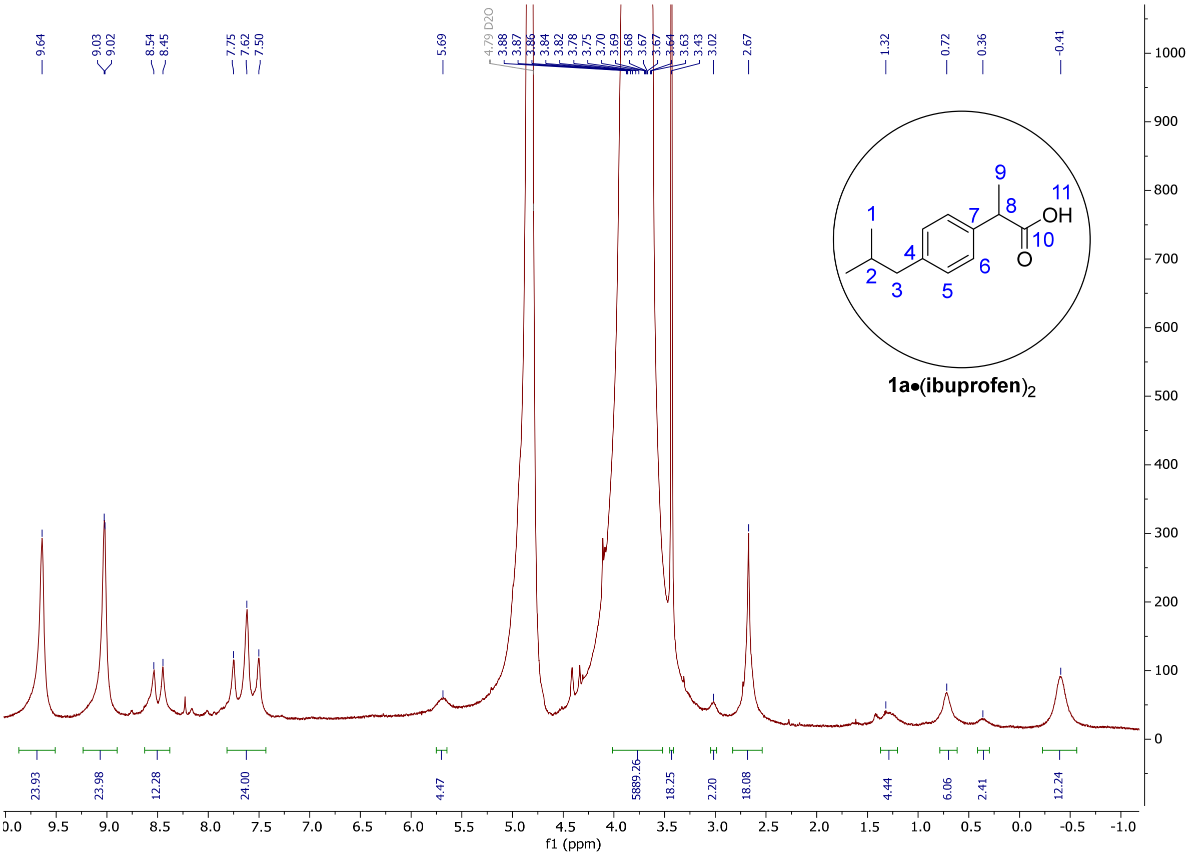

*Figure S 77: ¹H NMR (600 MHz, D_2O) of **1a•(ibuprofen)**$_2$ before sonication.*

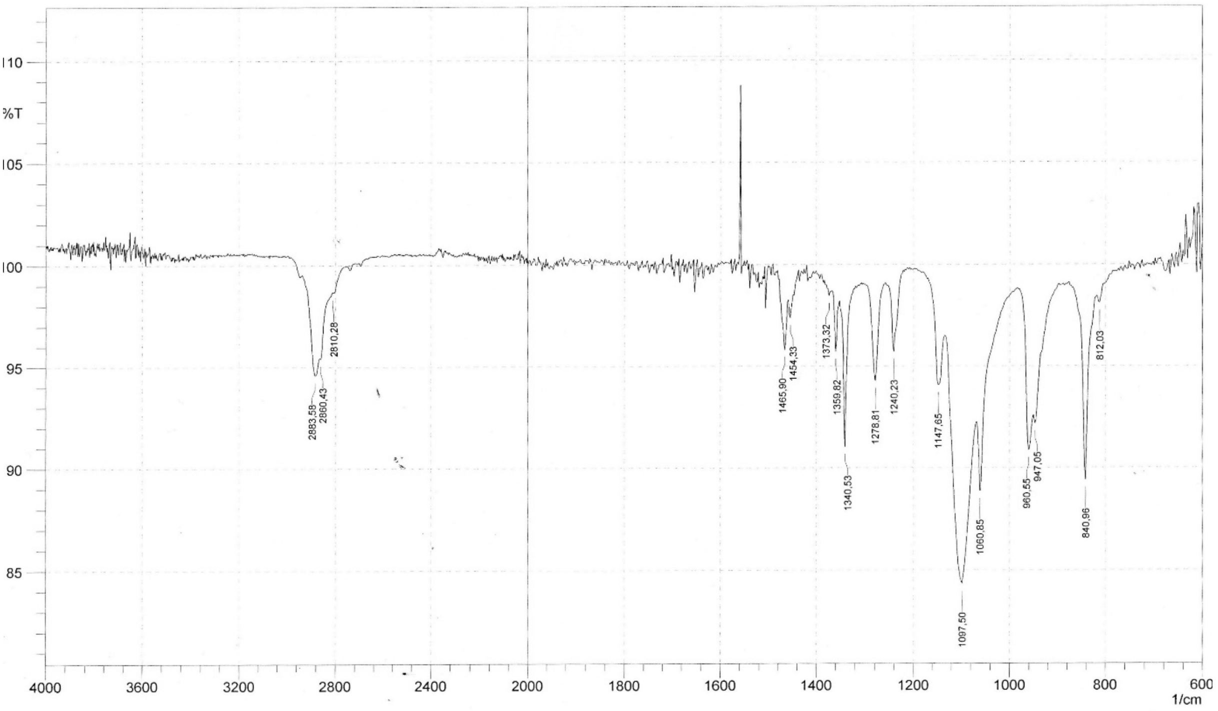

*Figure S 78: IR spectrum of **1a•(ibuprofen)**$_2$ before sonication.*

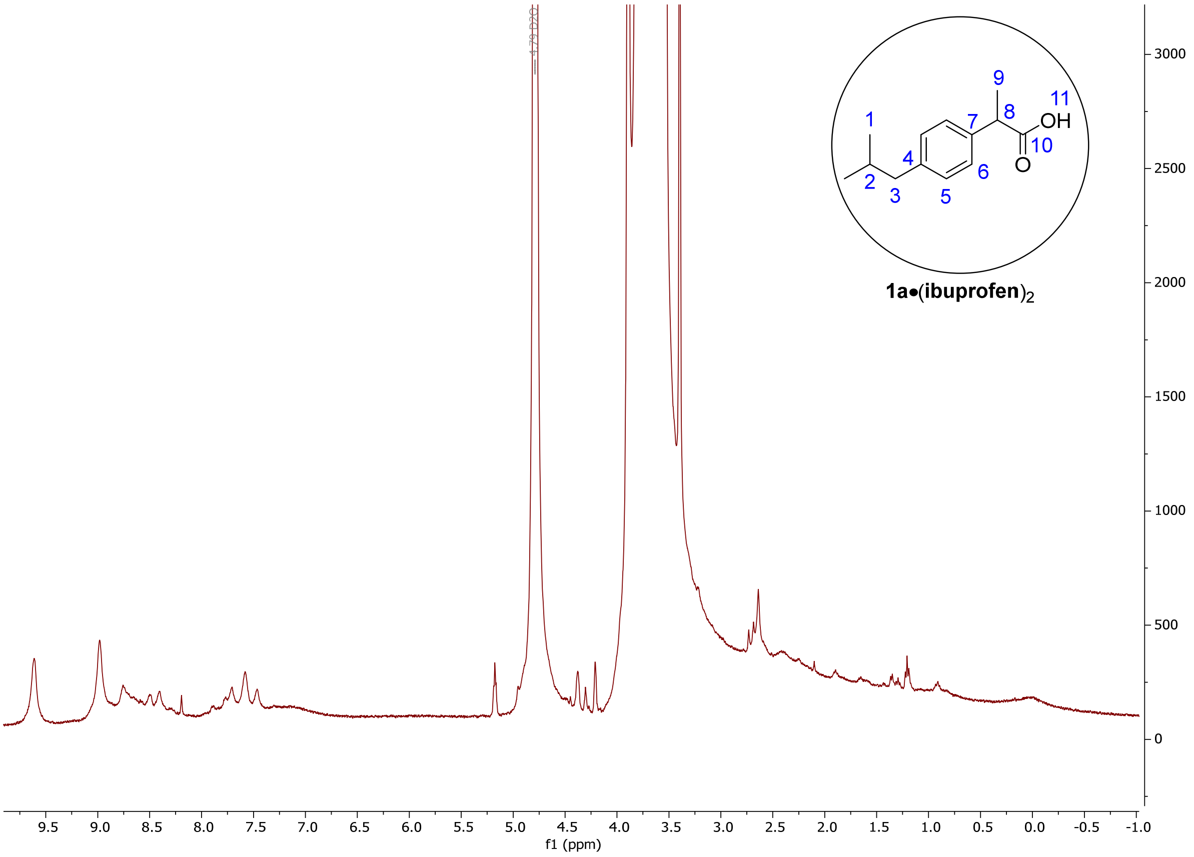

*Figure S 79: ¹H NMR (400 MHz, D_2O) of **1a•(ibuprofen)**$_2$ after sonication.*

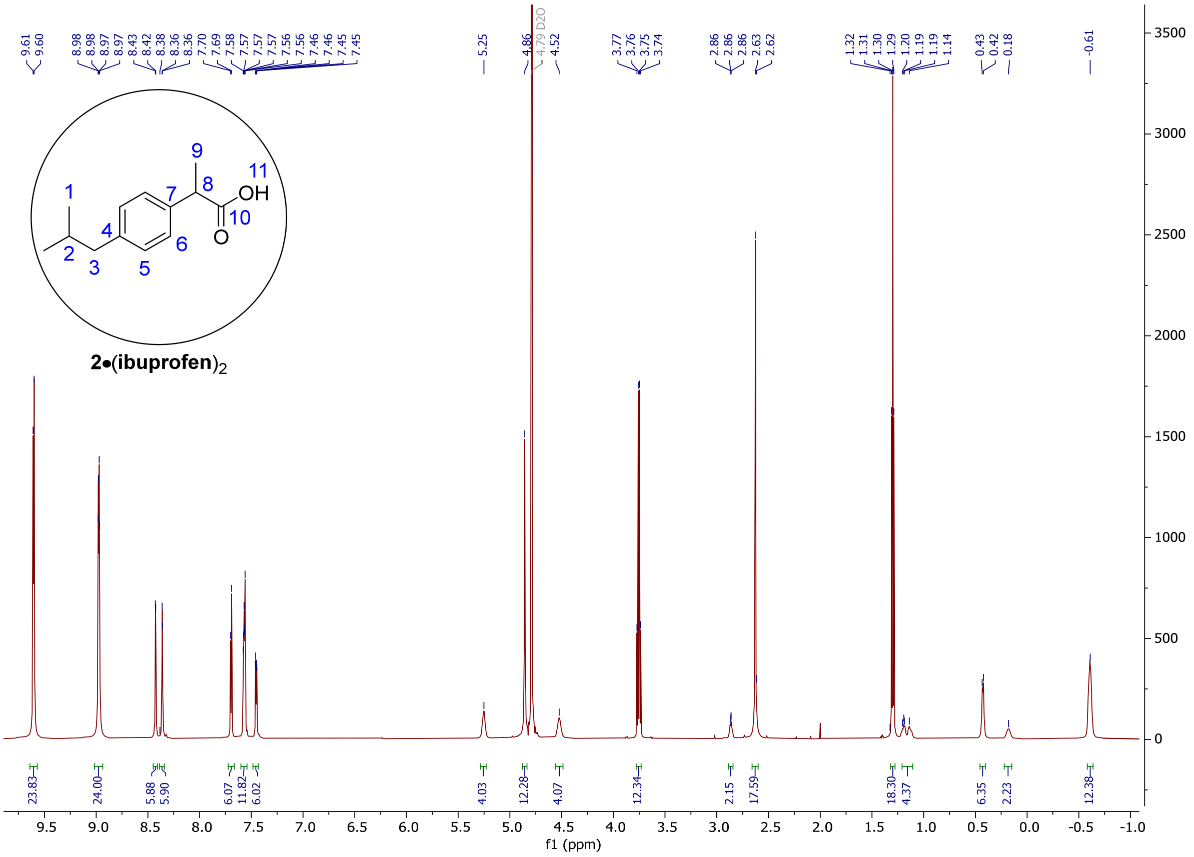

*Figure S 80: ¹H NMR (600 MHz, D_2O) of **2•(ibuprofen)**$_2$.*

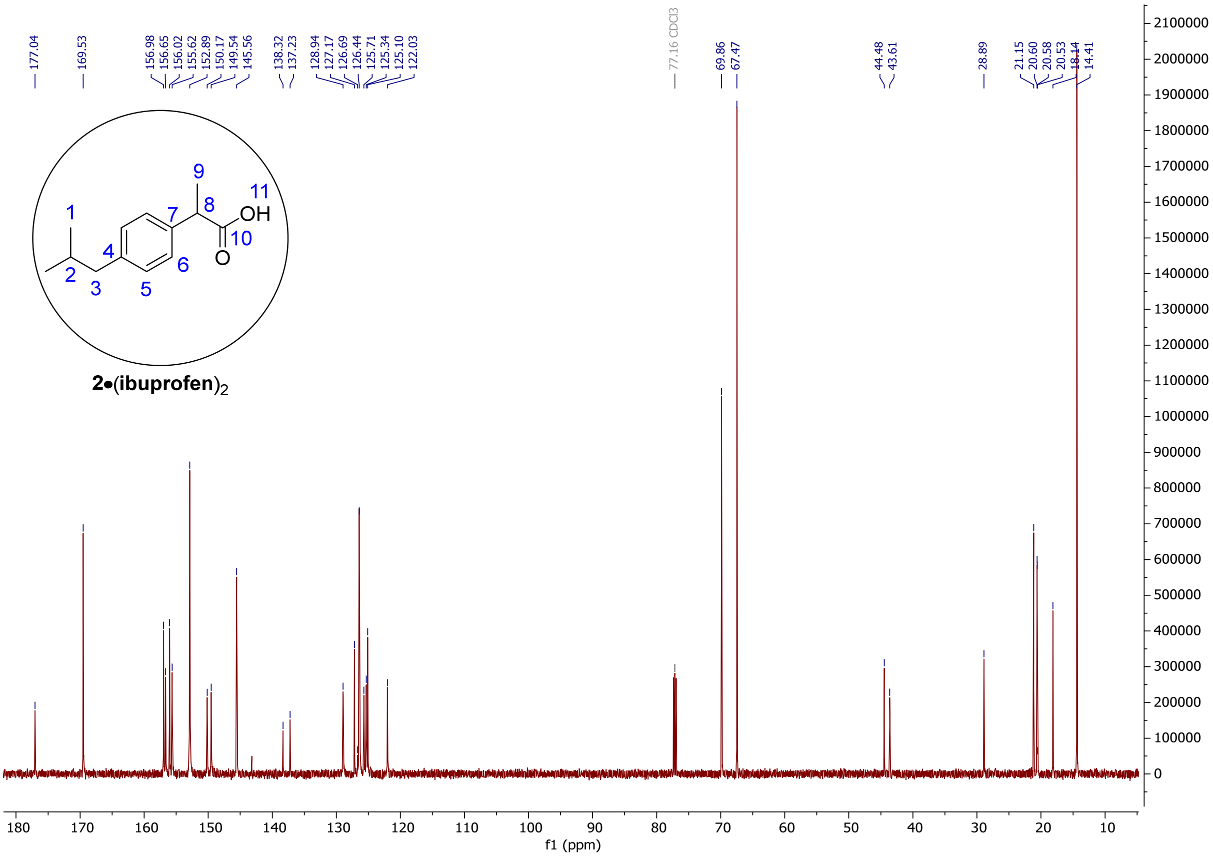

*Figure S 81: $^{13}C\{^{1}H\}$ NMR (151 MHz, D_2O) of **2•(ibuprofen)**$_2$.*

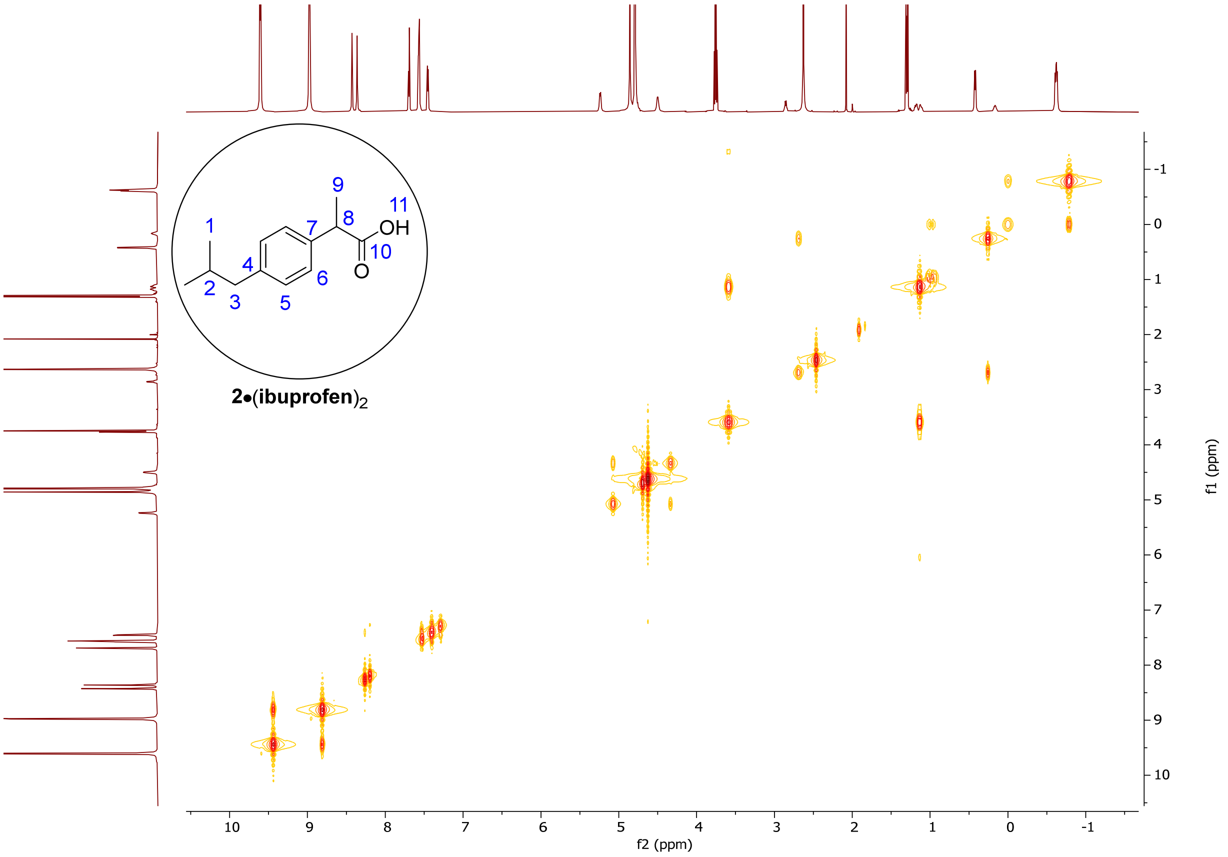

*Figure S 82: ^{1}H-^{1}H COSY NMR (600 MHz, D_2O) of **2•(ibuprofen)**$_2$.*

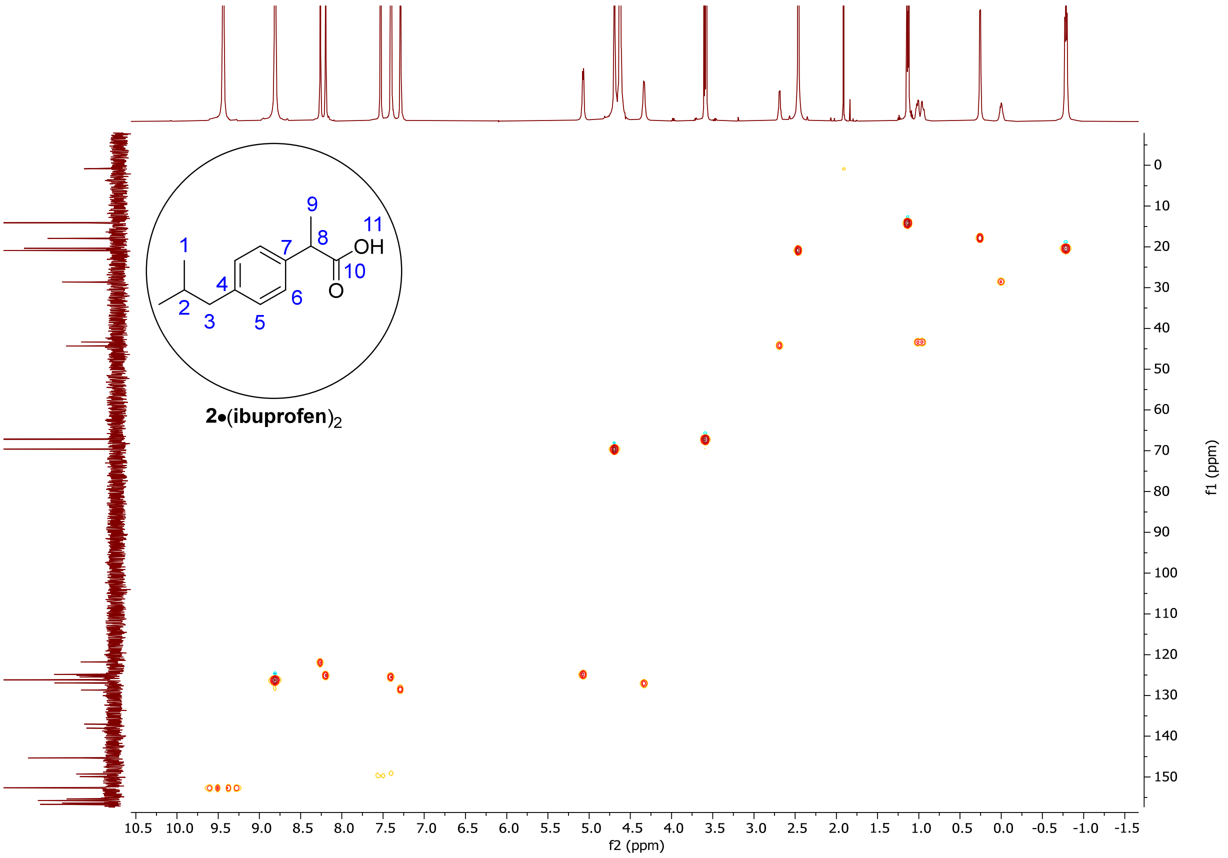

*Figure S 83: ^{1}H-{^{13}C} HSQC NMR (600 MHz, D_2O) of **2•(ibuprofen)**$_2$.*

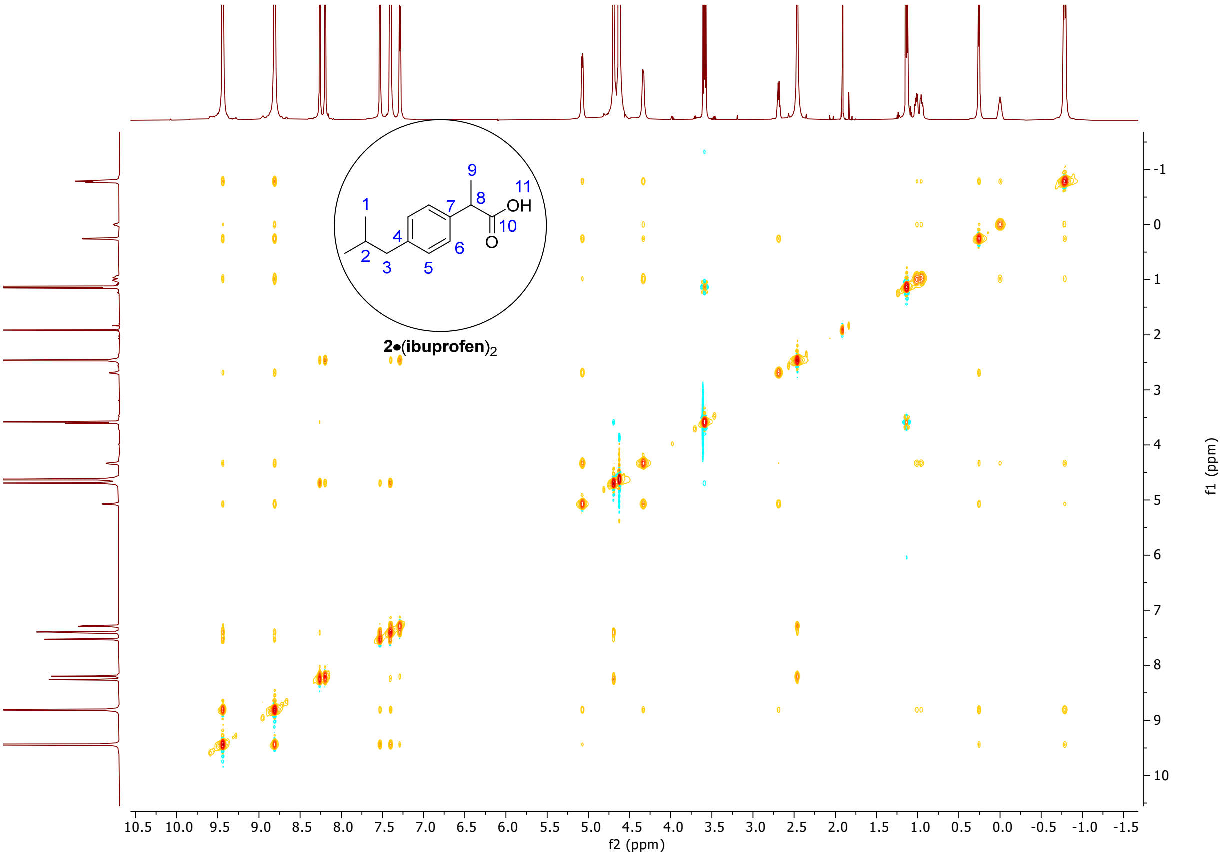

*Figure S 84: ^1H NOESY NMR (600 MHz, D_2O) of **2•(ibuprofen)$_2$**.*

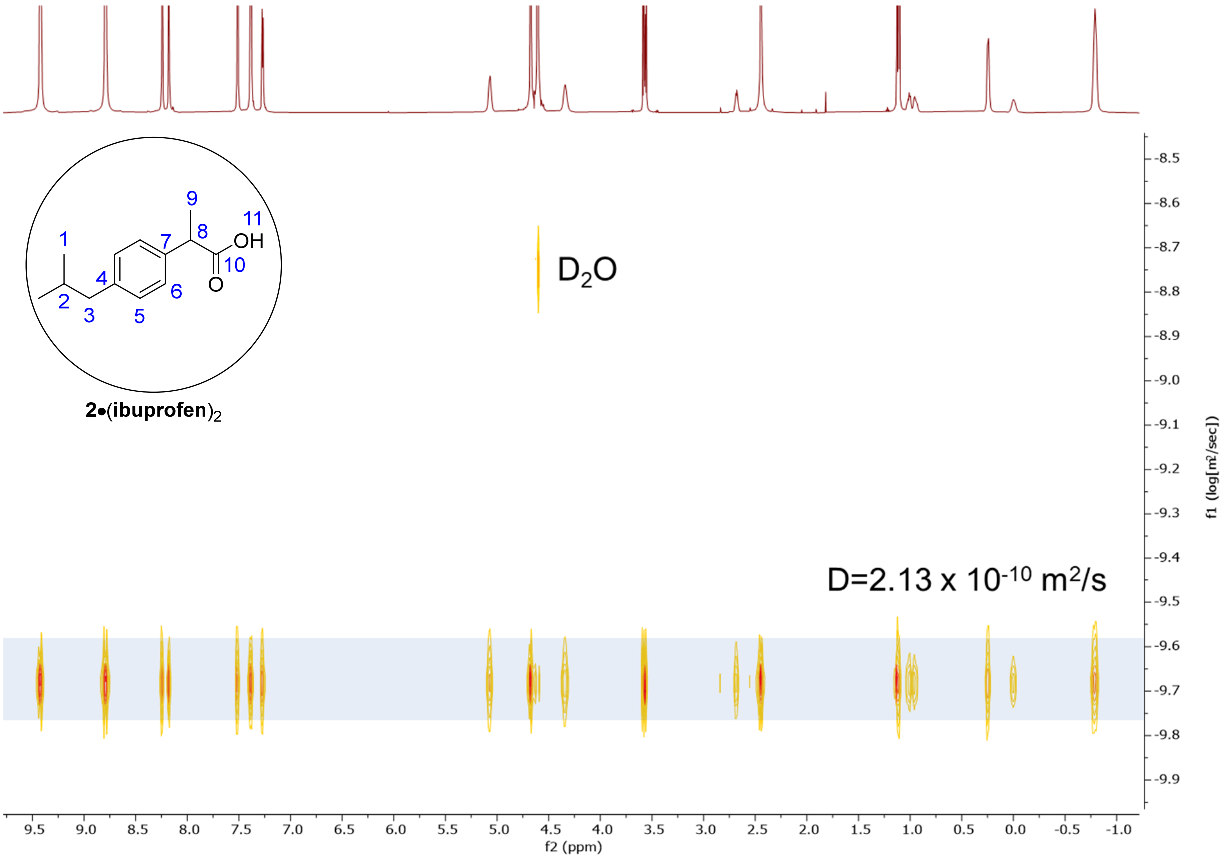

*Figure S 85: DOSY NMR (600 MHz, D_2O) of **2•(ibuprofen)$_2$**.*

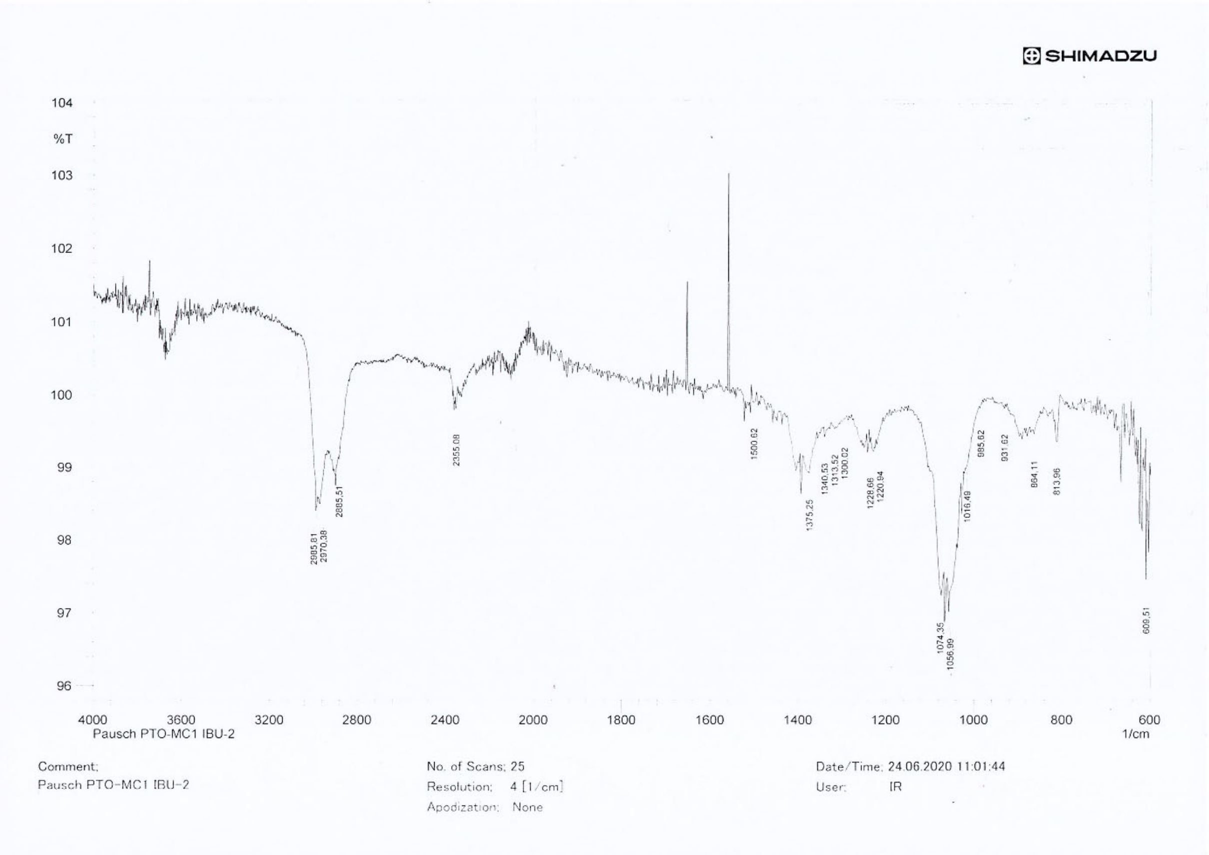

*Figure S 86: IR spectrum of **2•(ibuprofen)**$_2$.*

Phenolphthalein

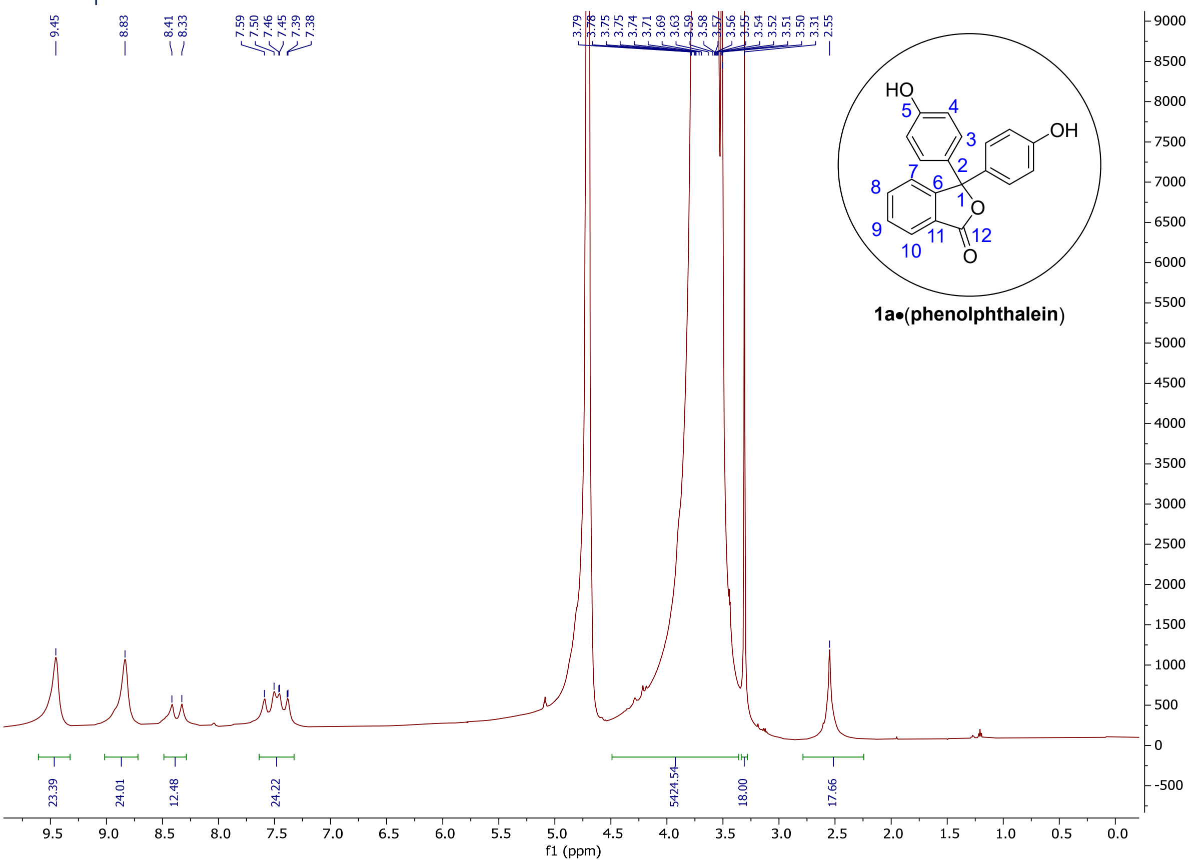

*Figure S 87: ^{1}H NMR (600 MHz, D_2O) of **1a•(phenolphthalein)**.*

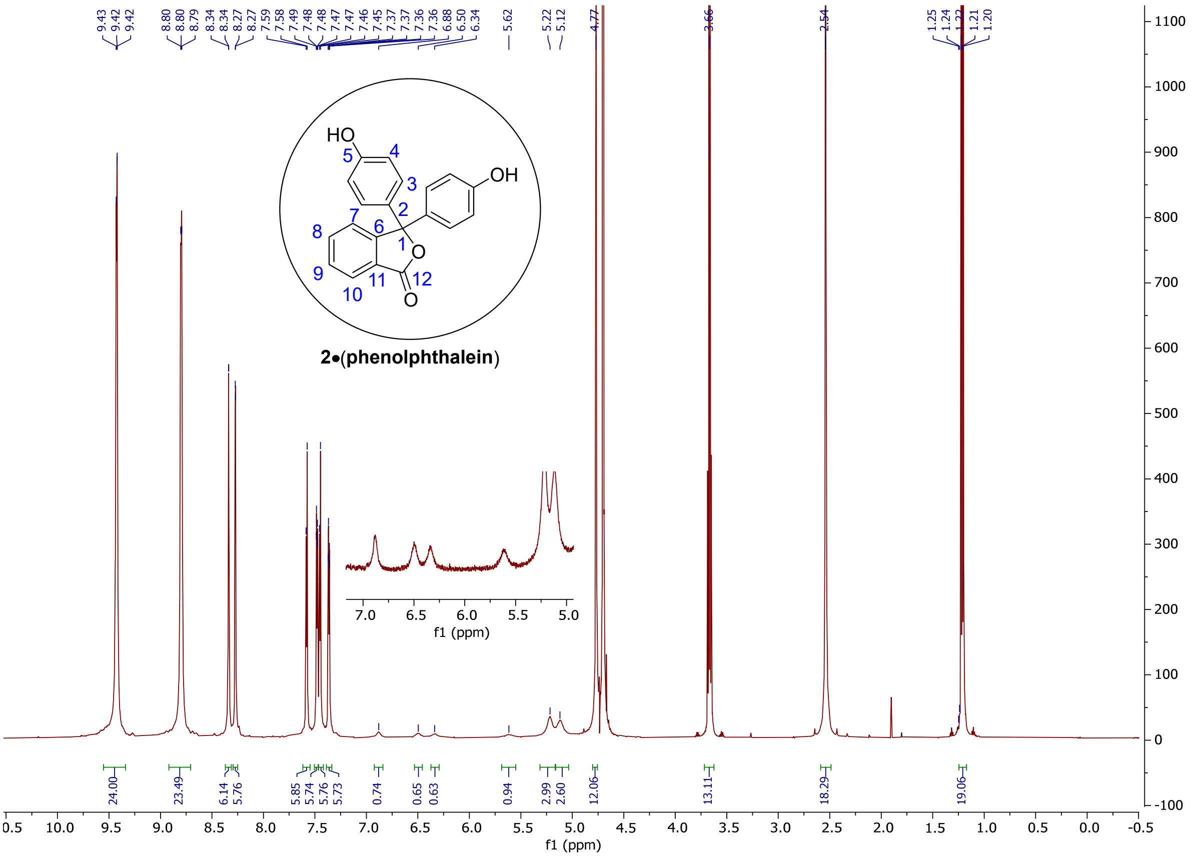

*Figure S 88: ^{1}H NMR (600 MHz, D_2O) of **2•(phenolphthalein)**.*

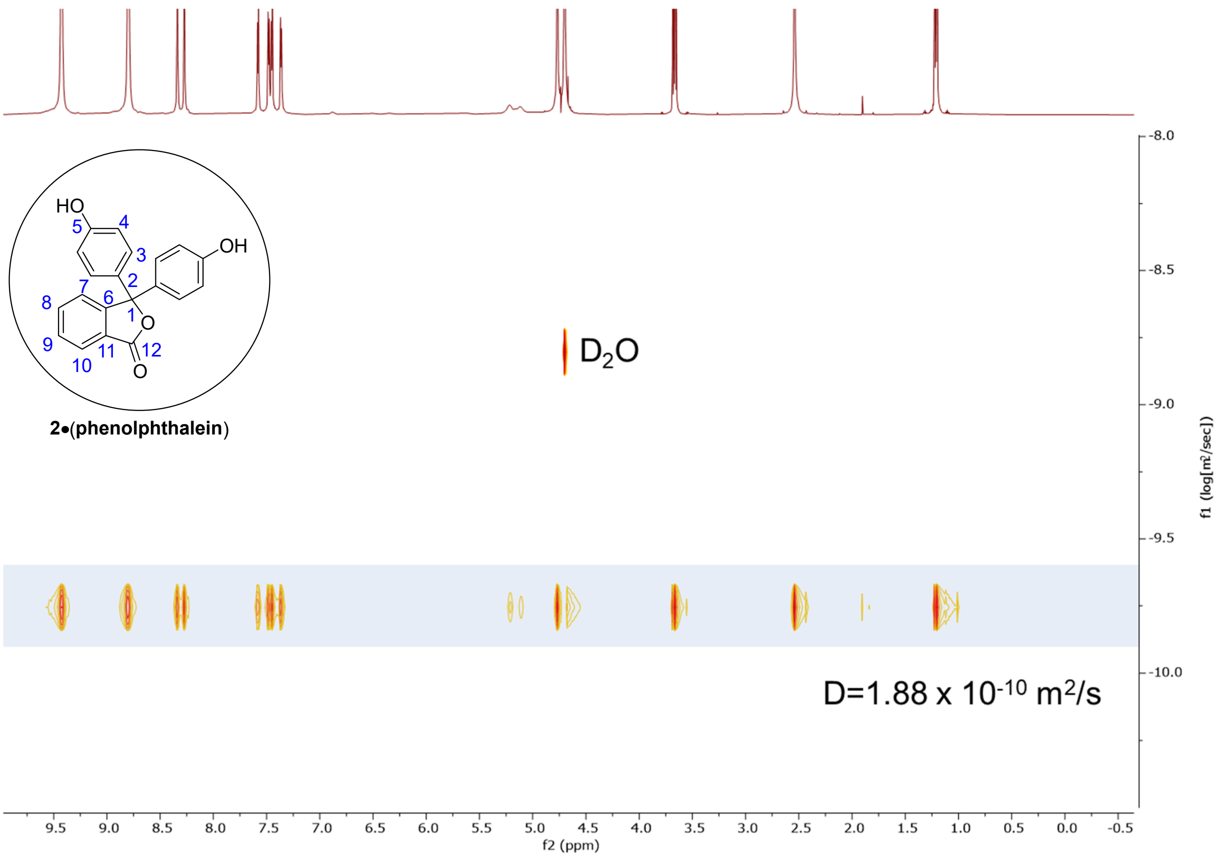

*Figure S 89: DOSY NMR (600 MHz, D_2O) of **2•(phenolphthalein)**.*

Testosterone

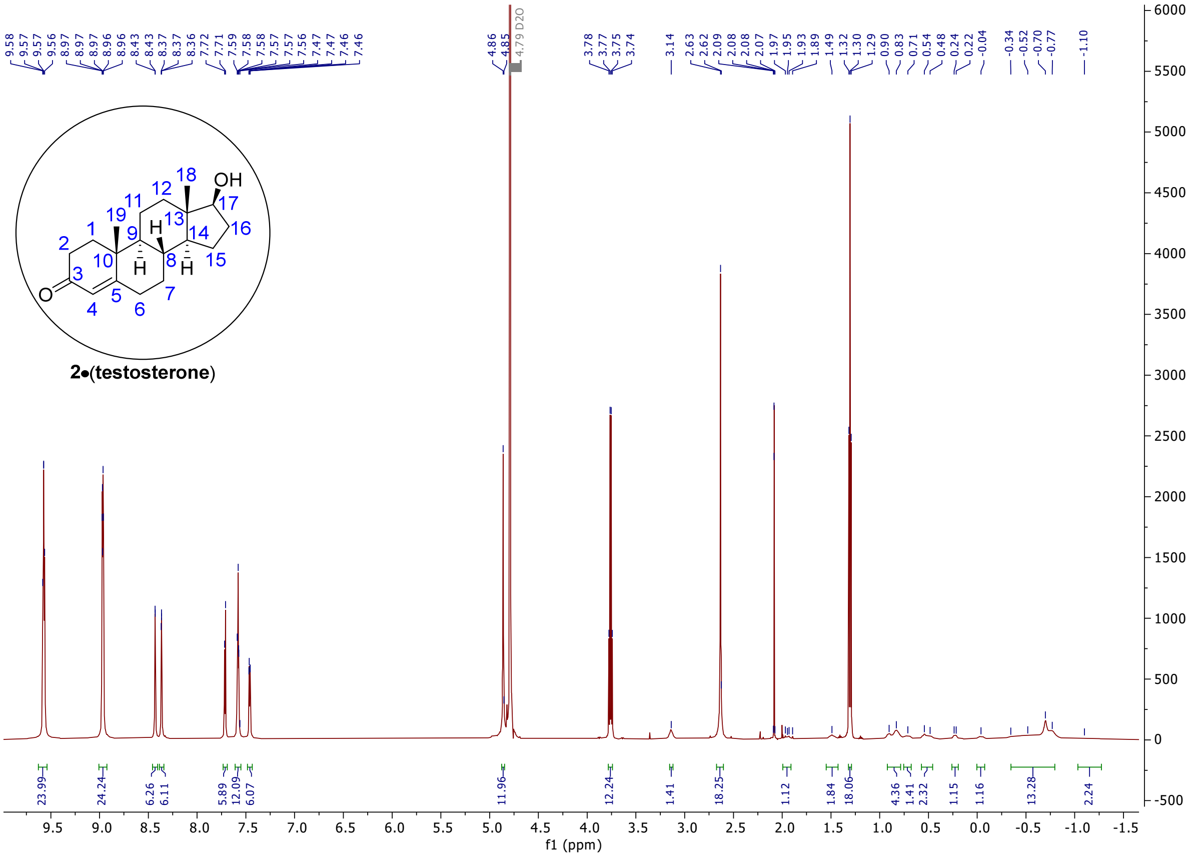

*Figure S 90: ^1H NMR (600 MHz, D_2O) of **2•(testosterone)**.*

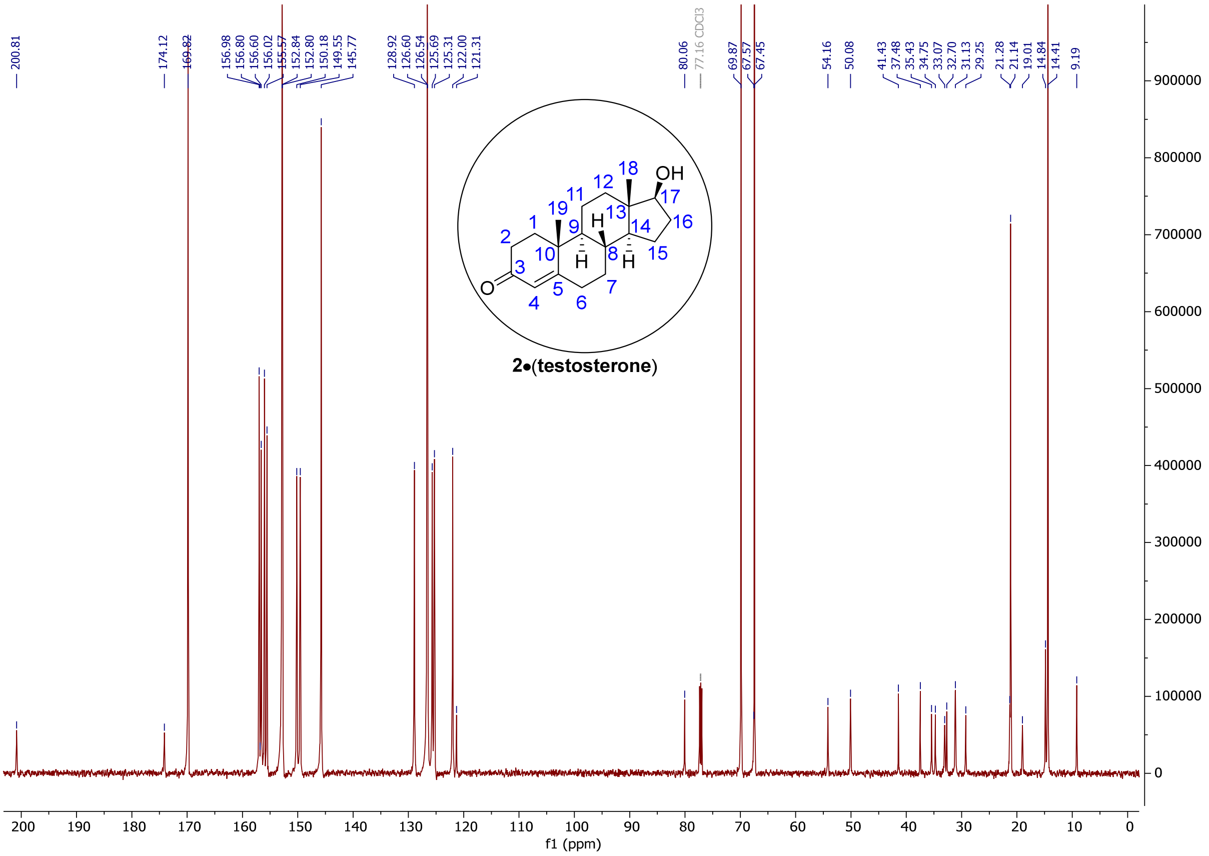

*Figure S 91: $^{13}C\{^{1}H\}$ NMR (151 MHz, D_2O) of **2•(testosterone)**.*

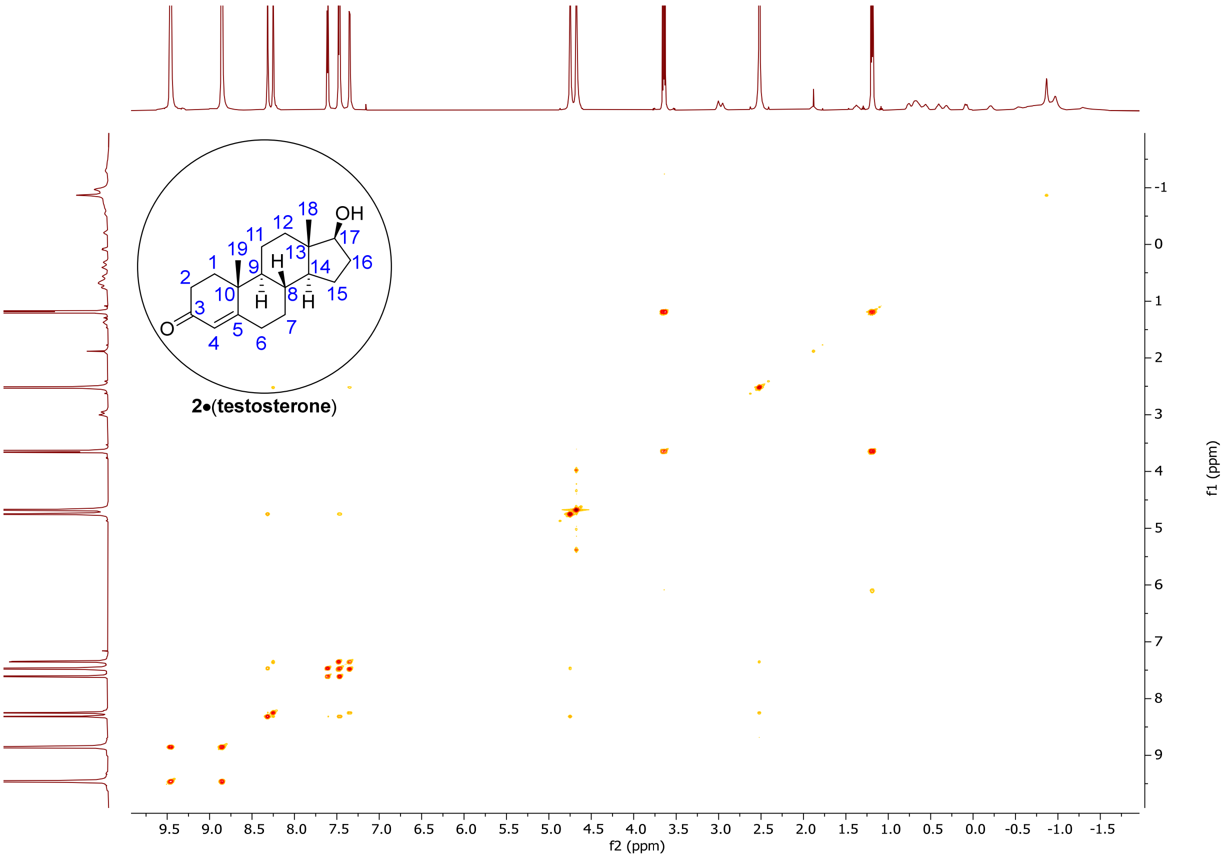

*Figure S 92: ^{1}H-^{1}H COSY NMR (600 MHz, D_2O) of **2•(testosterone)**.*

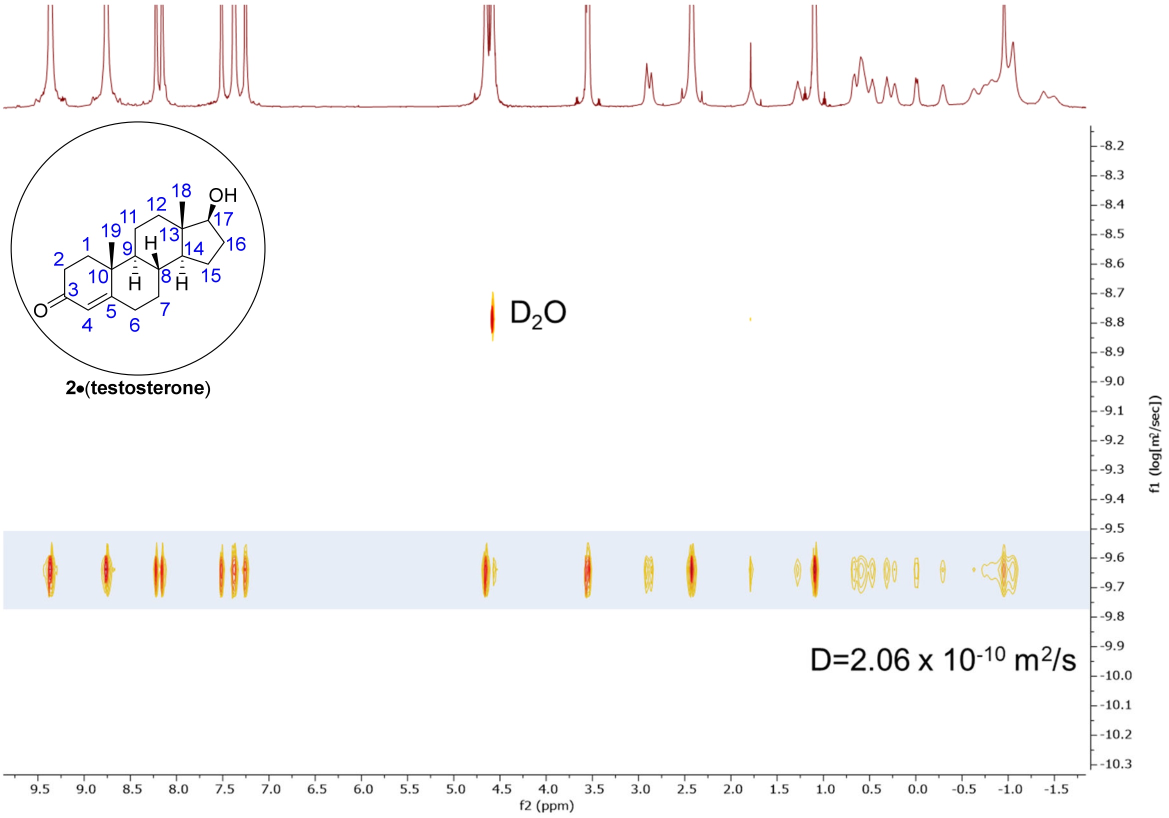

*Figure S 93: DOSY NMR (600 MHz, D_2O) of **2•(testosterone)**.*

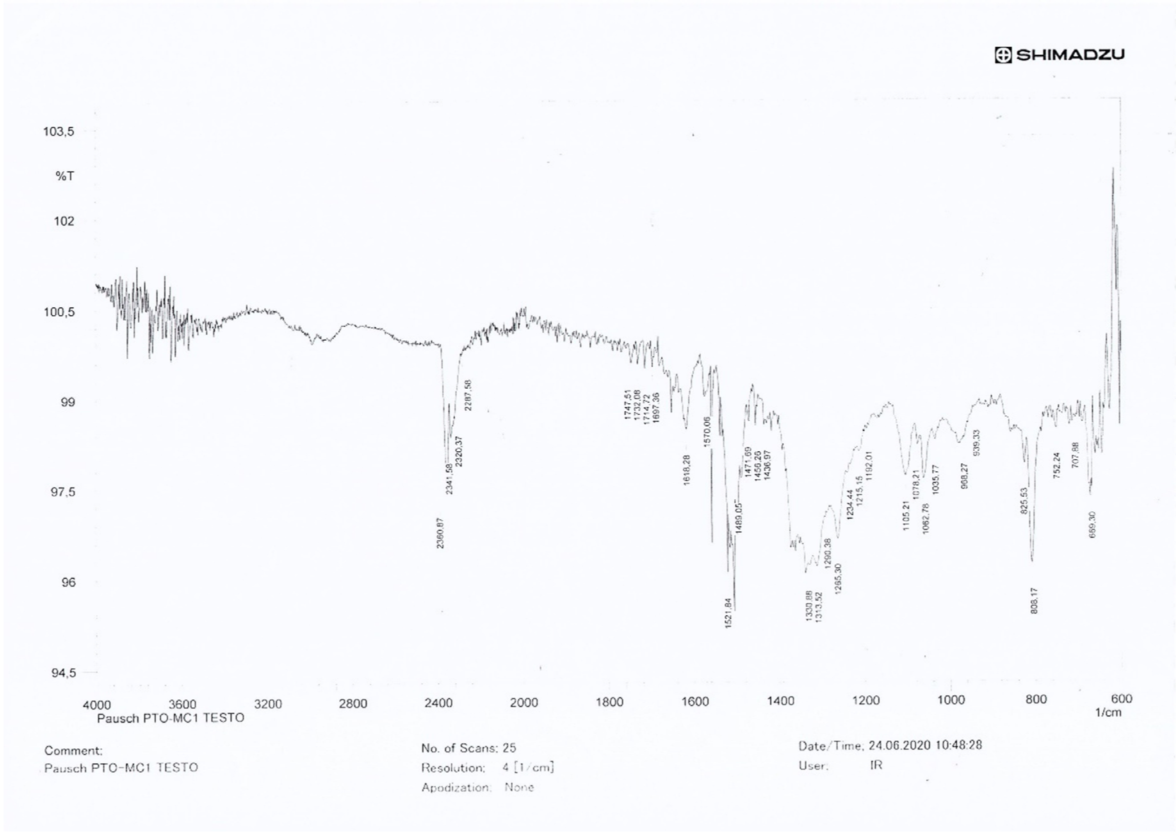

*Figure S 94: IR spectrum of **2•(testosterone)**.*

Drospirenone

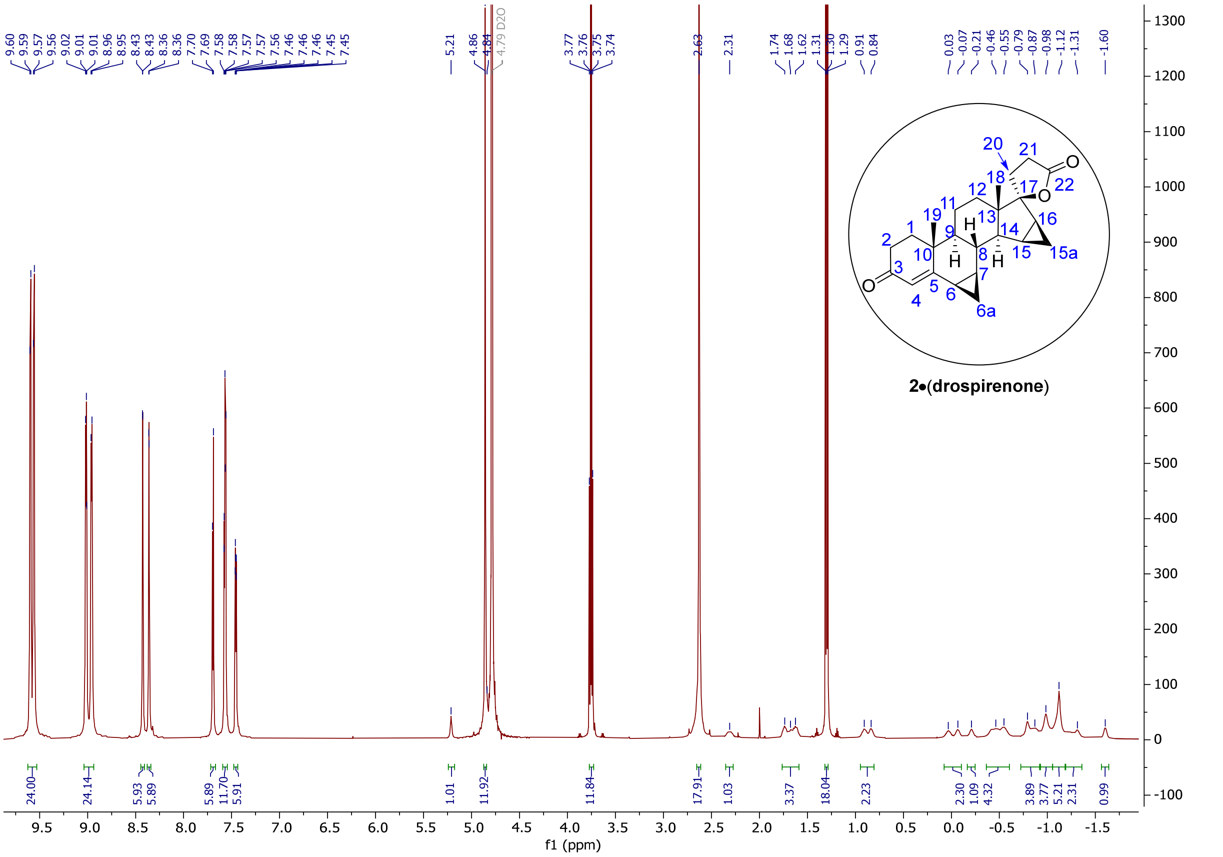

*Figure S 95: ^{1}H NMR (600 MHz, D_2O) of **2•(drospirenone)**.*

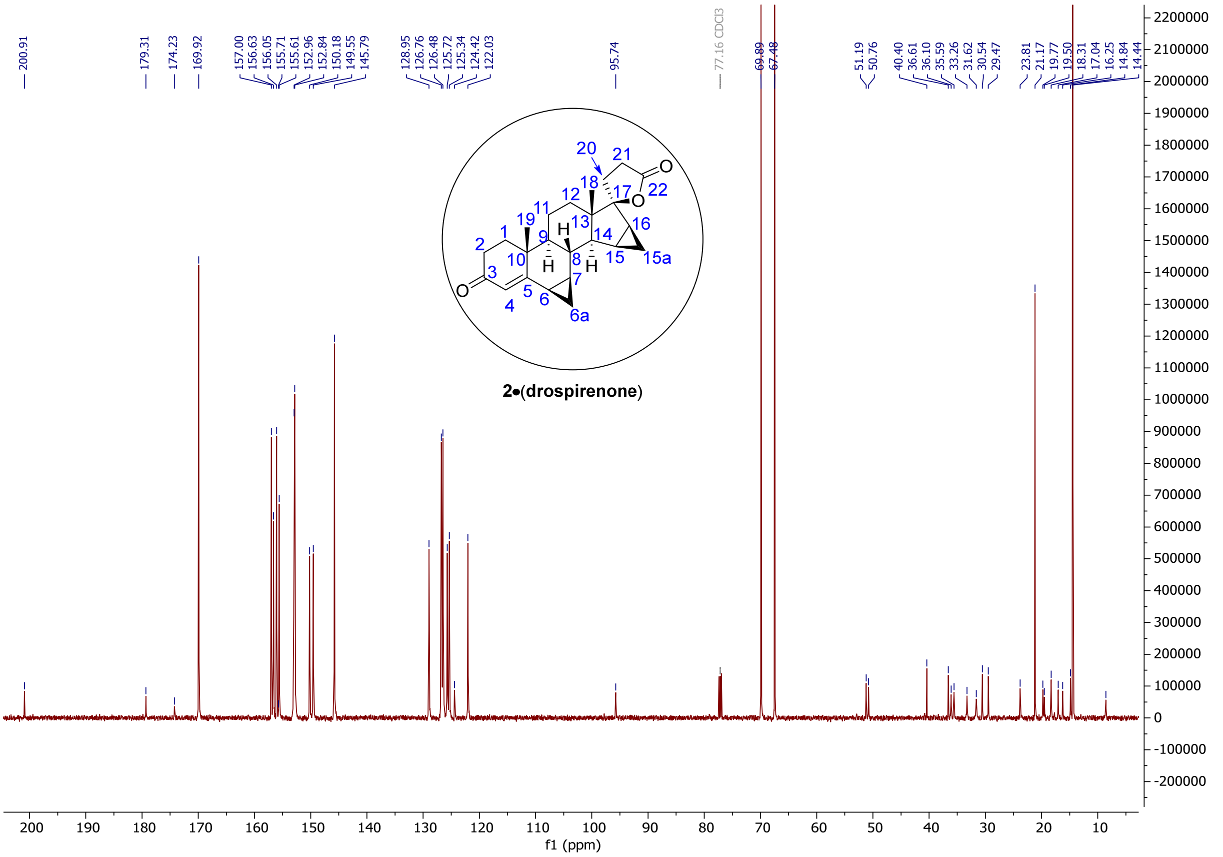

*Figure S 96: $^{13}C\{^{1}H\}$ NMR (151 MHz, D_2O) of **2•(drospirenone)**.*

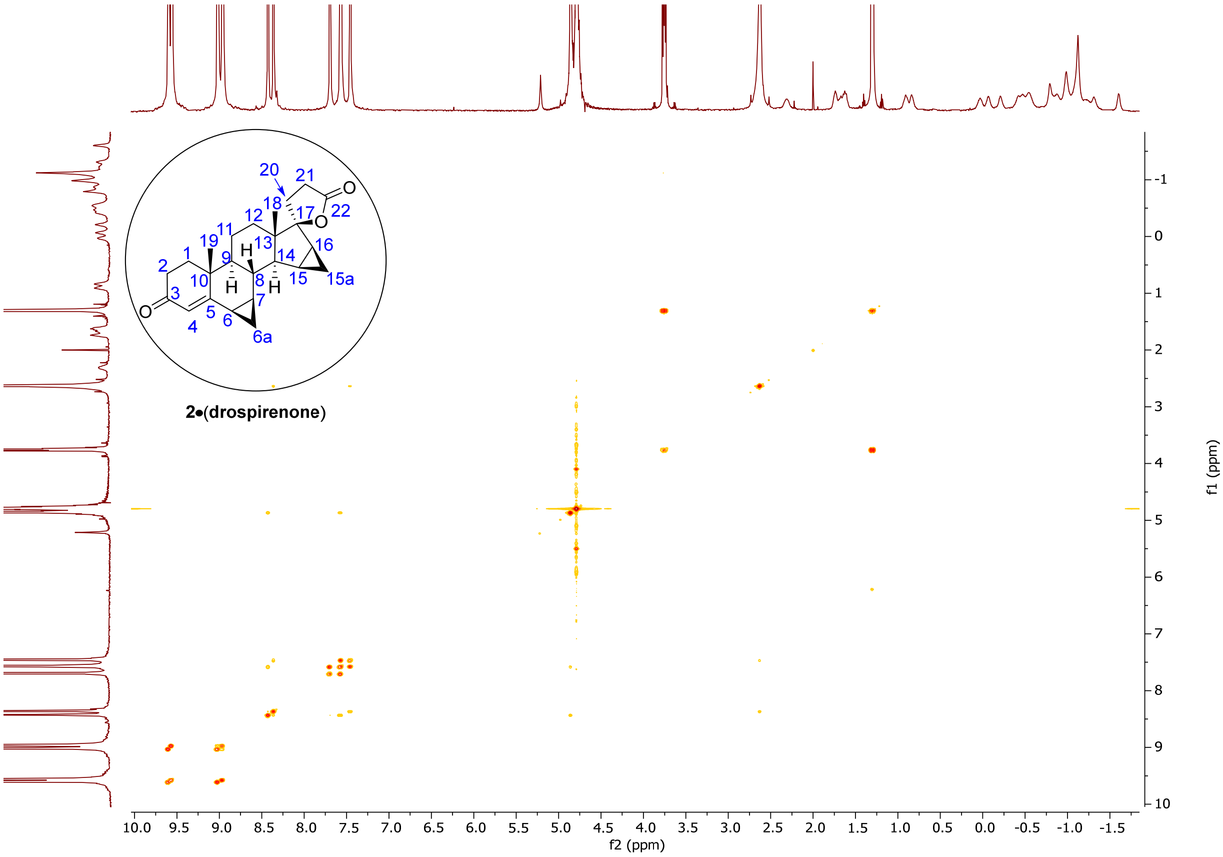

*Figure S 97: ^{1}H-^{1}H COSY NMR (600 MHz, D_2O) of **2•(drospirenone)**.*

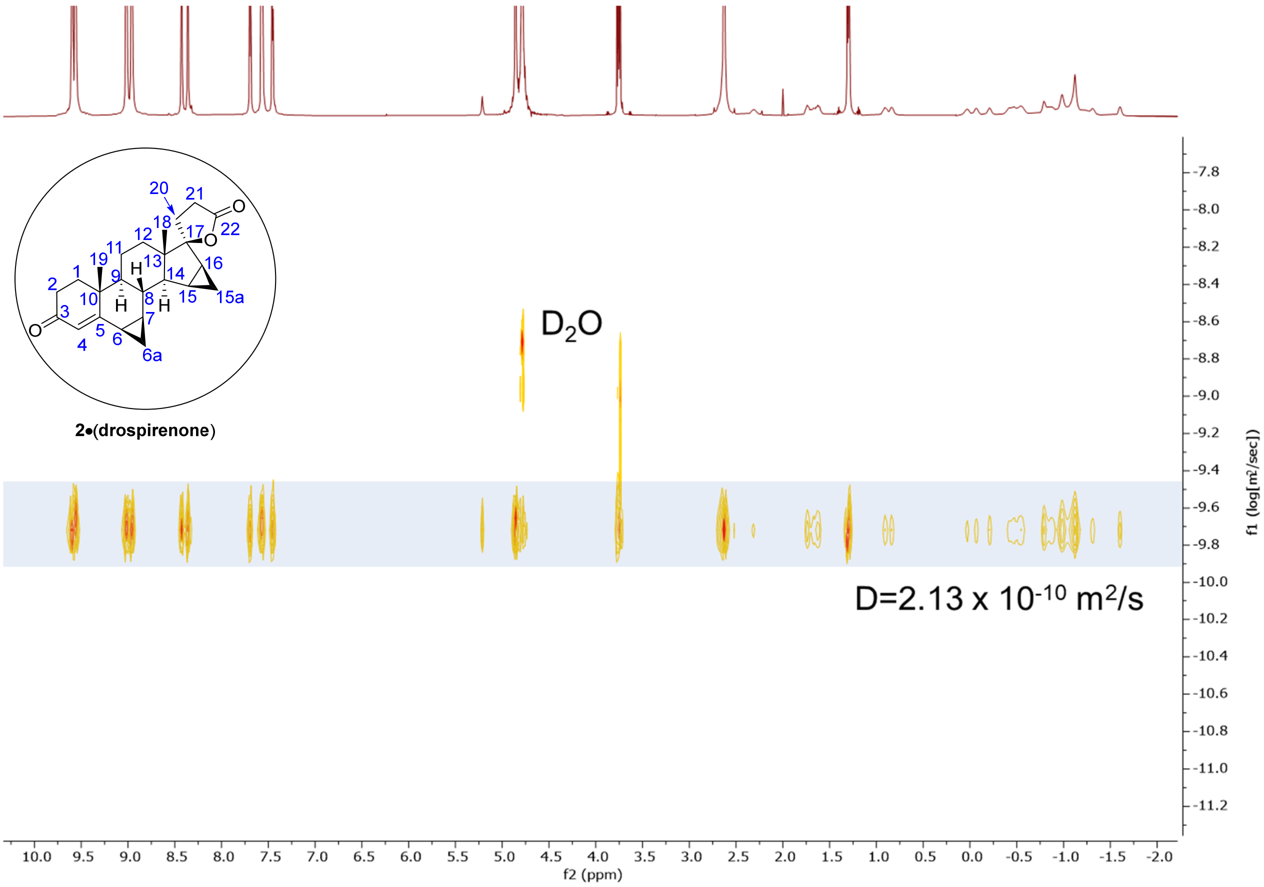

*Figure S 98: DOSY NMR (600 MHz, D_2O) of **2•(drospirenone)**.*

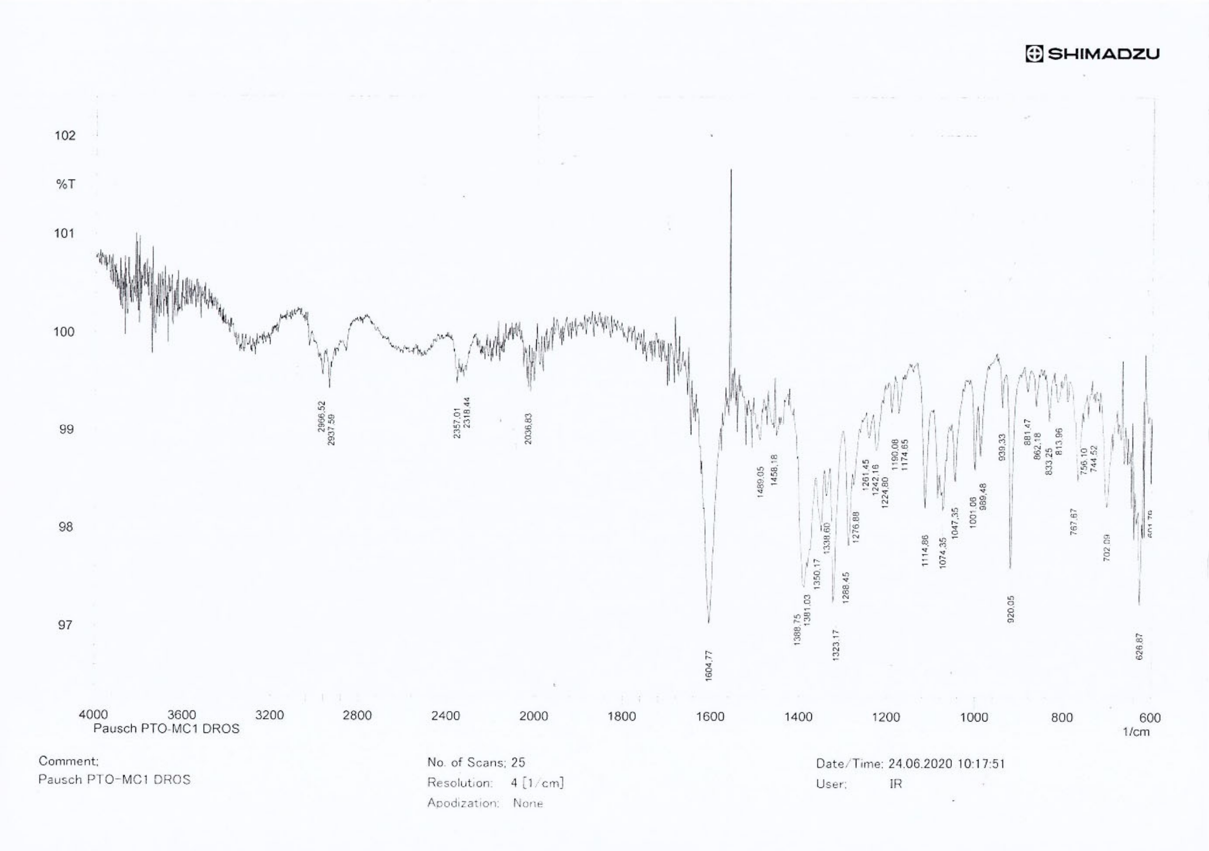

*Figure S 99: IR spectrum of **2•(drospirenone)**.*

References

[1] J. T. Edward, *J. Chem. Educ.* **1970**, *47*, 261-270.

[2] S. H. A. M. Leenders, R. Becker, T. Kumpulainen, B. de Bruin, T. Sawada, T. Kato, M. Fujita, J. N. H. Reek, *Chem. Eur. J.* **2016**, *22*, 15468-15474.

[3] A. T. Dickschat, F. Behrends, S. Surmiak, M. Weiß, H. Eckert, A. Studer, *Chem. Commun.* **2013**, *49*, 2195-2197.

[4] K. Hayamizu, O. Kamo, *Magn. Reson. Chem.* **1990**, *28*, 250-256.

[5] P. G. Quintana, S. M. Romero, G. Vaamonde, A. Baldessari, *J. Mol. Catal. B Enzym*. **2013**, *97*, 110-117.
